# Food systems transformation would reshape global agriculture

**DOI:** 10.1038/s41586-026-10775-2

**Published:** 2026-07-15

**Authors:** Matthew Gibson, Marina Sundiang, Daniel Mason-D’Croz, Thais Diniz Oliveira, Felicitas Beier, Lauren Benavidez, Astrid Bos, Maksym Chepeliev, Jonathan Doelman, Shahnila Dunston, Shinichiro Fujimori, Tomoko Hasegawa, Petr Havlik, Jordan Hristov, Jonas Jägermeyr, Marta Kozicka, Marijke Kuiper, Page Kyle, Thijs de Lange, Benjamin Leon Bodirsky, Hermann Lotze-Campen, Hermen Luchtenbelt, David Meng-Chuen Chen, Abhijeet Mishra, Christoph Müller, Gerald Nelson, Amanda Palazzo, Ignacio Perez Dominguez, Alexander Popp, Ronald Sands, Marco Springmann, Elke Stehfest, Timothy B. Sulser, Kiyoshi Takahashi, Gianmaria Tassinari, Ferike Thom, Philip Thornton, Kazuaki Tsuchiya, Willem-Jan van Zeist, Hans van Meijl, Dominique van der Mensbrugghe, Detlef Van Vuuren, Hannah H. E. van Zanten, Isabelle Weindl, Keith Wiebe, Xin Zhao, Mario Herrero

**Affiliations:** 1 Ashley School of Global Development and the Environment, Cornell University, Ithaca, NY USA; 2 Centre on Climate Change and Planetary Health, London School of Hygiene & Tropical Medicine, London, UK; 3 Cornell Atkinson Centre for Sustainability, Cornell University, Ithaca, NY USA; 4 Agricultural Economics and Rural Policy Group, Wageningen University & Research, Wageningen, The Netherlands; 5 Potsdam Institute for Climate Impact Research (PIK), Member of the Leibniz Association, Potsdam, Germany; 6 Center for Global Trade Analysis, Purdue University, West Lafayette, IN USA; 7 Netherlands Environmental Assessment Agency (PBL), The Hague, The Netherlands; 8 International Food Policy Research Institute (IFPRI), Washington, DC USA; 9 Kyoto University, Kyoto, Japan; 10 Ritsumeikan University, Kusatsu, Japan; 11 International Institute for Applied Systems Analysis (IIASA), Laxenburg, Austria; 12 European Commission Joint Research Centre (JRC), Seville, Spain; 13 Climate School, Columbia University, New York, NY USA; 14 NASA Goddard Institute for Space Studies, NASA, New York, USA; 15 Wageningen Social and Economic Research, Wageningen University & Research, Wageningen, The Netherlands; 16 Pacific Northwest National Laboratory, College Park, MD USA; 17 Humboldt-Universität zu Berlin (HU), Albrecht Daniel Thaer-Institut für Agrar- und Gartenbauwissenschaften, Berlin, Germany; 18 Integrative Research Institute on Transformations of Human–Environment Systems (IRI THESys), Humbolt University Berlin, Berlin, Germany; 19 University of Illinois Urbana-Champaign, Champaign, IL USA; 20 Faculty of Organic Agricultural Sciences, University of Kassel, Witzenhausen, Germany; 21 US Department of Agriculture, Washington, DC USA; 22 University College London, London, UK; 23 Environmental Change Institute, Oxford University, Oxford, UK; 24 National Institute for Environmental Studies, Tsukuba, Japan; 25 Johann Heinrich von Thünen Institute, Braunschweig, Germany; 26 International Livestock Research Institute (ILRI), Nairobi, Kenya; 27 Farming Systems Ecology Group, Wageningen University & Research, Wageningen, The Netherlands

**Keywords:** Agriculture, Economics, Environmental impact, Socioeconomic scenarios, Sustainability

## Abstract

Food systems are a major contributor to exceeding planetary boundaries^1–3^ and poor quality diets are a key mortality risk globally^4^. Projected population and income growth could exacerbate these challenges^5^. In response, there are calls for transformation towards healthy and sustainable food systems^6–8^. However, the scale and distribution of the impacts of this transformation on agriculture are underexplored. Here we show that, by 2050, the transformation of food systems towards healthy diets (adoption of the EAT–*Lancet* reference diet), improved productivity and halving of food waste results in a fundamental restructuring of global agriculture, aspects of which break with historical trends. Scenario simulations using a multimodel ensemble of ten global economic models show a 6% median decrease in agricultural land (+1% to −26%) compared with 2020 levels. By 2050, agricultural production would be 17% lower than business-as-usual projections (−2% to −32%) and, economically, the value of this production is US$1.6 trillion (26%) lower (+8% to −58%). Within this, the value of livestock production would be substantially lower than current 2050 projections (−49% to −83%), while vegetable, fruit, nut and legume production value would increase by 23% (−33% to +106%). Results are dependent on the assumed policies to achieve the transformation scenario. We highlight a more active role for food policy to consider the benefits of such a transformation (improved population health and reduced environmental pressures) and navigate the political economy of its impacts.

## Abstract (web summary)

Scenario simulations using global economic models show that by 2050 the transformation of food systems towards healthy diets, improved productivity and halving of food waste result in a restructuring of global agriculture.

## Main

Poor-quality diets are one of the leading mortality risks globally^4^. Food systems (that is, all elements and activities related to the production, processing, distribution, preparation and consumption of food, their governance and outcomes of these activities^9^) contribute around one-third of all anthropogenic greenhouse gas (GHG) emissions globally^1^ and are a leading factor in exceeding several planetary boundaries^2,10^. In response to these intersecting crises, there are calls to transform food systems^7,8,11,12^. However, the scale and distribution (by sector and region) of the impacts of this transformation on agriculture are underexplored.

Specifically, our review of food systems modelling literature found 75 studies that have conducted scenario and modelling analysis of food systems change (for example, refs. ^2,13–16^) (Supplementary Figs. 2 and 3 and Supplementary Tables 11–13). From this, we identify several research gaps. First, sectoral disaggregation and granularity are lacking. More than half (56%) of studies either did not report a specific agricultural sector or reported a single aggregate sector, with just four studies reporting ten or more sectors. Second, although the use of historical data was present in 23% of studies, none considered data before the 1960s (owing to the use of Food and Agriculture Organization (FAO) statistics) which precludes contextualization with longer-run changes. Finally, economic analysis of changes in the value of production of different agricultural sectors under future scenarios was absent from the reviewed studies, thus preventing a more detailed exploration of sectoral economic impacts.

The potential socioeconomic and livelihood impacts stemming from food system transformation will probably mean that it is politically contested^17^. As such, it is important to understand the implications of such a food systems transformation in more detail. Specifically, given the substantial changes in food intake implied by a global shift to a healthy diet (for example, EAT-Lancet^18^) we focus on the socioeconomic and environmental impacts of food systems transformation across different sectors (for example, animal-sourced products, cereals, sugar, vegetables, fruits, nuts and legumes). This is complemented by a historical lens which helps to contextualize future projections with decadal and multicentury agricultural trends^19^.

We use a multimodel ensemble (MME) of ten global economic models (AIM, CAPRI, ENVISAGE, FARM, GCAM, GLOBIOM, IMAGE, IMPACT, MAGNET and MAgPIE; Supplementary Table 3). The ensemble comprises a mix of computable general equilibrium, partial equilibrium and integrated assessment models that have all previously participated in multimodel exercises through the Agricultural Model Intercomparison and Improvement Project (AgMIP)^20–22^. Ensemble analyses combine different modelling perspectives on ex ante scenarios not available from single model assessments^13^, with the variability of model ensemble results giving a measure of uncertainty in how a system, and its responses, are modelled^23^. We simulate two main scenarios: a business-as-usual (BAU) scenario which continues current trends to 2050 and a food systems transformation (EL2) scenario which combines 2025 EAT–*Lancet* Commission (EAT–*Lancet* 2.0 report)^6^ healthy diet recommendations (modelled as consumer preference shifts), higher supply-side productivity improvements of crops and livestock compared with BAU, and a 50% reduction in food loss and waste (FLW). The EL2 scenario is an interpretation of a food systems transformation which includes three key supply-and-demand strategies that are regarded as levers for food systems change^12^. Additionally, three scenarios simulated the individual components of EL2 (dietary change, increased productivity and FLW reduction) to assess their impacts in isolation (Supplementary Table 1). Given greater uncertainty around the representation of FLW, four more FLW sensitivity scenarios were simulated (Supplementary Fig. 6). Data inputs to the modelling ensemble include the latest updated macroeconomic and demographic projections to the shared socioeconomic pathways (SSPs)^5^.

Unless otherwise stated, reported values are model ensemble medians and ranges are model ensemble minimums and maximums. We follow the approach of a recent multimodel assessment in reporting model agreement in which ‘high’ agreement is judged at 66% or more of models agreeing in the direction of change^24^. Not all models report on all food systems dimensions (Supplementary Table 7), and we state where appropriate the number of models reporting a given result. An uncertainty and sensitivity assessment is given in Supplementary Information (Supplementary Figs. 4–6 and Supplementary Tables 14–17).

## Projected changes to global agriculture

**Fig. 1:**
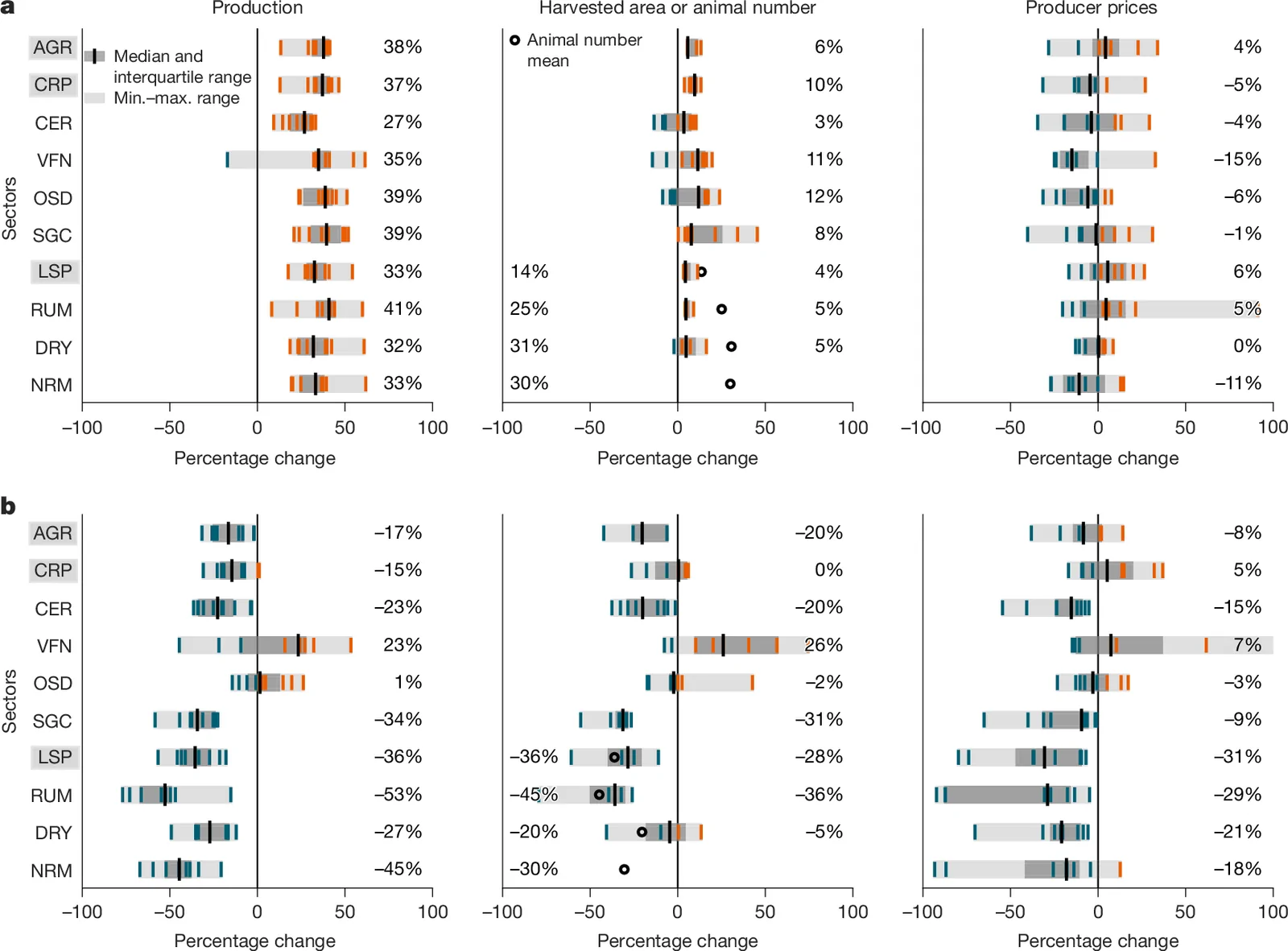
Global results from the model ensemble. **a**,**b**, Percentage change between a BAU scenario in 2050 versus current (2020) (**a**) and the EL2 scenario versus a BAU scenario in 2050 (**b**). Results are shown for sectors across three food systems dimensions—agricultural production, area (harvested area for crops, pasture area and animal numbers for livestock) and producer prices. Shaded areas give the range of results (dark grey, interquartile range; light grey, minimum (min.)–maximum (max.) range) with individual model results given by teal (percentage decrease) and orange (percentage increase) lines. Black vertical lines indicate median values which are also expressed numerically in each figure (on the right-hand side). Median percentage change in animal numbers is given by unfilled black markers and is represented numerically on the left-hand side. AGR, all agricultural products (but excluding fisheries and aquaculture); CRP, all crops, including cereals (CER) (wheat, rice and coarse grains), vegetables, fruits, nuts and legumes (VFN), oilseeds (OSD) and sugar crops (SGC) (all shown) and other minor food crops and non-food crops (energy crops and plant-based fibres; not shown separately); LSP, all livestock products, including ruminant meat (RUM) (cattle, sheep and goats), non-ruminants (NRM) (pork, poultry meat and eggs), dairy (DRY) and a category of other animal products (for example, wool; not shown separately). LSP excludes fisheries and aquaculture.

**Fig. 2:**
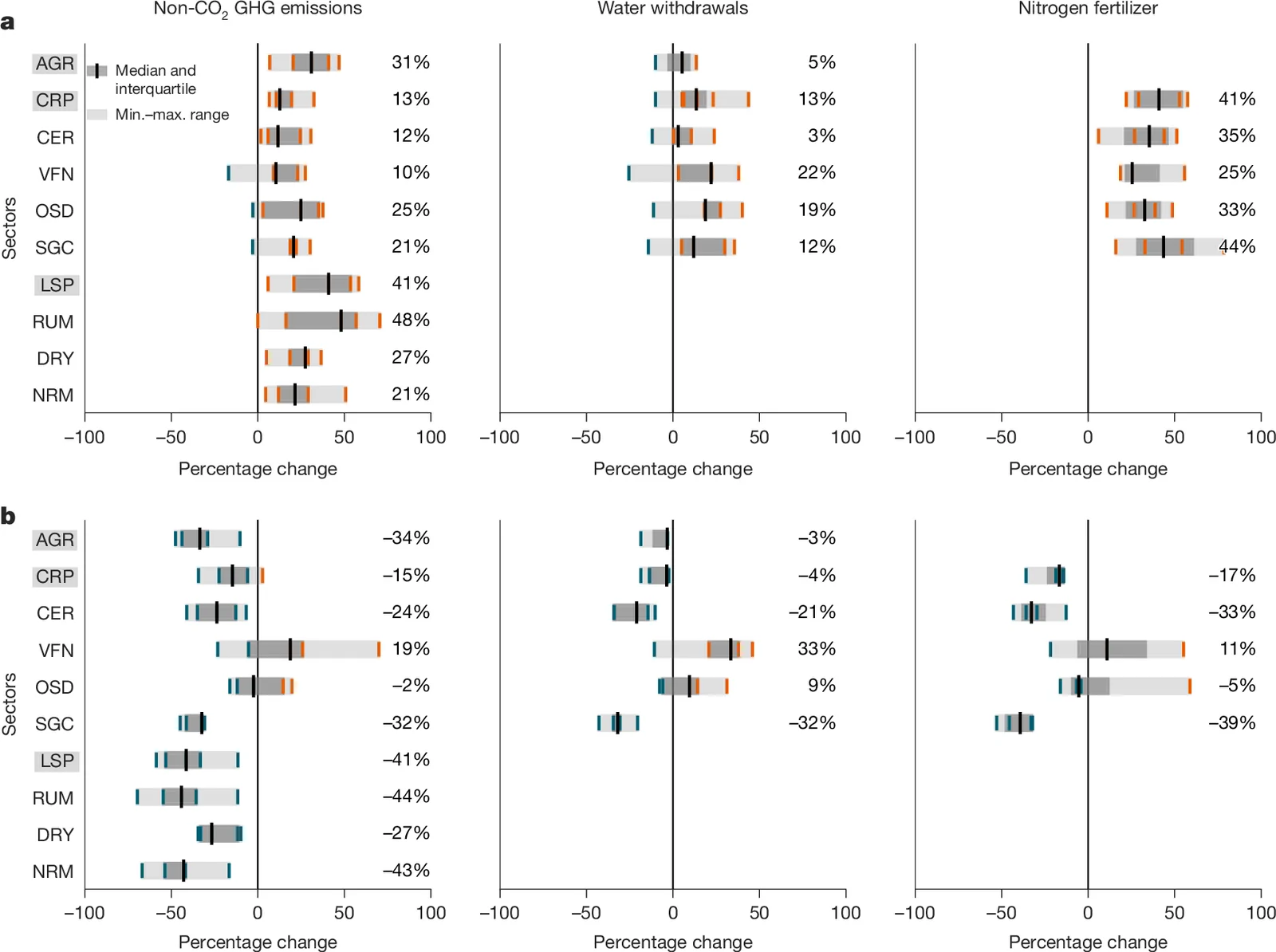
Global environmental results from the model ensemble. **a**,**b**, Percentage change between a BAU scenario in 2050 versus current (2020) (**a**) and the EL2 scenario versus a BAU scenario in 2050 (**b**). Results are shown for sectors across three environmental dimensions—non-CO_2_ GHG emissions, water withdrawals and nitrogen fertilizer use. Shaded areas give the range of results (dark grey, interquartile range; light grey, minimum–maximum range) with individual model results given by teal (percentage decrease) and orange (percentage increase) lines. Black vertical lines indicate median values which are also expressed numerically in each figure (on the right-hand side). LSP, RUM, DRY and NRM results for water withdrawal and nitrogen fertilizer are captured in crop sectors for feed production.

The BAU scenario shows a general increase across several key agriculture dimensions—production (inclusive of food, feed and other use), harvested area, animal numbers, real producer prices (Fig. 1a) and environmental impacts—non-CO_2_ GHG emissions, water withdrawals and nitrogen fertilizer use (Fig. 2a) by 2050 compared with 2020 levels. The EL2 scenario in general shows lower values relative to BAU by 2050, but with varying levels of agreement with respect to magnitude and direction of change across the ensemble (Figs. 1b and 2b). By 2050, there is high agreement (all models) that ruminant meat in the EL2 scenario is lower than the BAU scenario for production (−53%, nine models from −77% to −15%), animal numbers (−45%, three models from −70% to −33%) and producer prices (−29%, eight models from −92% to −5%). Dairy also shows high agreement, although with smaller magnitudes of change, for production (−27%, nine models from −49% to −12%), animal numbers (−20%, three models from −26% to −19%) and producer prices (−21%, eight models from −70% to −6%). Non-ruminant meat (and eggs) shows broadly comparable percentage changes to ruminant meat for production and animal numbers, and all but one reporting model projects lower producer prices relative to BAU.

**Extended Data Fig. 1:**
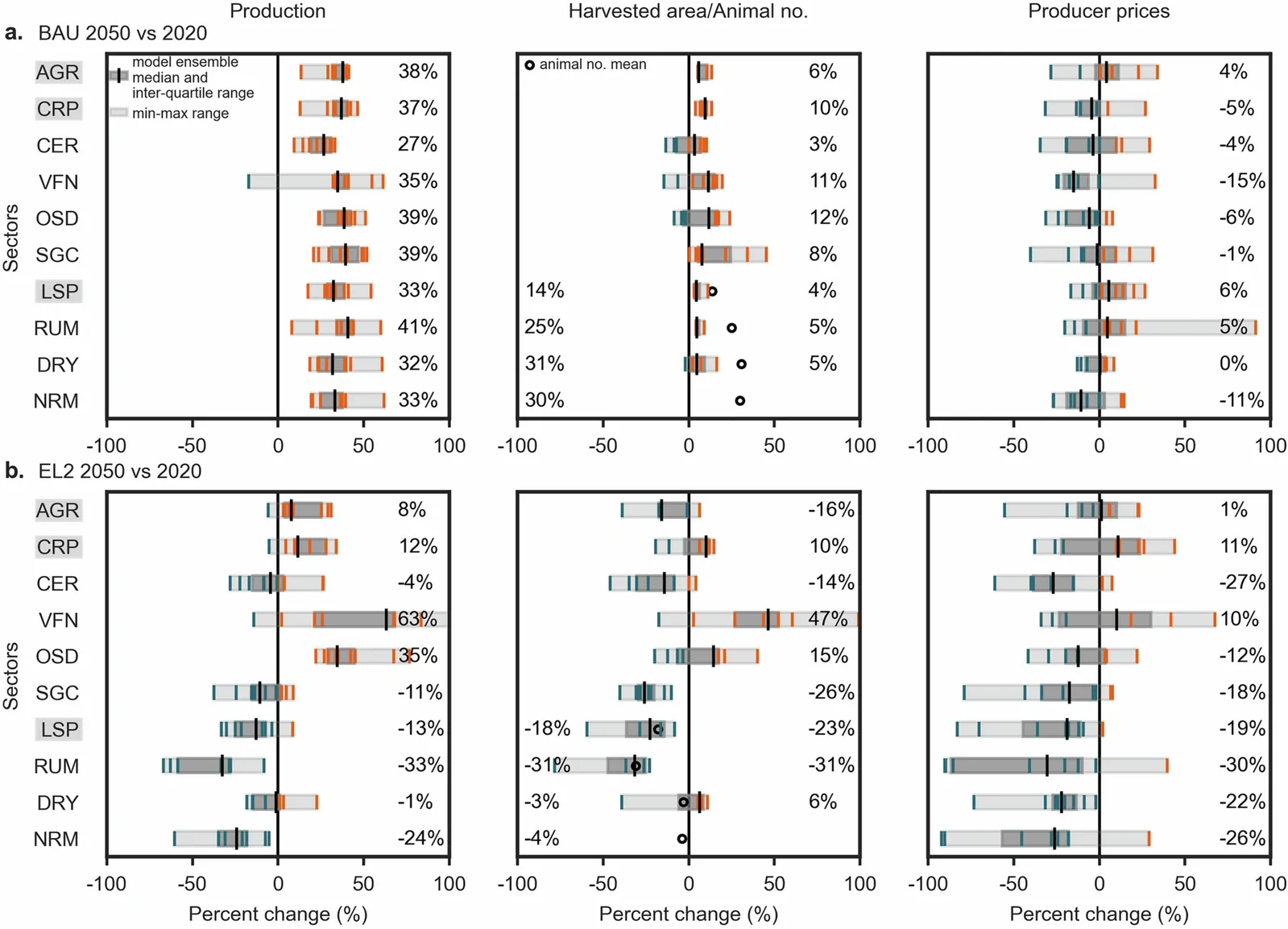
Global results from the model ensemble showing percentage change for 2050 scenarios versus current (2020). Percentage change between a BAU scenario in 2050 versus current (2020) (a) and the EL2 scenario in 2050 versus current (2020) (b). Results are shown for food group sectors across three food systems dimensions - agricultural production, area (harvested area for crops, pasture area and animal numbers for livestock), and producer prices. AGR = all agricultural products (but excluding fisheries and aquaculture), CRP = all crops including cereals (CER) (wheat, rice, coarse grains); vegetables, fruits, nuts, and legumes (VFN); oilseeds (OSD); and sugar crops (SGC) (all shown) and other minor food crops and non-food crops (energy crops and plant-based fibres; not shown separately), LSP = all livestock products include ruminant meat (RUM) (cattle, sheep, and goats), non-ruminants (NRM) (pork, poultry meat and eggs), dairy (DRY), and a category of other animal products (e.g. wool; not shown separately). LSP excludes fisheries and aquaculture. Shaded areas give the range of results (dark grey = inter-quartile range, light grey = min-max range) with individual model results given by teal (percentage decrease) and orange (percentage increase) lines. Black vertical lines indicate median values which are also expressed numerically in each figure (on the right-hand side).

Cereals also showed high agreement in the direction of change with lower values relative to BAU by 2050 across production (−23%, ten models from −36% to −3%), harvested area (−20%, nine models from −38% to −1%) and producer prices (−15%, nine models from −55% to −5%). Similarly, sugar crops showed high agreement (all models) in the direction of change in the EL2 scenario with lower values relative to BAU across production (−34%, ten models from −58% to −23%), harvested area (−31%, eight models from −55% to −27%) and producer prices (−9%, nine models from −65% to −1%). Conversely, the general trend for vegetables, fruits, nuts and legumes (VFN) was for larger values relative to BAU. Six of nine models showed increased production (+23%, nine models from −45% to +53%) and seven of nine models showed increased harvested area (+26%, nine models from −8% to +74%). VFN producer prices were more uncertain with a median of +7% change relative to BAU (seven models from −15% to +103%). Similarly, oilseeds showed mixed model agreement about the direction of change between EL2 and BAU scenarios. EL2 results versus 2020 levels are given in Extended Data Fig. 1, comparison with historic production quantities is given by Extended Data Fig. 3 and regional results on changes to production are given in Supplementary Fig. 7.

**Extended Data Fig. 2:**
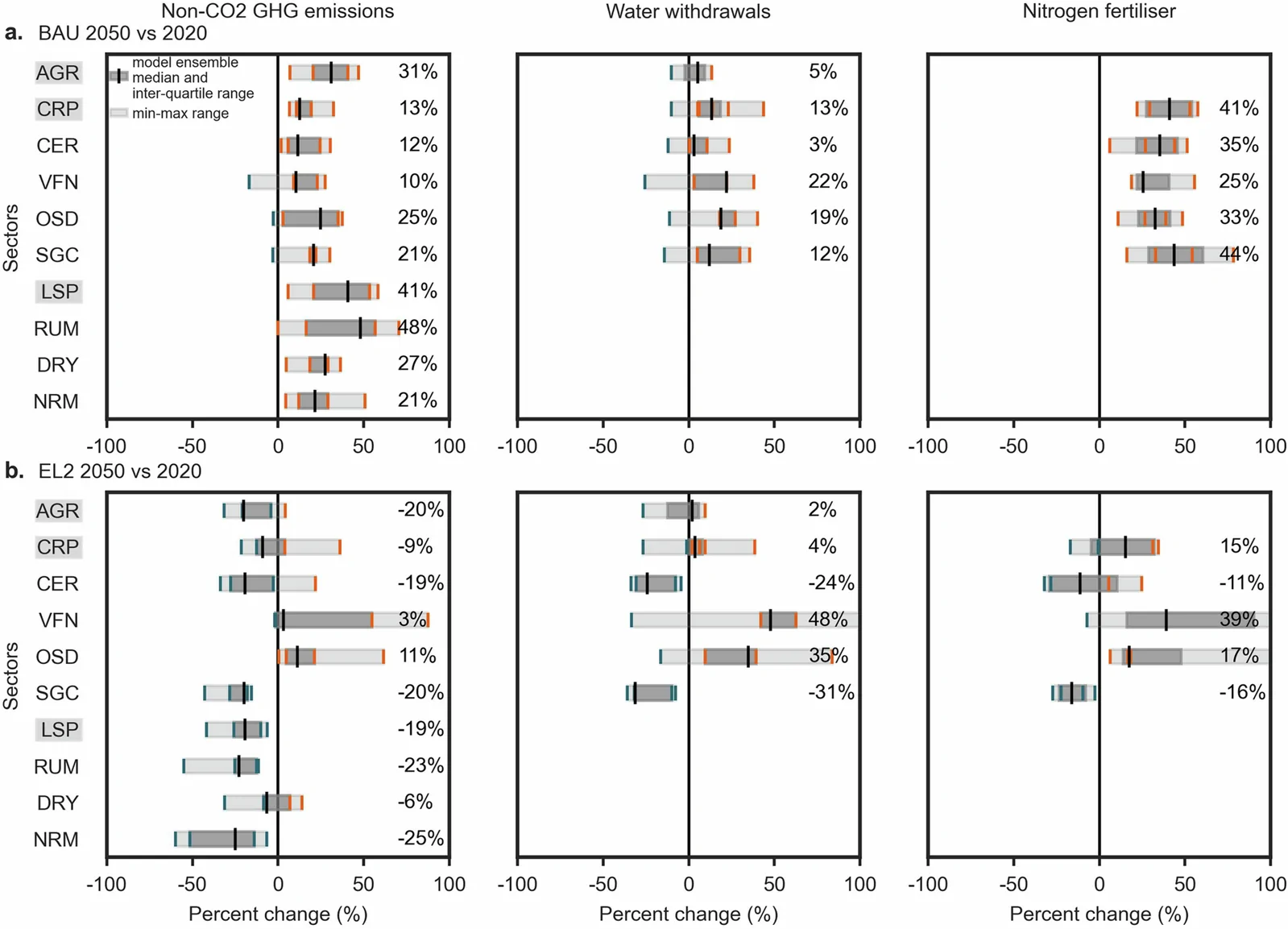
Global environmental results from the model ensemble showing percentage change between 2050 scenarios versus current (2020). Percentage change for a BAU scenario in 2050 versus current (2020) (a) and the EL2 scenario in 2050 versus current (2020) (b). Results are shown for sectors across three environmental dimensions – non-CO_2_ (GHG) emissions, water withdrawals, nitrogen fertiliser use. AGR = all agricultural products (but excluding fisheries and aquaculture), CRP = all crops including cereals (CER) (wheat, rice, coarse grains); vegetables, fruits, nuts, and legumes (VFN); oilseeds (OSD); and sugar crops (SGC) (all shown) and other minor food crops and non-food crops (energy crops and plant-based fibres; not shown separately), LSP = all livestock products include ruminant meat (RUM) (cattle, sheep, and goats), non-ruminants (NRM) (pork, poultry meat and eggs), dairy (DRY), and a category of other animal products (e.g. wool; not shown separately). LSP excludes fisheries and aquaculture. Shaded areas give the range of results (dark grey = inter-quartile range, light grey = min-max range) with individual model results given by teal (percentage decrease) and orange (percentage increase) lines. Black vertical lines indicate median values which are also expressed numerically in each figure (on the right-hand side). LSP, RUM, DRY, NRM results for water withdrawal and nitrogen fertiliser are captured in crop sectors for feed production.

**Extended Data Fig. 3:**
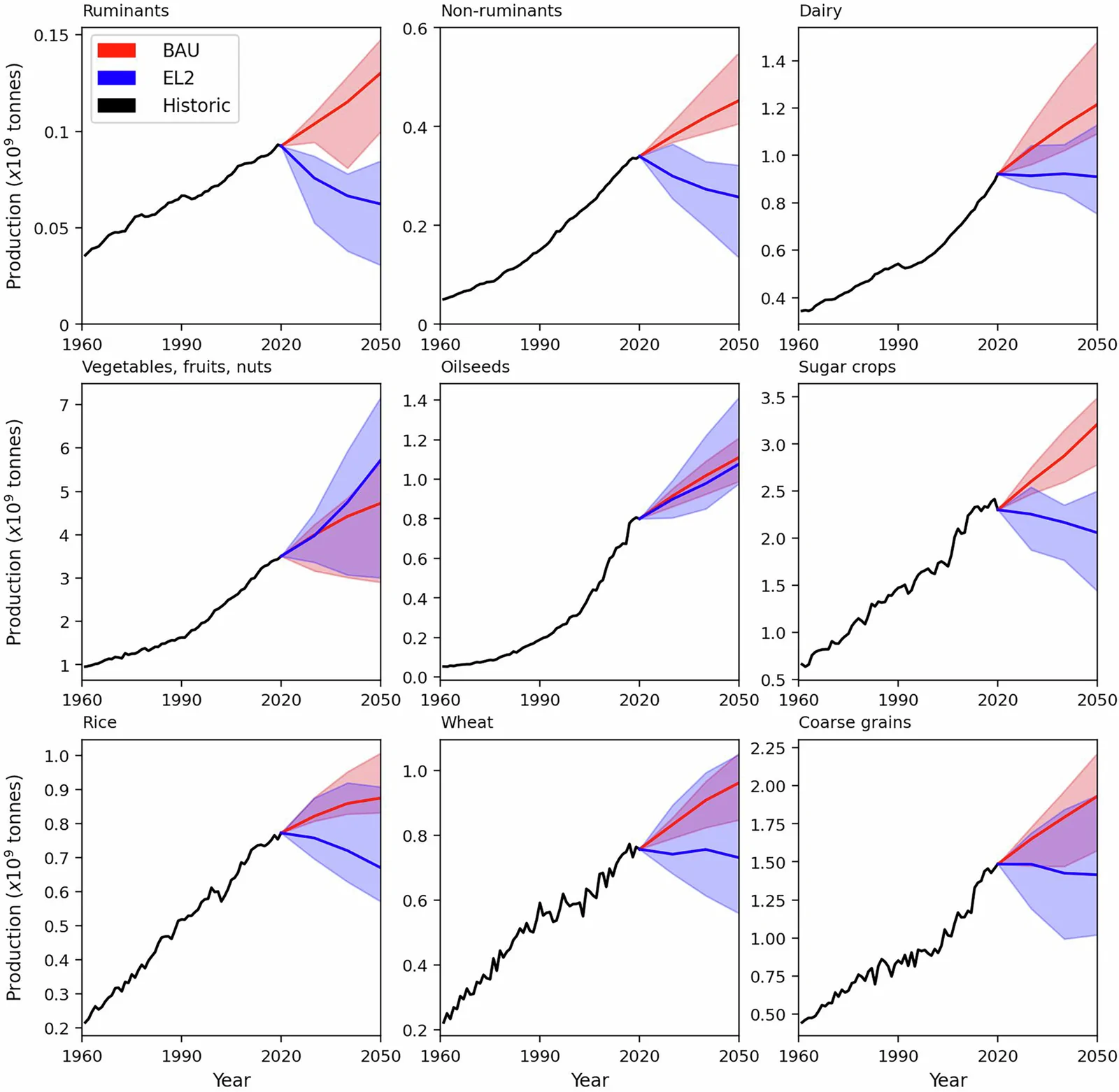
Changes in production (billion tonnes) from 1961 to 2020 (black) and then projections under BAU (red) and EL2 (blue) scenarios. Scenario shaded areas are bounded by upper and lower ensemble model results with the median result plotted as a solid line. Historic trends use FAO FAOSTAT data^68^.

Environmentally, direct non-CO_2_ GHG emissions from agricultural production (all agricultural products, but excluding fisheries and aquaculture) in the EL2 scenario are 20% (−1.2 GtCO_2_) smaller compared with 2020 levels (five models, −31% to 4%) (Extended Data Fig. 2 gives EL2 results compared with 2020 levels) and 34% smaller relative to BAU by 2050 (five models, −48% to −10%). Relative to BAU by 2050, water withdrawals are 3% smaller (three models, −19% to −3%) and nitrogen fertilizer uses for crops are 17% smaller (four models, −36% to −14%). Ruminant (five models, −70% to −12%) and non-ruminant meat (five models, −67% to −24%) GHG emissions are around 44% smaller relative to BAU by 2050. By contrast, GHG emissions from VFN are 19% larger relative to BAU, albeit under a wider range of model results (five models, −23% to 70%) (Fig. 2b). A subset of models (AIM, GLOBIOM, IMAGE and MAgPIE) also reported CO_2_ emissions and removals from land use change (not shown in Fig. 2). Agriculture-related net CO_2_ emissions from land use change in the EL2 scenario fell by 85% (−2.6 GtCO_2_) versus 2020 levels (four models, −117% to −66%) and are 76% lower relative to BAU by 2050 (four models, −113% to −67%).

## Restructuring of global agriculture

**Fig. 3:**
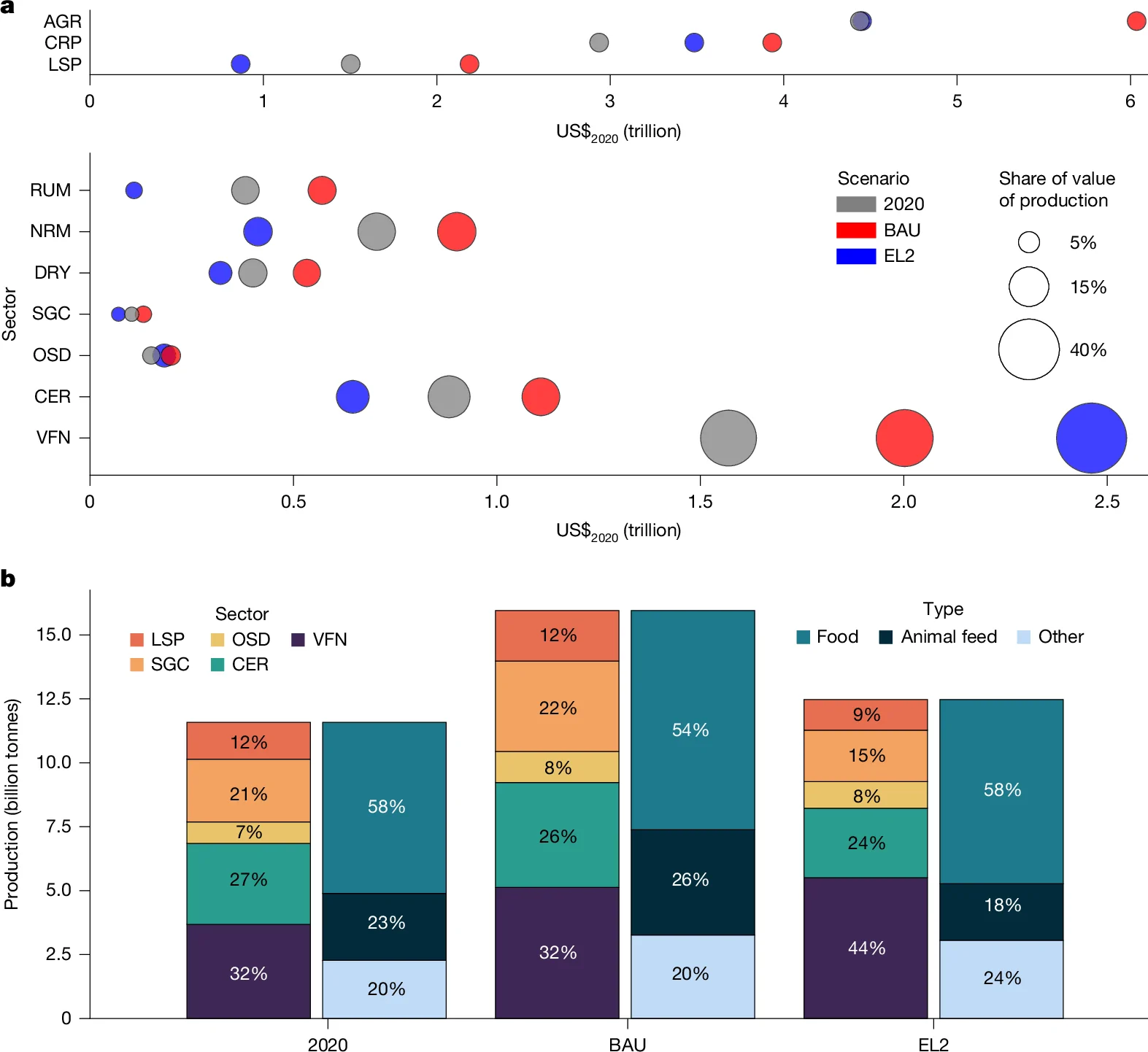
Production (economic value and physical) of different agricultural sectors for 2020 and for BAU and EL2 scenarios in 2050. **a**, The value of global agricultural production (trillion US$_2020_) and across different agricultural sectors. Median values of the model ensemble are given for BAU (red) and EL2 (blue) scenarios. Grey markers represent FAO value of production data for 2020, aggregated into sectors. The value of each of these sectors as a share of total value of production by scenario is given by the size of the markers. **b**, The composition of global agricultural production. Type of production is given as food, animal feed or other use (for example, industrial processes and bioeconomy).

By 2050 the EL2 scenario shows lower global agricultural production relative to BAU (high agreement, −17%) and a compositional restructuring of agricultural commodity sectors (Fig. 3b). Overall, the EL2 scenario shows a slightly higher share of agricultural production for food (+4 percentage points) and other uses (+4 percentage points) and a reduction in animal feed (−8 percentage points) compared with BAU by 2050. The use of traditional livestock feeds in EL2 highlights this shift, with oilseed production directed away from animal feed (−16 percentage points) towards direct human consumption and other uses (Supplementary Figs. 12 and 13).

Economically, all models show a smaller value of terrestrial agricultural production by 2050 in the EL2 compared with BAU scenario (−26%, from US$_2020_6 trillion per year in BAU to US$_2020_4.5 trillion per year in EL2) (Fig. 3a). Most sectors also see absolute decreases compared with 2020 values, but for the entire agricultural sector as a whole, EL2 and 2020 levels are comparable. The value of production of the global livestock sector (meat, dairy, eggs and other animal products) in EL2 is substantially lower than BAU by 2050 (Fig. 3a). This represents US$1.3 trillion lower production value compared with BAU by 2050 (US$870 billion versus US$2.2 trillion, −60%) and a US$630 billion decrease compared with 2020 (US$870 billion versus US$1.5 trillion, −42%). Ruminant meat contributes around 36% to this reduction, being US$473 billion lower (−81%, seven models from −96% to −61%) compared with BAU in 2050, as well as an absolute reduction on 2020 levels. This is smaller (in 2020 real terms) than at any point since 1961 (Extended Data Fig. 7).

Major commodity crops—sugar (−47%, eight models from −63% to −24%) and cereals (−42%, eight models from −58% to −15%—are around US$500 billion lower by 2050 compared with BAU (Fig. 3a). Some of this reduction in agricultural production value is offset by growth of VFN sectors (+23%, six models from −33% to +106%). Oilseeds show less agreement (five of eight models show a smaller value relative to BAU), given the role of oilseeds as inputs into livestock but also for biofuel and bioenergy production and plant-based foods (Supplementary Fig. 9 for changes in land for energy crops across scenarios). In terms of value share, the livestock sector shrinks from 36% (in BAU) to 20% of terrestrial agricultural output by value in the EL2 scenario, whereas VFN raises its share from 34% (in BAU) to 58% (in EL2).

Two-thirds of the difference in the overall value of agricultural production between BAU and EL2 is accounted for by changes in production, with the remainder explained by changes in prices. This is more pronounced for livestock with changes to production of ruminant meat contributing around 80% of the total change in value of production. These economic results show that a more sustainable food system supplying healthier diets could be achieved by 2050 with similar aggregate value of production to 2020 levels and substantially below those projected in the BAU scenario.

From a regional perspective there are substantial differences in changes to value of production (Extended Data Fig. 8). Sub-Saharan Africa (+60%) and India (+40%) both show large increases in production value in EL2 compared with 2020, whereas China (−42%) and Brazil (−35%) show a decrease on 2020 levels. These last two countries are the only ones that see a substantial decline in total plant-based value of production (−31% and −18%, respectively), possibly reflecting their position (along with the USA) as the top animal feed producers.

These results show a substantial restructuring of agriculture that has consequences for livelihoods and trade. Agricultural employment (reported by three models) showed similar decreases in both scenarios (median of −28%) by 2050 versus 2020. However, within this, employment in crop sectors in EL2 would be 17% larger than under BAU (although still lower than in 2020) whereas employment in livestock would be 36% lower than under BAU (Extended Data Table 2).

There is regional heterogeneity for changes to the share of imports of total consumption and share of exports of total production when comparing BAU and EL2. Europe and the USA, with populations on average consuming far in excess of EAT–*Lancet* diet red meat levels, see relative increases in ruminant exports as a share of production (from 21% to 34% and 4% to 9%, respectively) in the EL2 scenario versus BAU. South and Central America see a relative fall in export share of ruminant production from around 19% to around 10%. On the import side, Southeast and Other Asia (Supplementary Table 10 lists included countries) see a relative increase in the share of imports in total VFN consumption (from 11% to 17% and 17% to 25%, respectively) in EL2 versus BAU (Supplementary Fig. 10).

## Historical context

**Fig. 4:**
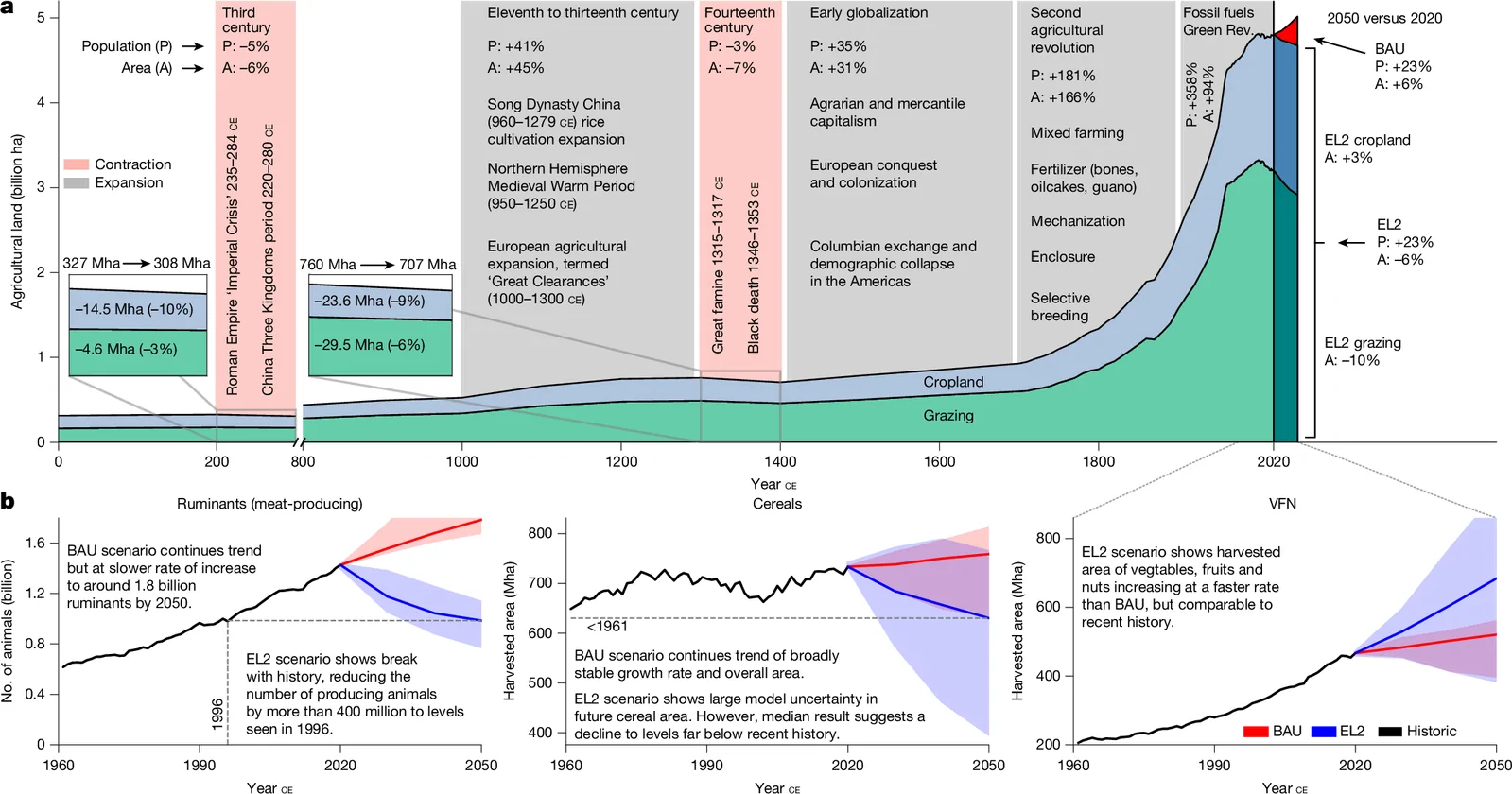
Long-run historical agricultural land use changes and trajectories from recent history. **a**,**b**, Comparison of long-run historical agricultural land use changes with a BAU and EL2 scenario (**a**) and comparison of trajectories from recent history with BAU and EL2 scenarios for animal numbers and harvested crop area (**b**). **a**, The development of global agricultural land (billion ha) from around 0 ce to 2020 coupled with possible changes under BAU (red shading) and EL2 scenario (darker blue and green) out to 2050. The green plotted area is grazing land and the blue plotted area is cropland. Time periods highlighted in pink (third century ce and fourteenth century ce) show instances of global reduction in agricultural land and time periods in grey highlight key instances of agricultural land expansion. Pink and grey periods are annotated with key influencing events and the percentage changes to agricultural area (given by ‘A’) and global population (given by ‘P’). BAU and EL2 scenario results show median model ensemble values for changes to grazing and cropland. **b**, Global animal numbers and harvested area from 1961 to 2020 (black lines) and the two scenarios (BAU given in red and EL2 given in blue) from 2020 to 2050. The red and blue lines show median values with the shaded areas showing minimum and maximum values from the model ensemble. Three sectors are given (ruminant meat, cereals and VFN), selected on the size of their contribution to global agricultural land use changes under EL2. Equivalent historic levels are indicated for median model values for an EL2 scenario in 2050 for ruminant meat animal numbers and cereals. Green Rev., Green Revolution.

**Extended Data Fig. 4:**
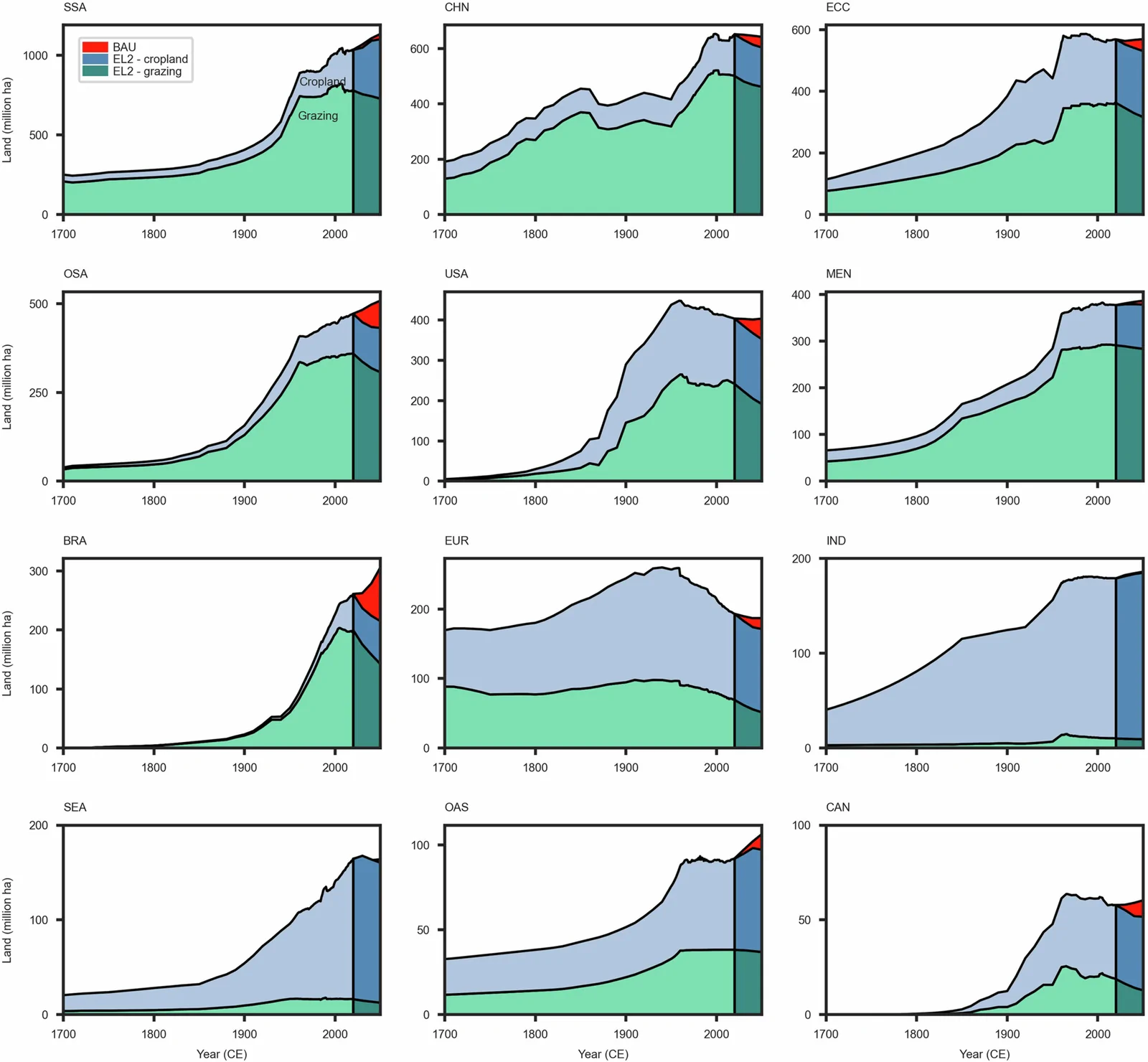
Comparison of long-run historical agricultural land use changes with a BAU and EL2 future scenario for different global regions. Supplementary Fig. 10 shows the development of agricultural land (million ha) from around 1700 CE to 2020 for 12 global regions coupled with possible changes under BAU (red shaded) and EL2 scenario (darker blue and green) out to 2050. The green plotted area is grazing land, and the blue plotted area is cropland. BAU and EL2 scenario results show median model ensemble values for changes to grazing and cropland. Note the different scale on the y-axis. Region codes are BRA = Brazil; CAN = Canada; CHN = China; EUR = Europe; ECC = Eastern Europe, Caucasus, Central Asia; IND = India; MEN = Middle East and North Africa; OAS = Other Asia; OSA = Other South and Central America; SEA = Southeast Asia; SSA = Sub-Saharan Africa; USA = United States of America. Historic source data from HYDE^69^.

A transformed future food system (EL2) could result in the largest absolute reduction (−274 Mha) in agricultural land in more than 2,000 years (Fig. 4a), whereas current trends (BAU) suggest a net increase by 2050 (6%, six models from 1% to 7%). Compared with 2020, all models showed grazing land for livestock falling in the EL2 scenario (−10%, six models from −30% to −4%). This reduction was partially offset by an increase in cropland (3%, six models from −19% to 11%). This amounts to a 6% reduction versus 2020 (six models from −26% to 1%), equivalent to late 1970s levels of agricultural land when the global population was less than 5 billion. On a compound annual growth rate basis, both scenarios showed substantial reductions in land expansion compared with historical trends and under the EL2 scenario these are even more pronounced than in the BAU scenario. Specifically, expansion in cropland under both scenarios (0.10% per year for EL2 and 0.28% per year for BAU) showed a slower rate than observed so far in the twenty-first century (0.33% per year on average since 2000). Grazing land under the EL2 scenario has a rate of −0.40% per year compared with a BAU rate of 0.05% per year. Although grazing land has recently declined, an EL2 scenario implies a rate of contraction 2.5 times faster than has been observed over 2000–2020 (−0.40% per year versus −0.16% per year) (Extended Data Table 1). Detailed results of global and regional land-use change (cropland, grazing land, forest and other natural vegetation) are given in Extended Data Figs. 4 and 6.

**Extended Data Fig. 5:**
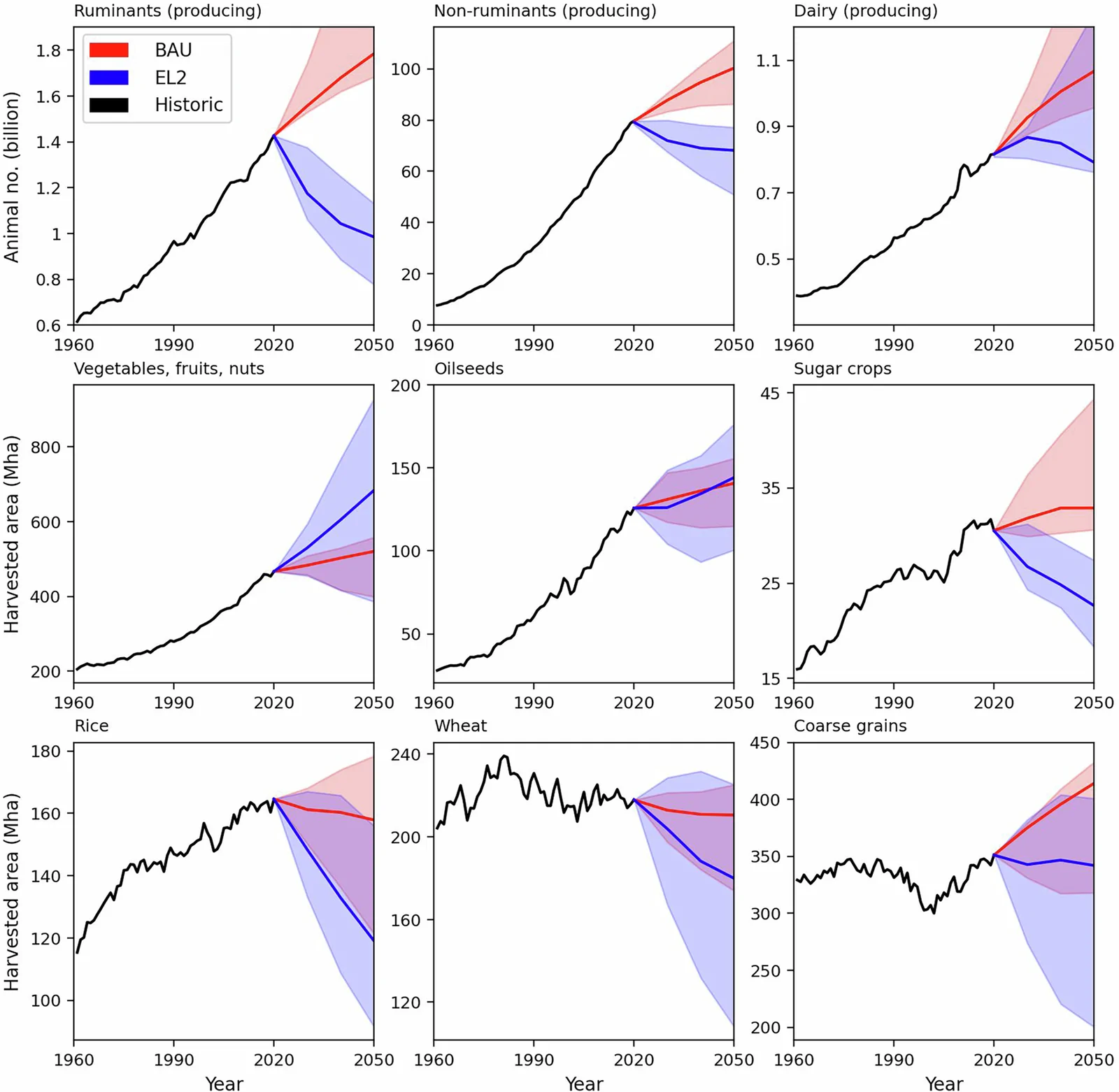
Changes in animal number (billion) and harvested area (Mha) from 1961 to 2020 (black) and then projections under BAU (red) and EL2 (blue) scenarios. Animal numbers refer to producing animals, not herds/stocks. Scenario shaded areas are bounded by upper and lower ensemble model results with the median result plotted as a solid line. Historic trends use FAO FAOSTAT data^68^.

Key drivers of crop and grazing land change in the EL2 scenario to 2050 are reductions in ruminant animal numbers (cattle, sheep and goats)—although this relationship is not a one-to-one reduction as not all ruminant production is in extensive grassland systems (Supplementary Fig. 8 and Supplementary Table 18 provide further analysis on changes to ruminant production and grassland). Ruminant animal numbers mark a distinct break with the past, corresponding to more than 400 million fewer producing animals than 2020 levels or 800 million fewer than a BAU scenario, down to levels last seen in 1996 (Fig. 4b). For other livestock, the EL2 scenario showed a 14% decline in non-ruminant animals (poultry and pigs) for slaughter (11 billion, mostly chicken) versus 2020 levels and 32% lower (32 billion) relative to the BAU scenario, equivalent to levels in 2014. Changes to dairy numbers corresponded to 25 million (−3%) fewer animals on 2020 levels or 275 million (−26%) fewer than the BAU scenario, equivalent to 2018 levels (Extended Data Fig. 5). Changes in cereal area under EL2 show uncertainty but trends downwards, versus recent stable harvest area which is continued in the BAU scenario. By contrast, the growth in VFN area in EL2 is consistent with decadal trends since the 1960s.

## Discussion

### Scale of the challenge

This study shows that several dimensions of a potential ‘great food transformation’^12^ break with history and in doing so highlights the scale of the challenge. Although grazing land may have already peaked in the last 20 years, the size of overall agricultural land reduction by 2050 suggested in EL2 is probably without precedent. The global reduction in economic value of livestock production and reduced meat consumption versus BAU projections would mark a paradigm shift in the role of farmed animals in many food systems (400 million fewer ruminant animals for meat, 42% decline in livestock production value on 2020 levels). And although the rate of increased production of healthy foods under a food systems transformation scenario is comparable to trends from the 1960s, this increase is higher relative to BAU projections and achieving it will necessitate increased investment. Historical comparisons highlight the scale of change and the challenge of achieving food systems transformation while managing many potential trade-offs.

However, the scale of change and challenges should be considered in the context of the substantial benefits that food system transformation offers. Global achievement of healthy diets for all would result in 15 million fewer adult deaths per year^25^. From a ‘true’ or ‘hidden’ cost perspective, current food systems have been estimated at US$10–20 trillion annually^26,27^, of which unhealthy dietary patterns account for most. Coupled with this, changes to land use (limited cropland expansion, 10% reduction in grazing land and 4% increase in forest cover versus 2020) offer sizeable climate change mitigation and biodiversity improvement potential^28^, in addition to the reduction in direct GHG emissions, largely from 400 million fewer ruminants. Specifically, agriculture-related net CO_2_ emissions from land use change in the EL2 scenario fell by 85% on 2020 levels.

To achieve larger environmental benefits, our results (consistent with previous studies^2,29^) show that more measures may be necessary. Modelling and scenario-based research could consider a more comprehensive conception of food systems transformation than explored here, for example incorporating sustainable and ecological intensification and circularity^30^ elements. Going further, more work is needed to develop, integrate and critically examine radical and fundamentally different scenarios of the future^31^ with greater stakeholder engagement and diversity of participation in the development of narratives of change and quantifiable scenarios. Research could also explore the interactions between food systems and wider social and economic transformations needed to tackle the climate and other crises. Finally, future research should consider questions of strategy and pathways for how to achieve food systems transformation, including national pathways and, linked to this, assessments of the cost of transformation (for example, ref. ^32^) and how it is distributed.

### Navigating the impacts of transformation

**Extended Data Fig. 6:**
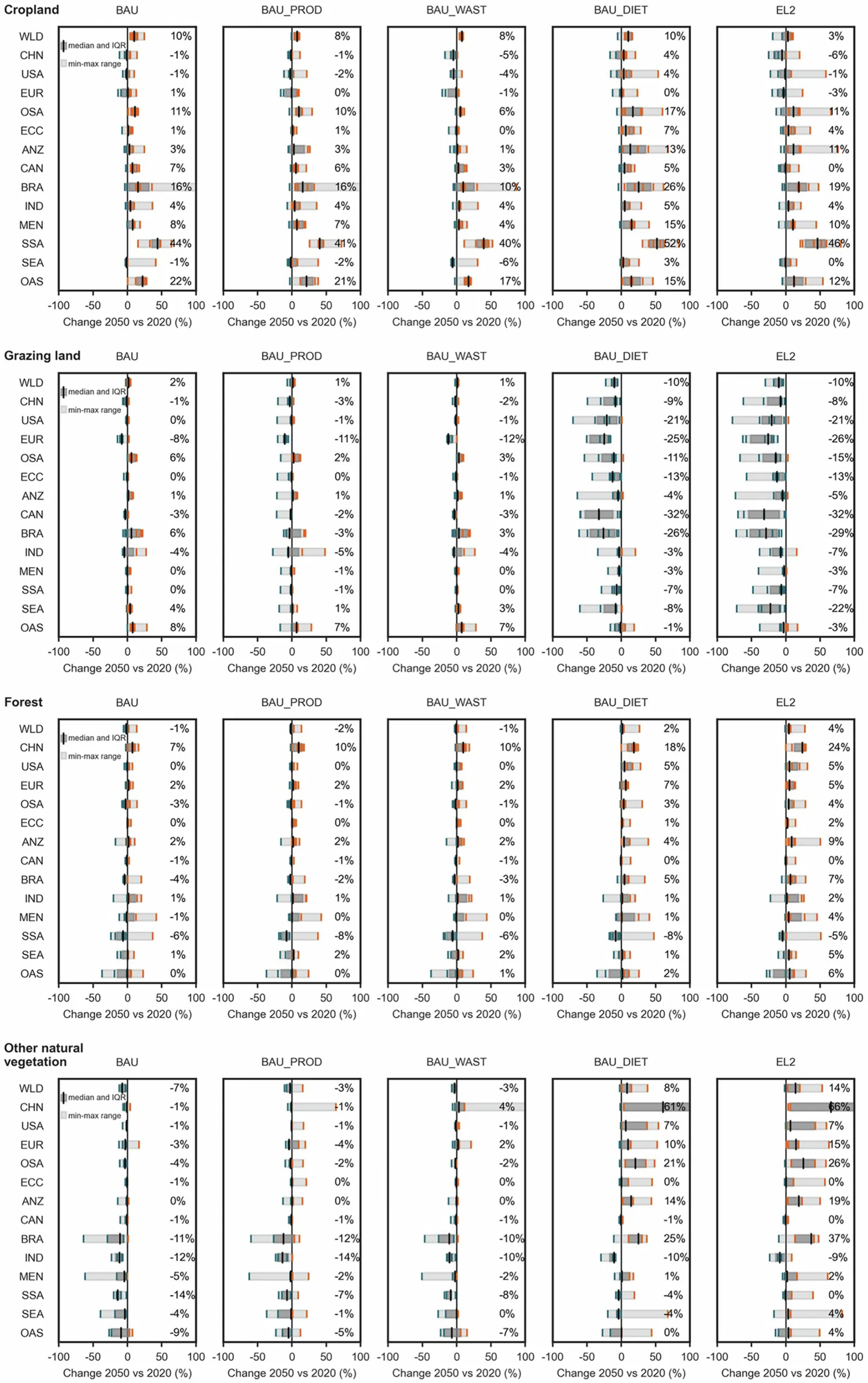
Changes to land-use types – cropland, grazing, land, forest, other natural vegetation for BAU, BAU_PROD, BAU_WAST, BAU_DIET and EL2 scenarios by 2050 versus 2020 levels. Results are given for the world (‘WLD’) and 13 global regions. Region codes are ANZ = Australia and New Zealand, BRA = Brazil; CAN = Canada; CHN = China; EUR = Europe; ECC = Eastern Europe, Caucasus, Central Asia; IND = India; MEN = Middle East and North Africa; OAS = Other Asia; OSA = Other South and Central America; SEA = Southeast Asia; SSA = Sub-Saharan Africa; USA = United States of America. Shaded areas give the range of results (dark grey = inter-quartile range, light grey = min-max range) with individual model results given by teal (percentage decrease) and orange (percentage increase) lines. Black vertical lines indicate median values which are also expressed numerically in each figure (on the right-hand side).

**Extended Data Fig. 7:**
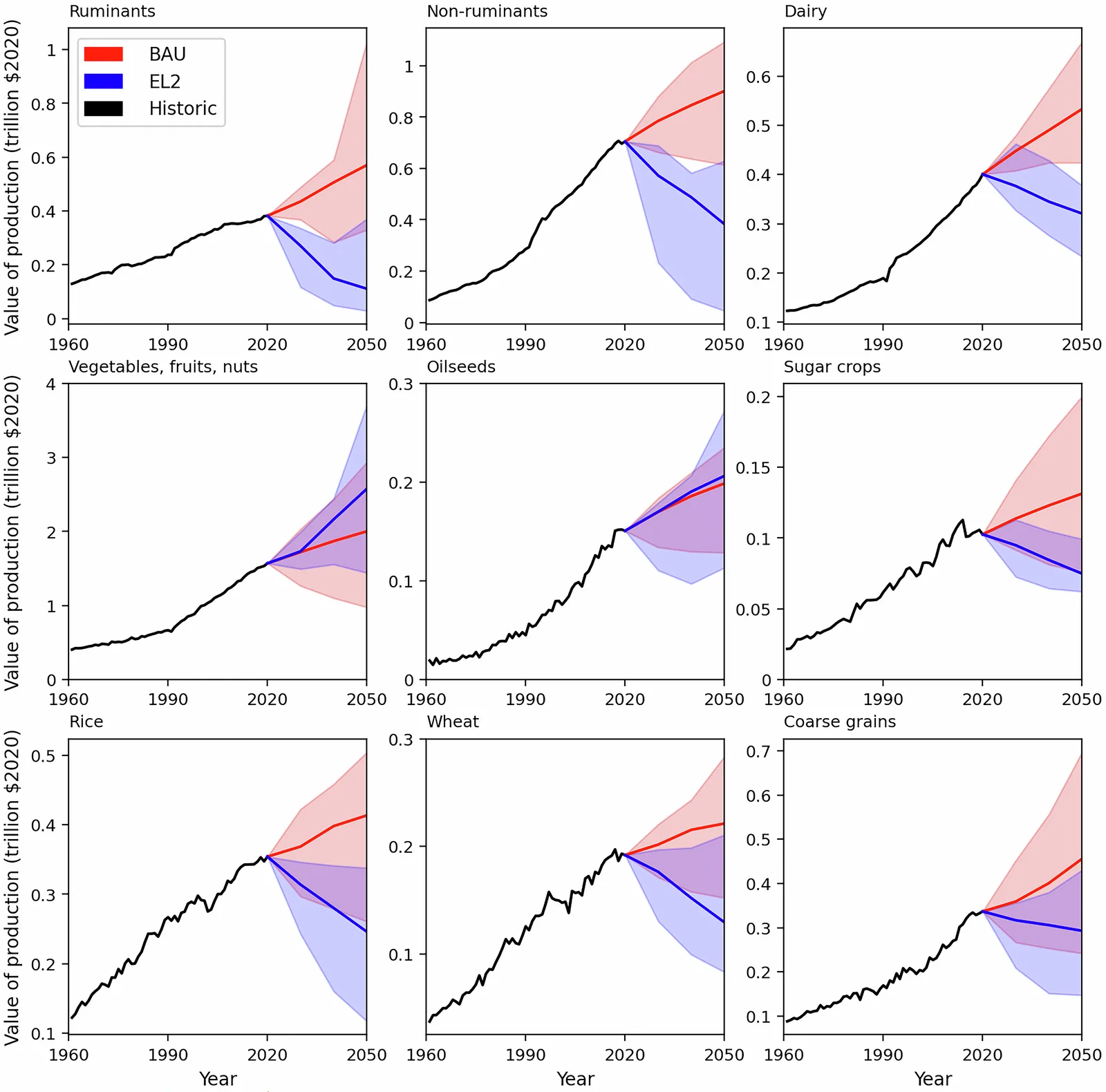
Changes in the value of production (trillion $2020) from 1961 to 2020 (black) and then projections under BAU (red) and EL2 (blue) scenarios. Scenario shaded areas are bounded by upper and lower ensemble model results with the median result plotted as a solid line. Historic trends use FAO FAOSTAT data^68^.

**Extended Data Fig. 8:**
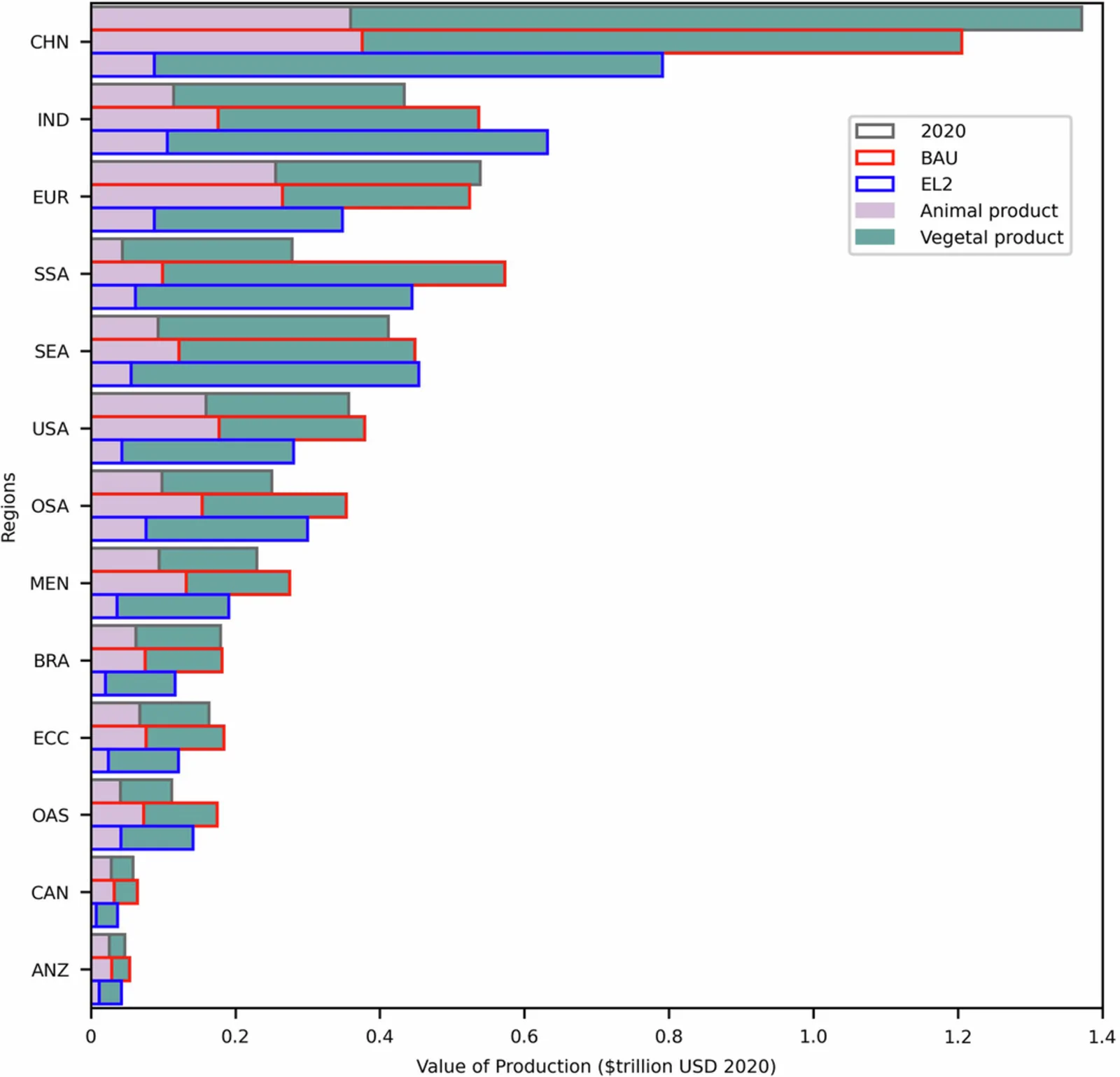
Total terrestrial value of agricultural production ($ trillion USD 2020) across 13 global regions. Results are given for 2020, BAU and EL2 scenarios in 2050. Bars are shaded to show the proportion of production value from animal (pink) and vegetal i.e. plant (green) products. Region codes are ANZ = Australia and New Zealand; BRA = Brazil; CAN = Canada; CHN = China; EUR = Europe; ECC = Eastern Europe, Caucasus, Central Asia; IND = India; MEN = Middle East and North Africa; OAS = Other Asia; OSA = Other South and Central America; SEA = Southeast Asia; SSA = Sub-Saharan Africa; USA = United States of America.

In a transformed food system scenario with a 10% reduction in grazing land (six models from −30% to −4%), land reallocation would occur from grazing to crops. Around 65%^33^ of grazing land is unsuitable for crop production and so, if not used for pasture or rangeland, would probably exit the agricultural sector altogether. This is context specific, but sizeable land-use change will probably be accompanied by trade-offs^34,35^. Countries have different and unequal biophysical endowments and climate change impacts that affect grass–arable conversion^36^ (Extended Data Figs. 4 and 6). A likely economic effect is downward price pressure on grazing land with limited immediate alternative use and market opportunity. This could result in land abandonment, as observed after the breakup of the Soviet Union in which cattle production declined precipitously^37^ and 30–60 Mha of agricultural land was abandoned^38–40^, leading to large carbon sinks which were subsequently reversed as the land was eventually recultivated. Future land use changes will occur in the context of existing unequal land distribution^41^, power asymmetries, unequal benefit capture and contested land tenure and rights^35^.

Linked with this, reductions in the value of production of ruminant and non-ruminant sectors would have serious ramifications for production practices and labour. It raises the possibility of accelerating consolidation (for example, as seen with dairy in the UK, USA and other markets) as small-to-mid-sized livestock producers could be priced out. This poses potentially sizeable consequences for livestock-oriented rural economies and livelihoods^42–45^.

### Consumers and commercial interest

Overall, the scale of change implied in a food system transformation requires commensurate ambition. As it stands, many policies and subsidies are working in opposition to food systems transformation^46^. Current levers and interventions aimed at changing aspects of food systems—for example, sugar taxes^47^, labelling^48^ and advertising restrictions^49^—are insufficient in isolation to deliver the magnitude of change required in the scenario studied here. The substantial relative reductions in production across livestock (36%), sugar (34%) and cereals (23%) by 2050 in the transformed food system versus a BAU scenario suggest profound implications for the political economy of global food systems. Powerful actors behind some commodity industries often move to resist, block or co-opt efforts^50,51^ and represent a principal impediment to food systems transformation^52,53^.

Another impediment is consumer behaviour, which in our scenario assumed a costless consumer preference shift towards healthy diets. In reality, there are challenges to changing consumer behaviour including questions of accessibility and affordability, as well as engaging with broader food cultures and individual tastes that all contribute to dietary choices^54^. Although the challenges are immense and raise serious questions over the feasibility of achieving such changes by 2050, the potential of substantial positive change should not be discounted, for example through social tipping points^55^ or health innovation (for example, GLP-1 (ref. ^56^)). This study does not explicitly contend with questions of consumer affordability, but some studies have assessed that a current EAT–*Lancet* diet could be more affordable for many in high-income countries but less affordable for many in lower-middle and low-income countries^57^. However, recent modelling suggests that an EAT–*Lancet* diet achieved by 2050 alongside reductions in FLW and projected per capita incomes rising faster than food prices, could be less expensive globally on average than BAU projected diets^58^ and, in a similar vein, we find that (producer) prices would be lower relative to BAU projections.

The global food systems transformation explored here fundamentally breaks with several historical trends (for example, on land use, and agricultural production and its value). Such a transformation would pose substantial political economy challenges with costs that are often concentrated (for example, the livestock sector) and benefits more diffuse (for example, general improvement of public health). Bold policy decisions in the present can shape and shield who wins and who loses in such a food systems future.

## Methods

### Multimodel ensemble approach

We simulated scenarios using a MME, building on the global economics group of AgMIP food system modelling efforts that have developed modelling protocols to explore future uncertainty and increase the comparability across various economic models^13,16,21,59^. The ensemble includes ten global economic models, which have featured in a wide range of high-level science policy reports (for example, ref. ^60^). The participating models are AIM, CAPRI, ENVISAGE, FARM, GCAM, GLOBIOM, IMAGE, IMPACT, MAGNET and MAgPIE. Although all models are global in scope, they have varying levels of detail in representing parts of global food systems, including food production, processing, trade and demand, as well as environmental and socioeconomic outcomes of these activities (Supplementary Tables 3 and 4 give a summary of participating models).

**Fig. 5:**
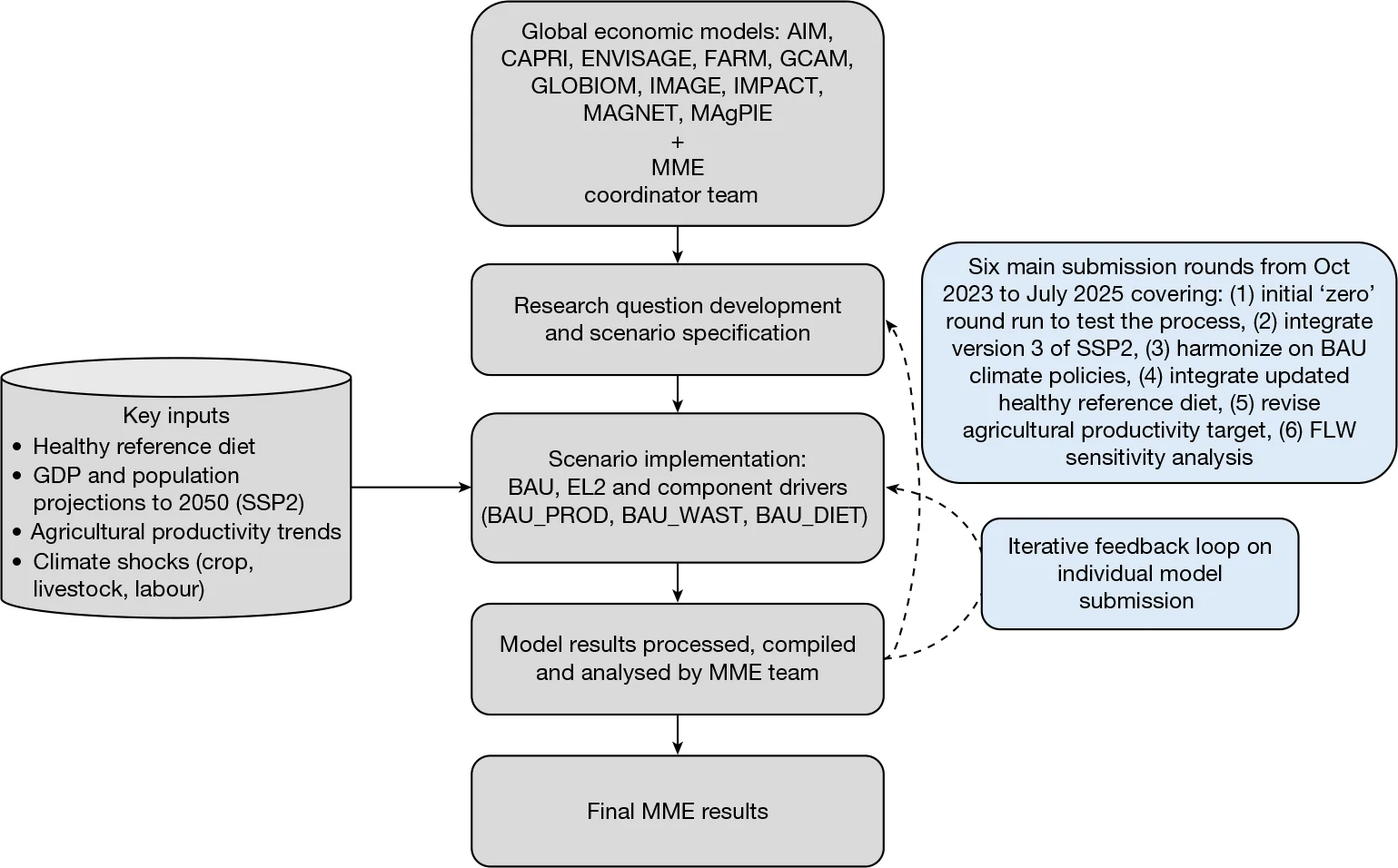
MME process undertaken for this study. Key data inputs and the main modelling results submission rounds are detailed.

Scenarios were developed by the MME coordinators and the individual model teams. The main scenario (EL2) was developed to mirror the core components in the 2025 EAT–*Lancet* report—shift to a healthy reference diet, increased agricultural productivity and a reduction in FLW, with the BAU scenario as a counterfactual to EL2. The scenario specification was shared with modelling teams to implement in their models. To align models on key inputs, data on the healthy reference diet, gross domestic product (GDP) and population projections from SSP2 v.3, productivity trends and climate shocks to crops, livestock and labour were shared with model teams. Model teams ran scenarios and shared results with the MME coordinators who processed and combined individual model scenario submissions. To ensure comparability, model outputs were converted to relative changes against 2020. There was a continual process of feedback and iteration during the exercise to harmonize on how models implemented scenarios and to compare preliminary results. The main forum for this was a monthly virtual meeting between all project participants supported by more frequent dedicated one-to-one engagement between the MME coordinators and individual model teams. In total, there were six main submission rounds—(1) initial ‘zero’ round run to test the process, (2) integrate v.3 of SSP2, (3) harmonize on BAU climate policies, (4) integrate updated healthy reference diet, (5) revise agricultural productivity target and (6) FLW sensitivity analysis—that were accompanied by individual model iterative submissions from modelling teams between October 2023 and July 2025. Figure 5 summarizes this process.

There were ten participating models in the study. Each has somewhat different structures in how they represent food, environmental, biophysical and economic systems. For detailed documentation on how each of these models captures these systems, behaviours and feedbacks, please follow the links in Supplementary Table 3 and see Supplementary Information for summaries.

### Scenarios and data inputs

Five scenarios and a further four sensitivity scenarios are presented here (Supplementary Table 1). Scenarios make use of the latest SSP2 data^5^ for population and GDP projections. We opt for SSP2 as it represents a ‘middle of the road’ pathway (see Supplementary Fig. 1 and Supplementary Table 2 for further details on SSPs). The first is a BAU scenario that reflects current trends based on SSP2 projections for key scenario drivers (GDP and population) and assumptions related to diets, crop and livestock productivity and FLW. The food systems transformation scenario (EL2) also uses SSP2 GDP and population projections but includes the EAT–*Lancet* reference diet, along with higher productivity growth rates similar to trends associated with SSP1 (SSP1 represents a more optimistic ‘sustainability’ path) and a reduction of FLW broadly consistent with the 50% reduction contained in Sustainable Development Goal 12.3. The final three scenarios represent individual drivers of this food systems transformation (shifts to the EAT–*Lancet* diet (BAU_DIET), improved productivity (BAU_PROD), and reduced food loss and waste (BAU_WAST)). Four sensitivity scenarios explore FLW reduction of 25% and 75% in the BAU and EL2 core scenarios, respectively.

The diet implementation requires data on food consumption categorized by food group and region for both the BAU and EL2 diet, provided by refs. ^2,18^. Supplementary Table 8 shows the global average reference intake values to be achieved by 2050. Models assume that populations make changes to the reference diet by way of a consumer preference shift. We assume that EAT–*Lancet* healthy reference diets will be achieved in all regions by 2050. Caloric coefficients (cal g^−1^) are held constant in the projection period. For food groups such as fruits and vegetables the intake value (g per day) was interpreted as a floor and populations in the models were free to consume more than this quantity. By contrast, many of the animal-sourced food groups were interpreted as a ceiling in which populations above this converged down to the target, but populations below this continued to follow country or region dietary trends up to and including this value.

The approach to the agricultural productivity scenario component was as follows. Future trajectories of potential growth in agricultural sector productivity have been developed by researchers at the International Food Policy Research Institute (which hosts the IMPACT model) over the last two decades^61^. Yield growth rate assumptions in IMPACT are periodically updated through consultation with experts (for example, Consultative Group for International Agricultural Research (CGIAR) centres), economic model comparison projects, trends in agricultural research expenditures and updates on trends in long term yield growth rates based on FAOSTAT data^62^. Yield trends from this process formed the BAU productivity growth rates. The higher rates in EL2 were constructed by scaling BAU rates by the difference in per capita GDP between SSP1 and SSP2 (see refs. ^63,64^ for details). On average, this gave a 10–15% percentage point increase in yield between BAU and EL2 over 2020–2050 (Supplementary Table 9). Yields in EL2 and BAU versus historic trends are given in Supplementary Fig. 11.

Scenarios did not include any more mitigation policies that have not already been implemented. Some specific climate-related impacts are included in line with representative concentration pathway (RCP) 7.0. We opt for RCP 7.0 as it broadly reflects the outcome of no further climate policy, representing an upper range of future GHG emissions and warming by 2050. These include climate-related impact shocks to crops (using IPSL GCM average of the global gridded crop model intercomparison (GGCMI) crop model ensemble for soybean, maize, wheat and rice^65^), livestock^66^ and agricultural labour^67^. The four main crops represented by the global gridded crop models (maize, wheat, rice and soybeans) were mapped to 36 crop commodities of IMPACT as closely as possible to their native crop representation. Likewise, the livestock climate shocks for meat and dairy were mapped to beef, lamb, pork, poultry, eggs and milk, respectively. Agricultural labour productivity trends are based on ref. ^67^ and reflect global reductions in manual agricultural work capacity owing to climate change. Supplementary Table 5 details how individual models implemented the scenario components.

### Reporting model outputs

As each model has different native reporting (across food sectors, variables, regions, units and time steps) we asked models to adhere to a reporting template to obtain a common and consistent set of model results. This study included 13 main sector types across crop and livestock products (Supplementary Table 6) and focused on global results (models reported globally and in 13 macro-regions; Supplementary Table 10). The main reported variables for each of the sectors were: producer prices, production, harvested area (crops), animal numbers (livestock), food use, feed use, other use, agricultural employment, GHG emissions, water withdrawals and nitrogen fertilizer use. Physical land was reported for cropland, grazing land and total agricultural land. Not all models report on all sectors and variables as a result of differing model structure, representation and specialization. Supplementary Table 7 shows the number of models reporting results for each food sector and variable combination. Results in this study were from model submissions up to July 2025.

### Historic (1961–2020) food systems data

FAO data^68^ were used to compare global production, area, animal numbers, yield and value of production from 1961 to 2020 with scenario results under BAU and EL2 up to 2050. FAO food items were aggregated into broader food sector categories (for example, ruminants or VFN). For consistency and effective comparison, the relative (percentage) change for modelled scenarios in 2050 versus 2020 values of models were applied to FAO mean values for 2019–2021. FAO GDP deflators were applied to the value of production data (natively given as 2014–2016 average) to calculate 2020 values. To calculate modelled value of production across scenarios, we take model reported producer prices and production by sector and region and apply these percentage changes (BAU scenario in 2050 versus 2020 reference year, and EL2 scenario in 2050 versus 2020 reference year or versus BAU in 2050) to FAO producer price and production data. We then estimate modelled value of production as the product of producer price and production.

### Long-run land use data

We obtain long-run historic agricultural land use and population from the history database of the global environment (HYDE) (v.3.3)^69^. HYDE gives data at global and country level from −10,000 ce to present.

### Reporting summary

Further information on research design is available in the Nature Portfolio Reporting Summary linked to this article.

## Online content

Any methods, additional references, Nature Portfolio reporting summaries, source data, extended data, supplementary information, acknowledgements, peer review information; details of author contributions and competing interests; and statements of data and code availability are available at https://doi.org/10.1038/s41586-026-10775-2.

## Supplementary information

## Figures and Tables

**Extended Data Table 1:**
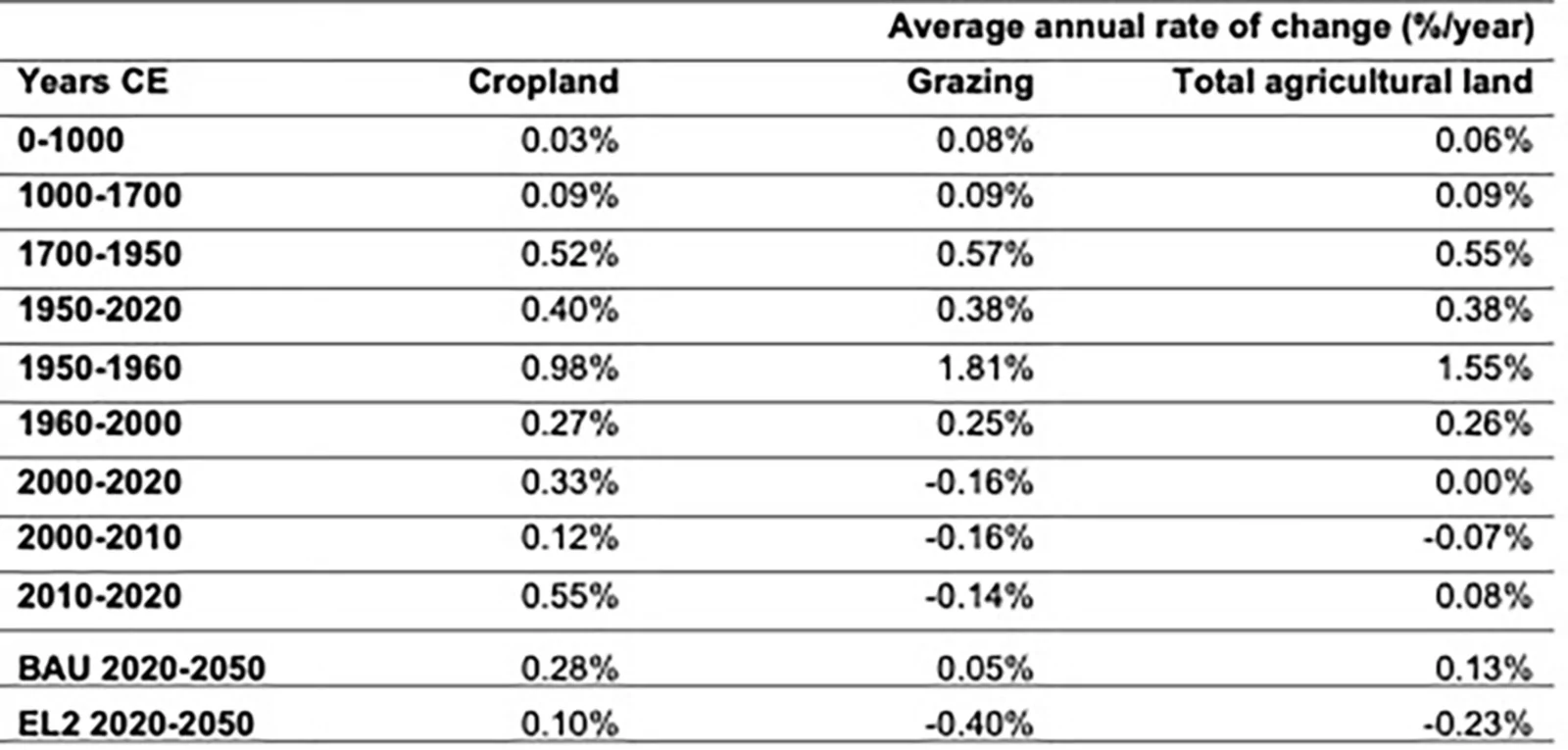
Average annual rate of change of global agricultural land across different historical periods with BAU and EL2 future scenarios for comparison

Historic source data from HYDE68.

**Extended Data Table 2:**
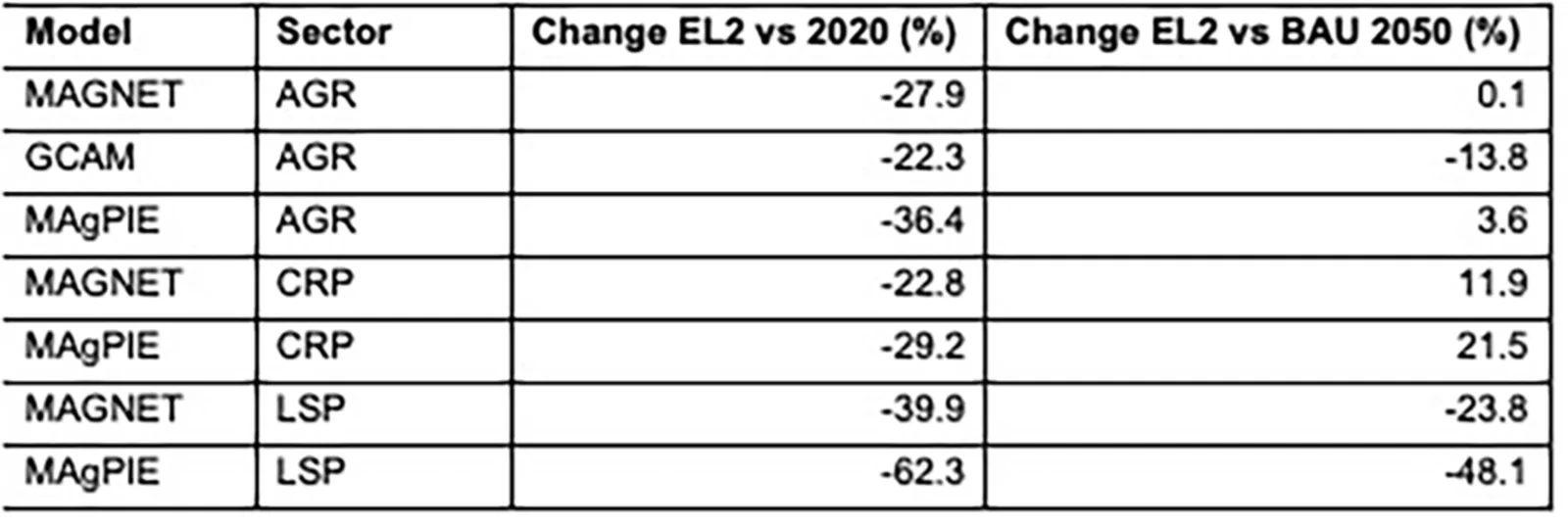
Relative change to agricultural employment in the EL2 scenario with 2020 and BAU in 2050

AGR = all agricultural products (but excluding fisheries and aquaculture), CRP = all crop products, LSP = all livestock products excluding fisheries and aquaculture.

## Data Availability

Data underlying model results that support the findings of this study are available at Zenodo (https://doi.org/10.5281/zenodo.17570720)^70^. FAO data can be found at https://www.fao.org/faostat/en/#data. HYDE long-run land-use data can be found at https://landuse.sites.uu.nl/datasets/.

## Code availability

We performed the analysis using Python. The scripts to replicate Figs. 1–4 are available at Zenodo (https://doi.org/10.5281/zenodo.14876855)^71^.
