# Supplementary material for: Food systems transformation would reshape global agriculture
Source: Nature. 2026 Jul 15;657(8130):156–63. doi: 10.1038/s41586-026-10775-2 (PMC13538146; doi:10.1038/s41586-026-10775-2)
Supplement: Supplementary file 1 [file 41586_2026_10775_MOESM1_ESM.pdf]

nature portfolio
https://doi.org/10.1038/s41586-026-10775-2

Supplementary information

Food systems transformation would reshape global agriculture

In the format provided by the authors and unedited

Supplementary Information

Table of Contents

Supplementary Information ***1***

Supplementary Methods **1**

Model summary 4

Model implementation of the main scenario components 13

Protocol reporting 24

Supplementary Discussion **28**

Literature review summary 28

Sensitivity and uncertainty assessment 35

Supplementary results 52

References **65**

Supplementary Methods

Scenario summary

Supplementary Table 1 summaries the five scenarios (BAU, BAU_DIET, BAU_PROD, BAU_WAST, and EL2) modelled by the ensemble. The table gives a description of each scenario and the core assumptions across the three modelled food system change components (diet shift, improved productivity, reduced food loss and waste).

Supplementary Table 1: Description and main assumptions of the scenarios modelled in the study*. NPi = National policies implemented.*

Scenario	Description	Climate change impact	Food system component		
			**Diet**	**Productivity**	**Waste**
BAU	Business as usual scenario based on SSP2 with a higher climate impact reflected by RCP 7.0	Crop model: Using IPSL GCM average of GGCMI crop model ensemble (RCP 7.0) for soybean, maize, wheat, and rice.<br><br>Livestock: Livestock productivity shocks (RCP 7.0) from ref [1] based on changes in higher temperatures.	Model BAU assumptions on diets to 2050	Model BAU assumptions on crop and livestock productivity.	Model BAU assumptions on FLW
BAU_DIET	Decomposition scenario. Adds EAT-Lancet diet to BAU		Globally achieves EL2 diet by 2050	Model BAU assumptions on crop and livestock productivity.	Model BAU assumptions on FLW
BAU_PROD	Decomposition scenario adds high productivity to BAU		Model BAU assumptions on diets to 2050	High productivity growth rate consistent with SSP1 productivity trends	Model BAU assumptions on FLW

BAU_WAST	Decomposition scenario. Adds a reduction (halving) of food loss and waste (FLW)	Labour: Labour productivity shocks (RCP 7.0) for agricultural labor from ref [2]	Model BAU assumptions on diets to 2050	Model BAU assumptions on crop and livestock productivity.	Reduction of FLW
EL2	Full EAT-Lancet scenario (diet, productivity, FLW) without mitigation and higher climate impacts based on RCP 7.0		Globally achieves EL2 diet by 2050	High productivity growth rate consistent with SSP1 productivity trends	Reduction of FLW

Supplementary Figure 1 and Supplementary Table 2 summarise socioeconomic input data from Shared Socioeconomic Pathways (SSPs) (v3.0) projections used in the modelling. Supplementary Figure 1 shows the GDP and population projections across the five SSPs. This study used SSP2 ('middle-of-the-road') projections on GDP and

population. Supplementary Table 2 gives GDP, population and GDP per capita projections for the 13 global regions (plus ‘world’) reported on in the study for 2050.

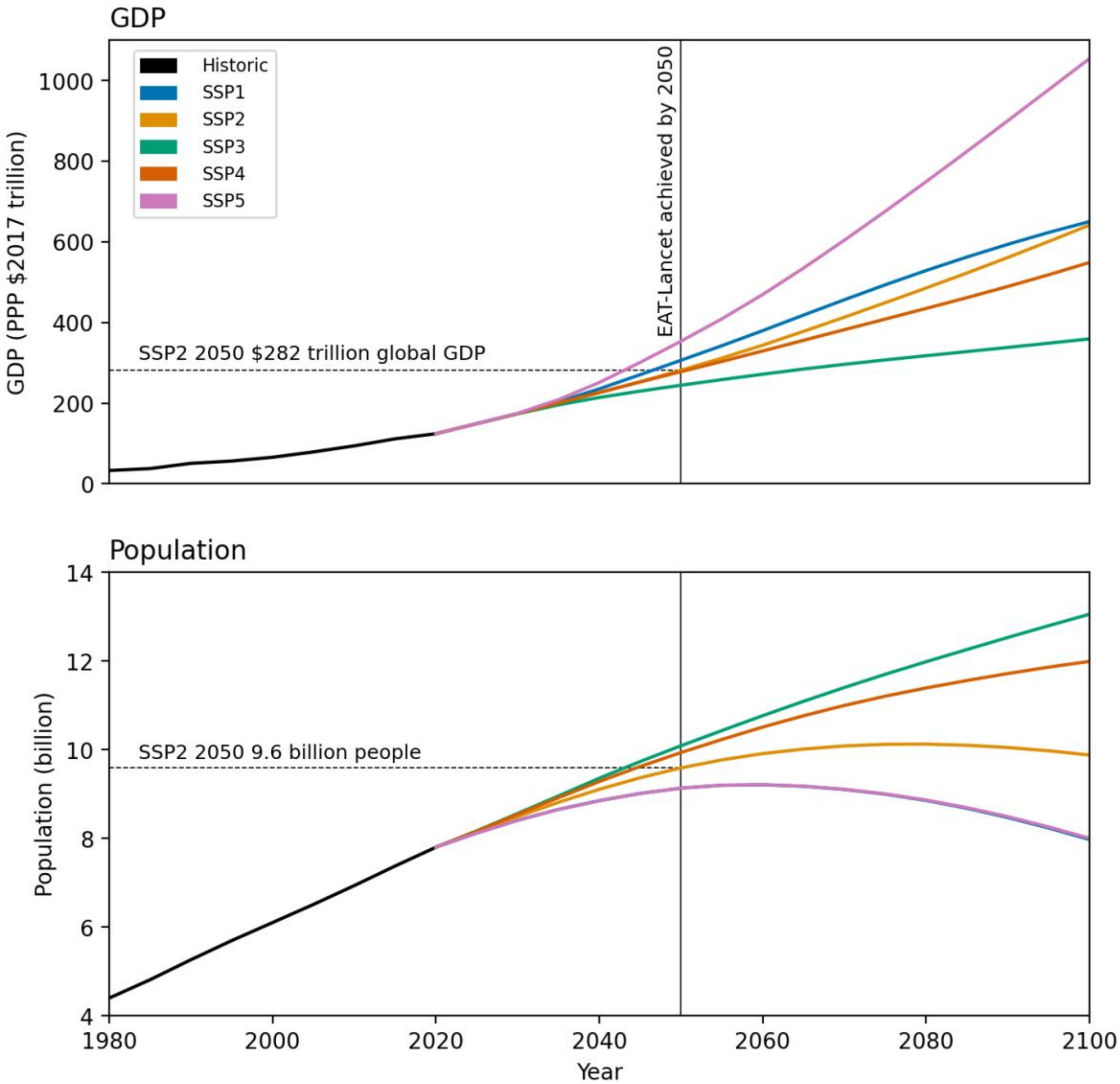

Supplementary Figure 1: Global GDP (PPP $2017 trillion) and population (billion) projections of five Shared Socioeconomic Pathways (SSPs). *Historic GDP and population are given by the black line. SSP1 = blue, SSP2 = gold, SSP3 = green, SSP4 = orange, SSP5 = pink. Vertical lines indicate the scenario end points at 2050. The dashed horizontal lines indicate SSP2 GDP and population in 2050.*

Supplementary Table 2: GDP, population and inferred GDP per capita projections under SSP2 for the world (WLD) and 13 global regions. *Data is given for 2020, 2050, and the percentage change between the two. Region codes are ANZ = Australia and New Zealand; BRA = Brazil; CAN = Canada; CHN = China; EUR = Europe; ECC = Eastern Europe, Caucasus, Central Asia; IND = India; MEN = Middle East and North Africa; OAS = Other Asia; OSA*

= Other South and Central America; SEA = Southeast Asia; SSA = Sub-Saharan Africa; USA = United States of America.

SSP2	GDP (billion USD2017/year)			Population (million)			GDP per capita (USD2017/year)		
**Region**	**2020**	**2050**	**Change (%)**	**2020**	**2050**	**Change (%)**	**2020**	**2050**	**Change (%)**
**WLD**	124,204	281,700	127	7,805	9,594	23	15,913	29,364	85
**ANZ**	1,399	2,865	105	31	37	21	45,759	77,761	70
**BRA**	2,936	6,623	126	213	228	7	13,811	29,073	111
**CAN**	1,650	3,088	87	38	42	12	43,696	73,237	68
**CHN**	23,972	55,133	130	1,432	1,314	-8	16,738	41,950	151
**EUR**	21,437	35,141	64	543	540	-1	39,478	65,113	65
**ECC**	5,489	10,075	84	290	301	4	18,897	33,438	77
**IND**	8,504	34,136	301	1,390	1,620	17	6,118	21,067	244
**MEN**	9,758	26,529	172	555	762	37	17,592	34,808	98
**OAS**	2,652	10,643	301	499	730	46	5,314	14,580	174
**OSA**	5,840	14,257	144	436	515	18	13,397	27,660	106
**SEA**	16,578	34,435	108	868	950	9	19,091	36,251	90
**SSA**	4,202	16,404	290	1,127	2,123	88	3,729	7,728	107
**USA**	19,691	32,001	63	335	377	12	58,710	84,877	45

Model summary

Supplementary Table 3 gives the 10 participating models in the study. Links are provided to detailed model documentation.

Supplementary Table 3: *Participating models in the study. IAM = integrated assessment model, CGE = computational general equilibrium, PE = partial equilibrium.*

Model	Model type	Institutions	Documentation
AIM	IAM/CGE	National Institute for Environmental Studies & Kyoto University	AIM
CAPRI	PE	European Commission Joint Research Centre	CAPRI

ENVISAGE	CGE	Purdue University	ENVISAGE
FARM	CGE	U.S. Department of Agriculture	FARM
GCAM	IAM/PE	Pacific Northwest National Laboratory, Joint Global Change Research Institute (PNNL, JGCRI)	GCAM
GLOBIOM	PE	International Institute for Applied Systems Analysis (IIASA)	GLOBIOM
IMAGE	IAM	Netherlands Environmental Assessment Agency (PBL)	IMAGE
IMPACT	PE	International Food Policy Research Institute (IFPRI)	IMPACT
MAGNET	CGE	Wageningen University	MAGNET
MAgPIE	PE	Potsdam Institute for Climate Impact Research (PIK)	MAgPIE

Supplementary Table 4 summarises the 10 models participating in the study, giving key characteristics, time and geographic resolution.

Supplementary Table 4: *Summary of the models participating in the study.*

Model	Type	Description
**AIM**	Computable General Equilibrium (CGE) model	AIM is a one-year-step recursive-type dynamic general equilibrium model that covers all regions of the world. AIM includes 17 regions and 42 industrial classifications. For appropriate assessment of bioenergy and land use competition, agricultural sectors are also highly disaggregated. The production sectors are assumed to maximize profits under multi-nested constant elasticity substitution (CES) functions and each input price.<br><br>Household expenditures on each commodity are described by a linear expenditure system function. The parameters adopted in the linear expenditure system function are recursively updated by income elasticity assumptions. Land use is determined by Logit selection.<br><br>In addition to energy-related CO_2, CO_2 from other sources, CH_4, N_2O, and fluorinated gases (F-gases) are treated as GHGs in the model. Energy-related emissions are associated with fossil fuel feedstock use. The non-energy-related CO_2 emissions consist of land use changes and industrial processes. Emissions from changing land use are derived from the change in forest area relative to the previous year multiplied by the carbon stock density, which is differentiated by AEZs (Global Agro-Ecological Zones).<br><br>For this study, CO_2 emissions and removals from land-use change were considered for carbon stocks in forests.<br><br>Non-energy-related emissions other than those from changing land use are assumed to be in proportion to the level of each activity (such as output). CH_4 has a range of sources, mainly rice production, livestock, fossil fuel mining, and waste management sectors. N_2O is emitted as a result of fertilizer application and

		livestock manure management and by the chemical industry. F-gases are emitted mainly from refrigerants used in air conditioners and cooling devices in the industry. Emissions factors change over time with the implementation of air pollutant removal technologies and relevant legislation.<br><br>Base year: 2005, annual time steps, horizon: 2100<br><br>Number of regions: 17 - Japan, China, India, Rest of East and Southeast Asia, Rest of Asia, EU, Rest of Europe, Former Soviet Union, Turkey, Canada, USA, Brazil, Rest of South America, Middle East, North Africa, Rest of Africa, New Zealand and Australia
**CAPRI**	Partial Equilibrium (PE) model	The CAPRI (Common Agricultural Policy Regionalised Impact) modelling system is an economic large-scale, comparative-static, partial equilibrium model focusing on agriculture and some primary processing sectors (biofuels, dairy and oilseed crushing industries). CAPRI comprises two interacting modules, linking a set of mathematical programming models of EU regional agricultural supply to a spatial multicommodity model for global agrifood markets. The regional supply models depict a profit-maximizing behaviour of representative farms in the European Union and candidate countries, taking into account constraints related to land availability, nutrient balances for cropping and animal activities and policy restrictions. The market module consists of a spatial, comparative-static global multicommodity model for about 60 primary and processed agricultural products, covering 77 countries in 40 trading blocks. Bilateral trade flows and attached prices are modelled based on the Armington assumption of quality differentiation. The behavioural functions in the market model represent supply and demand for primary agricultural and processed commodities (including human and feed consumption, biofuel use, import demand from multilateral trade relations), balancing constraints and agricultural market policy instruments (that is, import tariffs, tariff rate quotas, producer and consumer support estimates, and so on). Depending on scenario specification, behavioural functions are shifted (for example, to reflect productivity shocks or preference shifts) and the model solves for the new market equilibrium.<br><br>With regard to GHG accounting, CAPRI reports EU agricultural GHG emissions for the most important N2O and CH4 emission sources based on the inputs and outputs of agricultural production activities, following to a large extent the 2006 IPCC guidelines (Tier 2 level emission factors in the EU and Tier 1 in non-EU regions). It also considers detailed technical and management-based GHG mitigation options for EU agriculture. GHG emissions for the rest of the world are estimated on a commodity basis in the market model GHG mitigation in non-European countries is represented by a change in emission factors and a matching change in output prices to reflect the increase in cost, derived from mitigation cost functions from the literature. In terms of the database the European data are mostly sourced from Eurostat, while the international data are mostly from the Food and Agriculture Organization, for both model parts supplemented by topic-related sources.

		Base year: 2005, flexible time steps, horizon: 2050<br>Number of regions: 44
**ENVISAGE**	Computable General Equilibrium (CGE) model	Environmental Impact and Sustainability Applied General Equilibrium (ENVISAGE) is a global computable general equilibrium model designed to analyse a variety of issues related to the economics of climate change, as well as broader developmental policies. ENVISAGE is solved as a sequence of comparative static equilibria where the factors of production accumulate over time. The model follows a modular setup, where different modules of the framework can be turned on or off depending on the purpose of the simulations. The version of the model used in the current study is calibrated to the GTAP-Power Data Base v10 with 2014 reference year[3], includes nutritional accounts[4], global food loss and waste data[5], and traces CO2 and non-CO2 greenhouse gases (GHGs), as well as 9 types of air pollutants.<br><br>Mitigation policies in the model are targeting CO2 emissions from fossil fuel combustion, while changes in other GHG and air pollutants are driven by changes in the level of underlying drivers across scenarios (e.g. food demand, fertilizer application etc.). The most recent version of the model documentation is available at: https://mygeohub.org/groups/gtap/envisage-docs.<br><br>Base year: 2014, flexible time steps, horizon: 2100<br>Number of regions: 160 possible, but 20-30 typically
**FARM**	Computable General Equilibrium (CGE) model	The Future Agricultural Resources Model (FARM) is a global computable general equilibrium model used to simulate agricultural adaptation to climate change and options for reducing net greenhouse gas emissions in agriculture, forestry, and energy systems. The FARM model enables active participation in multi-model studies organized by the Agricultural Model Intercomparison and Improvement Project (AgMIP) and the Stanford Energy Modeling Forum. Current applications include simulation of world food demand through 2050, competition for land between energy crops and food crops, and the role of agricultural productivity as an adaptation strategy to climate change. FARM is a recursive dynamic CGE model: essentially a sequence of static equilibria with capital stocks updated between time steps, but lacking foresight of future economic variables. Land use can shift among crops, pasture, and managed forests in response to population growth, changes in income, and other drivers of agricultural activity. Data requirements include a world economic database from the Global Trade Analysis Project (GTAP) at Purdue University, energy balances from the International Energy Agency (IEA), and land use and agricultural production from FAO data. The FARM model operates in five-year time steps from 2011 to 2100, covering 13

		world regions and 38 production sectors. Model output is provided in monetary flows and also quantities: energy flows (joules), agricultural commodities (calories), and land use (hectares).<br><br>Base year: 2011, 5-year time steps, horizon: 2100<br>Number of regions: 13 world regions
**GCAM**	Partial Equilibrium (PE) model	The Global Change Analysis Model (GCAM) is an open-source global multisectoral dynamic and economic equilibrium model that integrates representations of human and physical Earth systems[6]. The model is maintained by the Joint Global Change Research Institute of the Pacific Northwest National Laboratory. GCAM (v7.1) is calibrated to the base year of 2015 and runs dynamically up to 2100 in 5-year steps, driven by future socioeconomic and technological changes. GCAM encompasses 32 world regions and a logit-based Armington approach is used to connect and differentiate regional energy and agricultural markets.<br><br>Agricultural production is modelled at the intersection of 235 water basins and the 32 geopolitical regions. In the base year, GCAM represents the supply and utilization accounts of 21 crop commodities, 6 livestock commodities, and a primary forestry product, providing aggregated representations of all agricultural commodities included in the FAOSTAT database. Additionally, two purpose-grown energy crops (woody and herbaceous) are introduced in 2025. The model includes all land covers and employs a nested logit approach to model their competition, while crop yield at the technology level (i.e., by irrigation and inputs) connects cropland to production[7]. Agricultural greenhouse gas (GHG) emissions in GCAM are activity-based. In particular, CH4 and N2O are traced endogenously as their emission factors are linked to sectoral activities in the agriculture system. The accounting for land use, land-use change, and forestry (LULUCF) emissions relies on emission factors of soil organic carbon and vegetation carbon, which are dynamically applied to land use change results.<br><br>For this study, the recently released version of GCAM (v7.1) was employed, with modifications essential for implementing dietary change and waste reduction scenarios incorporated.<br><br>Base year: 2015, 5-year time step, horizon: 2100<br><br>Number of regions: 32 – Eastern Africa, Northern Africa, Southern Africa, Western Africa, Argentina, Australia New Zealand, Brazil, Canada, Central America and the Caribbean, Central Asia, China, Colombia, EU-12, EU-15, Ukraine, European Free Trade Association, Non-EU Europe, India, Indonesia, Japan, Mexico, Middle East, Pakistan, Russia, South Africa, Northern South America, Southern South America, South Asia, Southeast Asia, South Korea, Taiwan, USA

**GLOBIOM**	Partial Equilibrium (PE) model, Integrated Assessment Model (IAM) (i.e., coupled socioeconomic and biophysical modelling)	The Global Biosphere Management Model (GLOBIOM) is a partial-equilibrium model representing main land use sectors, including agriculture, forestry and biofuels. The supply side of the model is built from the bottom (spatially explicit land cover, land use, management systems and economic cost information) to the top (regional commodity markets). Demand is determined at the level of aggregate regions. Food demand projections are based on the interaction of different drivers: population growth, per-capita income growth, response to prices (based on consumer preferences), and policies.<br><br>The spatial resolution of the supply side relies on the concept of Simulation Units that are aggregates of 5 to 30 arcmin pixels belonging to the same altitude, slope, and soil class, and following country borders. For crops, livestock, and forest products, spatially explicit Leontief production functions covering alternative production systems are parameterized using biophysical models like Environmental Policy Integrated Climate Model (EPIC) or Global Forest Model (G4M). GLOBIOM covers major greenhouse gas (GHG) emissions from Agriculture, Forestry and Other Land Use (AFOLU) based on IPCC accounting guidelines. These include N2O from application of synthetic fertilizer and manure to soils, N2O from manure dropped on pastures, CH4 from rice cultivation, N2O and CH4 from manure management, and CH4 from enteric fermentation.<br><br>For this study, CO2 emissions and removals were considered afforestation, deforestation, wood production in managed forests, and from above- and below-ground biomass changes for other natural vegetation following conversion. Afforestation, deforestation and wood production from managed forests are estimated by the geographically explicit (0.5x0.5 degree) model G4M that is connected to GLOBIOM based on the parameters derived from G4M[8]. For some select cases, GLOBIOM also provides input into G4M[9] which is used to calculate forest emissions. In addition, GLOBIOM endogenously represents GHG mitigation technologies including technological and structural mitigation options.<br><br>Commodity markets and international trade are modelled at the level of aggregate economic regions (the aggregation is flexible and can be adapted to study needs) where prices are endogenously determined at the regional level to establish market equilibrium. Trade is modelled following the spatial equilibrium approach representing bilateral trade flows bases on cost competitiveness and homogeneous good assumption. Besides primary products for the different sectors, the model has several final and by-products, for which, processing activities are defined. The model computes market equilibrium for agricultural and forest products by allocating land use among production activities to maximize the sum of producer and consumer surplus, subject to resource, technological, demand and policy constraints.<br><br>GLOBIOM captures the multiple interrelationships between different systems involved in production of agricultural and forestry products. For example, population dynamics, changes in socio-economic and technological conditions, ecosystems and climate that lead to adjustments in the product mix and the use of land and other

		productive resources. The model is calibrated to an average around the year 2000 and solved recursively dynamic, typically done in 10-year time steps, and depending on the study, going up to 2100.<br><br>Base year: 2000, 10-year time step, horizon: 2100<br>Number of regions: 59 aggregate economic region – all EU27 countries, Rest of Central Europe (RCEU), Rest of Western Europe (ROWE), Russia, Ukraine, Former USSR, Australia, New Zealand, Pacific Islands, United States of America, Canada, Argentina, Brazil, Mexico, Rest of Central America (RCAM), Rest of South America (RSAM), China, Japan, South Korea, Indonesia, Malaysia, Rest of South East Asia - Pacific Countries (RSEA PAC), Rest of South East Asia – Other Pacific Countries (RSEA OPA), India, Rest of South Asian States (RSAS), Middle-East, Northern Africa, Turkey, Congo Basin, Eastern Africa, South Africa, Rest of Southern Africa, West and Central Africa
**IMAGE**	Integrated Assessment Model (IAM) (i.e., coupled socioeconomic and biophysical modelling)	Model description as given in[10]: IMAGE 3.2 is an integrated assessment modelling framework that simulates the interactions between human activities and the environment[11] to explore long-term global environmental change and policy options in the areas of climate, land, and sustainable development. IMAGE consists of various sub-models describing land use, agricultural economy, the energy system, natural vegetation, hydrology, and the climate system. Socioeconomic processes are modelled at the level of 26 regions. Most environmental processes are modelled on the grid-level at 30 or 5 arc-minutes resolution.<br><br>Agriculture, forestry, and land-use dynamics are modelled on the IMAGE-LandManagement model's grid-level[12]. Demand for crop and livestock products, trends in agricultural intensification, and trade dynamics are provided by the economic general equilibrium model MAGNET[13]. Gridded land-use dynamics are implemented in the dynamic global vegetation model LPJmL to model effects on the carbon and hydrological cycle[14,15] and to the global nutrient model (GNM) to model the nitrogen and phosphorus cycles[16]. LPJmL provides data on potential crop and grass yields, land-use change emissions, and irrigation water use. The simulation model TIMER represents the energy system with high technological detail for 12 primary energy carriers, including bio-energy. Land use for the production of bio-energy as determined by TIMER is implemented on the grid-level in IMAGE-LandManagement. GHG emissions from energy, industry, and land use are inputs to the simple climate model MAGICC, which emulates complex climate models to calculate global mean temperature change[17]. The climate policy model FAIR-SimCAP uses MAC curves to determine cost-optimal emission pathways to achieve specific climate targets[18].<br><br>For this study, CO2 emissions and removals (i.e. forest regrowth) from land-use change were considered for carbon stocks in managed forests and peatlands.<br><br>Base year: 2005, time steps: 1-5 year time step, horizon: 2100<br>Number of regions: 26 – Canada, USA, Mexico, Rest of Central America, Brazil, Rest of South America, Northern Africa, Western Africa, Eastern Africa, South Africa, Western Europe, Central

		Europe, Turkey, Ukraine +, Asian-Stan, Russia +, Middle East, India +, Korea, China +, Southeastern Asia, Indonesia +, Japan, Oceania, Rest of South Asia, Rest of Southern Africa
**IMPACT**	Partial Equilibrium (PE) model	The International Model for Policy Analysis of Agricultural Commodities and Trade (IMPACT) was developed in the early 1990s to explore the long-term challenges facing policymakers in reducing hunger and poverty in a sustainable fashion. The IMPACT model has been expanded and improved repeatedly to respond to increasingly complex policy questions and the state-of-the-art of modelling.<br><br>Currently, IMPACT is a network of linked economic, water, and crop models. At its core is a partial equilibrium multi-market economic model, which simulates national and international agricultural markets. The links to water and crop models support the integrated analysis of changing environmental, biophysical, and socioeconomic trends, allowing for in-depth analysis on a variety of critical issues of interest to policymakers at national, regional, and global levels.<br><br>Base year: 2020, annual time step, horizon: 2050<br>Number of regions: 158
**MAGNET**	Computable General Equilibrium (CGE) model	MAGNET (Modular Applied GeNeral Equilibrium Tool) is a recursive, dynamic, multi-regional, multi-commodity computable general equilibrium (CGE) model that simulates the global economy with a focus on agriculture and the bioeconomy. It is based on the GTAP v7 model[19] and the GTAPv11B database, using 2017 as the base year[20].<br><br>Characteristic of a CGE model, MAGNET solves by adjusting prices so that all factor and commodity markets reach equilibrium simultaneously. Producers adjust their input use in response to changes in input prices to maximise profits. With constant returns to scale, producers operate under zero profit conditions. Representative households respond to changes in income from factor sales and to fluctuations in commodity prices by maximising their utility within their income constraint. Bilateral trade flows between all regions are modelled, with regional sourcing of imports based on the Armington assumption.<br><br>Key features of MAGNET relevant to this study include endogenous land markets that capture the dynamics between the supply of agricultural land and real land prices, including the total potentially suitable land available for agriculture[21,22]. In addition, MAGNET incorporates segmented factor markets for labour and capital in the agricultural and non-agricultural sectors, reflecting empirical evidence of imperfect labour mobility as indicated by persistently lower wages in the agricultural sector[23]. Other key features of MAGNET include flexibly nested CES production trees, which

		provide greater substitution possibilities compared to the standard GTAP model, and a purchasing power-adjusted CDE demand function. The latter allows for the adjustment of income elasticities in baseline projections, leading to more realistic patterns of food demand. See also: www.magnet-model.org<br><br>Base year: 2014, flexible time steps, horizon: 2100<br><br>Number of regions: 141 (countries and aggregate country regions)
**MAgPIE**	Partial Equilibrium (PE) model	The Model of Agricultural Production and its Impact on the Environment (MAgPIE) is a modular open-source framework for modelling global land systems[24]. Together with the energy systems model REMIND[25], it forms an Integrated Assessment Modelling (IAM) framework.<br><br>Biogeophysical and spatially explicit input data such as crop yields, carbon stocks and freshwater availability are based on the grid-based dynamic vegetation model LPJmL[14,26], while socio-economic conditions such as factor costs and trade barriers are based on various sources and projections. The above also change (dynamically, depending on the module) as the model runs into the future.<br><br>Food demand is determined by an open-source food demand model[27] that is iterated with MAgPIE in every simulation time step to calculate food intake and availability estimates for different food commodities. The food demand model uses econometric and anthropometric approaches to estimate body height, the distribution of body weight across five body mass index classes, and related food intake based on per-capita GDP, physical activity level, and population data differentiated by sex and age groups.<br>The model projects future land-use patterns and agricultural production at a default spatial resolution of 200 clusters that are located within 12 regions. The model's solution strategy is partial-equilibrium optimization with the goal function of minimizing total cost of production, given economic and biophysical constraints while fulfilling food, feed and biomaterial demand.<br><br>For this study, GHG emissions in MAgPIE include CO2 emissions from land use change (including emissions from vegetation biomass and soil depletion associated with the conversion of forests, peatlands and other natural land into agricultural areas and degradation of forests and peatlands, but also carbon sequestration through forest regrowth and re-/afforestation as well as peatland rewetting[28,29] as well as non-CO2 emissions from crop management (e.g., CH4 from wet rice production, N2O from fertilization) and livestock management (e.g., CH4 and N2O from animal waste management and CH4 from enteric fermentation). N2O and CH4 emissions are converted to CO2 equivalents using the IPCC AR6 GWP 100 factors of 273 for N2O and 27 for CH4[30].<br><br>Base year: 2005, 5-year time steps, horizon: 2100<br><br>Number of regions: Flexible with country level as smallest unit. Standard setup has 12 aggregated regions.

Model implementation of the main scenario components

Supplementary Table 5 provides a description of how each of the models implemented the three key scenario components (dietary change, improved productivity, reduction in food loss and waste).

Supplementary Table 5: *Model descriptions of implementation of scenario components.*

Model	Dietary change	Improved productivity	Food loss and waste reduction
AIM	In the AIM model, food demand is analysed using linear expenditure system (LES) functions, which assume that income and price elasticities are interconnected. The model bases its food consumption income elasticity on two sources. The first is historical data; if a consistent relationship between income levels and food consumption is identified, AIM uses this relationship as a foundational principle. For other goods, we follow the perspective of the FAO[31]. The income elasticity parameters are differentiated across 17 regions. In AIM, food demand is categorized into six groups based on crops and plants: wheat, rice, other grains, oilseed crops, sugar crops, and vegetables, fruits, and nuts. Additionally, there are three groups for livestock and animal-sourced foods: ruminant livestock, milk, and non-ruminant livestock and fishery items. Dietary changes are represented by adjusting and calibrating the income elasticity parameters to align with the specified EAT-Lancet diets by the target year of 2050.	In the AIM model, assumptions regarding technological changes that affect crop yields are dependent on Shared Socioeconomic Pathways (SSPs). The model incorporates exogenous crop yields as inputs and calculates endogenous crop yields based on crop production divided by the area cultivated. Technology development and irrigation expansion are considered key factors influencing future yield changes. Yield changes resulting from technological advancements align with the range of estimates found in existing research, as guided by the SSPs. The model assumes variations in yield for both rain-fed and irrigation-fed crops, based on well-established assumptions from AgMIP as outlined in ref [32]. The changes in yield due to irrigation expansion are determined by irrigation growth rate estimations from ref [33]. Further details can be found in ref [34]. Productivity increases for crops are projected in line with SSP1 assumptions. For pasture productivity, improvements are	In the AIM model, food loss and waste are considered through commodity- and region-specific ratios, which adjust total demand to account for consumer-level waste. The parameters related to food loss and waste are based on data provided by ref [35]. By incorporating food loss and waste into the overall demand calculation, reducing these losses ultimately leads to a decreased demand for agricultural products. This reduction lowers the amount of land use required at the production stage. To illustrate the impact of food loss and waste reduction in this study, the waste ratio is adjusted multiplicatively until food loss and waste are halved.

		assumed to result from changes in livestock demand, which are calculated based on GDP growth, income elasticity, and population changes.	
CAPRI	Like most sectoral simulation models, CAPRI refers to quantities available at the market level for human consumption. Using rates of loss and waste in distribution (retail) and households provided by the FAO[35], individual intake quantities are calculated as ex-post reporting. The loss and waste rates are ad hoc corrected to reach plausible per capita energy intake levels (e.g., between 2,000 and 2,500 kcal in Europe)[36]. The resulting per capita intake in grams is transferred into calories, serving as a baseline for calculating scenario shocks of dietary changes.<br><br>To simulate the society-wide dietary shifts in CAPRI by adjusting the market balance quantities available for human consumption, the individual energy intake per commodity and region recommended in the EAT-Lancet healthy reference diet is compared to the CAPRI baseline, and the relative change is applied as a shock to shift the demand curves[37]. This means that human consumption aligns with the EAT-Lancet diet for prices at baseline levels.<br><br>The exogenous shock to the demand curve triggers endogenous reactions in the model, adjusting prices until a new supply-demand equilibrium is reached. Consumers react to	The CAPRI baseline captures GDP growth in a productivity multiplier[38]. In the scenarios containing climate-change-induced productivity changes, these are added to the baseline productivity changes, resulting in a combined multiplier used for exogenous shocks.<br><br>For EU countries, the shock is applied to yields which are endogenously modelled. They increase as a reaction to increasing producer prices, accounting for the incentives higher prices give producers to intensify production.<br><br>For non-EU countries, modelled without endogenous yields, the supply curve is shifted.	The reduction in food loss and waste is applied to the loss and waste shares in retail and households. These shares are sourced from the FAO[35] and ad-hoc corrected in the CAPRI baseline to reach plausible per capita energy intake levels[36].<br><br>In the scenarios, these shares are exogenously adjusted to simulate the reduction of loss and waste.<br><br>The reductions in loss and waste do not lead to an increased food intake by the consumers. Therefore, we adjust the quantities available for human consumption (consisting of loss, waste, and intake quantities). Demand curves are shifted to capture the reduction in loss and waste and to keep intake quantities constant.<br><br>As with the dietary component, the exogenous shock to the demand curve triggers endogenous reactions in the model, adjusting prices until a new supply-demand equilibrium is reached.

	price changes by adjusting demand. Therefore, the intake levels recommended in the EAT-Lancet reference diet are not met precisely for all commodities and all regions.		As consumers also react to price changes by adjusting demand, the baseline intake levels are not precisely met for all commodities and regions.
ENVISAGE	The baseline scenario assumes several modifications related to the representation of food- and nutrition-related aspects of the model. First, modifications to the Constant-Differences-of-Elasticities (CDE) consumer demand system parametrization have been implemented. In particular, following ref [13] the parametrization relies on the econometrically estimated relation between income elasticities and per capita income. The second adjustment is related to the representation of food and agricultural systems in the baseline scenario and includes updating shares of the primary food inputs to the processed food and service activities. Improvements vary by country and commodity and are on average between 1% and 2% per year. Finally, direct shifts in the final demand patterns are implemented. These adjustments have been introduced to better reflect the future food demand trends in the model. To achieve the dietary targets, two key channels are used. First, adjustments in the CDE share parameters capture preference changes by final consumers. Second, changes in the intermediate demand shares by food processing and out-of-home food consumption	In the baseline scenario, a labour augmenting technical change is assumed. Labour productivity is calibrated to given assumptions about GDP growth, based on the projections from the SSP2 scenario. For crop yields, livestock productivity, as well as agricultural labour, specific assumptions on productivity changes are based on the data provided to the modelling teams by study co-authors. Corresponding shocks have been implemented as exogenous productivity changes within the baseline scenario and correspond to the Representative Concentrations Pathway (RCP) 7.0 case, thus incorporating the impacts of climate change. For the high-productivity scenario, livestock productivity trends and crop yields follow the assumptions of the SSP1 scenarios.	Food loss and waste (FLW) reductions are implemented using efficiency improvements across countries, commodities and supply stages. Five FLW stages distinguished in ref [5]– agricultural production, postharvest handling and storage, manufacturing, distribution and retail, and consumption – are represented via two types of efficiency improvements in the model. First is the efficiency improvement in direct food consumption by final users and the second is an improvement in the intermediate use efficiency. Corresponding efficiency improvements are introduced starting from 2021 and are fully implemented by 2050 following a linear reduction in the FLW shares over the 30-year period (i.e. a 50% reduction is fully achieved in 2050). Corresponding efficiency improvements are not associated with any

	activities share of value-added and other inputs in the corresponding sectors. These shocks are implemented in the model to achieve the lower/upper bounds of per-capita food intakes for 2050 (in g/cap/day) of the EAT-Lancet healthy reference diet. In addition, targeted caloric intake is closely matched in 2050.		additional costs or investments in the model. It should be noted that when corresponding shocks are implemented, they lead to changing prices, composition of food demand, trade, production patterns, etc. When all these effects are taken into account, the resulting changes in FLW do not exactly equal 50%, as there are endogenous adjustment mechanisms determined by the model following the market-driven processes discussed above.
FARM	Food consumption for 14 food commodities is calibrated to benchmark year (2011) FAO food balance sheets[39]. FAO provides food consumption data of a mass basis within a country, which are converted to calories per person per day for each food commodity.<br><br>In business-as-usual (BAU) scenarios, food consumption is determined by the Linear Expenditure System (LES), which allows per capita food consumption to respond to changes in income. As incomes increase, the general pattern is an increase in total calories per person and a shift toward consumption of animal products and vegetable oils[40]. Demand system parameters are calibrated so that per capita food calorie consumption in 2050 approximates projections by the FAO[41].<br><br>In the EL2 scenario, future diets are predetermined by world region to 2050 based on	Technical change parameters vary over time to construct a plausible global reference scenario for energy, agriculture, and land use through 2050. All technical change parameters are input specific and are considered as input augmenting. For example, labour productivity parameters were used to produce GDP pathways for each region that closely approximate target pathways from SSPs. These parameters vary over four dimensions: model time step, input to production, production sector, and world region. Productivity improvements are reflected in input-output ratios that decline over time.	Three types of calorie measures are computed in each scenario: production of crop calories, food calories available for consumption, and food calories consumed. Crop calories are always greater than available food calories due to conversion losses from feed to animal products. FAO data correspond to food calories available for consumption. Available food calories are always greater than calories consumed because of FLW.<br><br>In each FLW scenario, the gap between available food calories and food calories consumed is reduced; final consumption of food does not change. A scenario with 50% reduction in FLW

	EAT-Lancet healthy reference intakes. Consumer diets change linearly from historical food consumption to targets by food commodity in 2050.	Each land-using production function (e.g., wheat, rice, coarse grains) has a technical coefficient associated with land, which is the primary determinant of yield growth over time. Crop yield is also influenced by changes in prices of agricultural products and the substitution of non-land inputs for land. Therefore, simulated crop yield in the FARM model is a combination of productivity growth and price-induced shifts across inputs to production.	reduces food calories available for consumption so the gap with calories consumed is reduced by half. In this analysis, the reductions are costless. With a reduction in available food calories, production of crop calories is reduced along with reductions in land required for crops.
GCAM	GCAM employs a calorie-based food demand model that incorporates own- and cross-price elasticities for both staple and non-staple food groups[42]. Substitution patterns within these groups are represented using a nested logit structure. FAO food commodities are aggregated into 15 crop and 6 livestock products, with their calorie content forming the basis of the demand model[43,44]. To implement the dietary target by a future period, target consumption values are first harmonized and mapped to specific GCAM food groups (i.e., staple crops, sugar, oils, fruits and vegetables, plant protein, ruminant meat, dairy, non-ruminant meat, and fish and other meat), with a logit function aggregating the underlying GCAM commodities within each group. These dietary targets are then converted into regional and commodity-group-specific shock trajectories—linearly interpolated relative to 2020 levels—which are applied exogenously in GCAM food systems to drive future dietary	Productivity growth and technological advancement in agricultural production are implemented in GCAM as exogenous changes in factor productivity for both crop and livestock sectors, based on input data provided to modelling teams by study co-authors. For crops, future yield improvements are applied as scalers to land productivity—i.e., increasing output per unit of land input. For livestock, productivity growth is represented as improvements in feed conversion efficiency.<br><br>Specifically, GCAM aggregates all FAO crops into 21 plant functional types (PFTs), with production further differentiated by irrigation type (rainfed vs. irrigated) and input intensity (high vs. low input technologies). After model calibration to the	GCAM represents food loss and waste as the wedge between food calorie supply and intake by linking dietary energy consumption to food supply using a recently compiled Food Balance Sheet[44] and EAT-Lancet healthy reference diet intake data. Waste shares are differentiated across three groups: livestock products, fruits and vegetables, and other crops.<br><br>In the BAU scenario, future changes in regional waste shares are driven by per capita income growth and are implemented alongside changes in calorie conversion efficiency (i.e., the ratio of calories supplied or consumed per unit mass of food). To implement a food loss and waste reduction target, e.g., a 50% reduction by 2050, we

	energy demand, reflecting preference shifts over time. The calorie intake of food products within each commodity group is determined endogenously, with dietary energy intake linked to food supply in mass units. This linkage aligns with the structure of food balance sheets and supply utilization accounts, and it plays an integral role in global and regional market clearing within GCAM.	base year (2015), future yield trajectories at the technology level are driven by exogenous productivity scalers mapped from study input data, available for both BAU and higher-productivity scenarios. Similarly, GCAM includes six livestock sectors, each characterized by either pastoral or mixed-feed systems. Feed conversion ratios, calibrated to the base year, are also updated using exogenous shocks derived from study inputs.	construct a trajectory of declining waste shares over time. These are then operationalized as improvements in calorie conversion efficiency, reflecting enhanced utilization of available food supply.
GLOBIOM	In GLOBIOM, regional food demand is modelled based on population, income, prices, and consumer preferences. Population and income growth are exogenous, while prices are endogenous. Preferences are represented by exogenous income and price elasticities that vary over time depending on a country's development and the dietary scenario. The base year for consumption levels is 2000, using FAO Food Balance Sheets to anchor projections.<br><br>Price elasticities in the base year align with USDA econometric estimates[45] and gradually converge to US levels with rising income. Income elasticities are calibrated to meet three conditions: (1) matching base year USDA estimates, (2) ensuring per capita calorie consumption aligns with scenario assumptions, and (3) reflecting dietary composition in line with scenario-based preferences (e.g., meat type, sugar, and fat share).	GLOBIOM explicitly covers the production of 18 major crops globally. Each crop can be produced under different production systems that differ in productivity and cost.<br><br>Crop yields are allowed to change endogenously based on external drivers through switching from one production system to another, or reallocation of the production to a grid cell with different productivity. Aside from the endogenous mechanisms, an exogenous component that represents long-term technological change is considered. The model also includes a detailed representation of the global livestock sector, incorporating multiple production systems, with grassland productivity explicitly modelled.	Food loss and waste (FLW) in GLOBIOM is exogenous and it is represented as a fraction of total production and consumption. Reduction in food loss and waste results in higher supply or lower demand of food products. This means that there is less land and other natural resources involved.<br><br>The amount of loss and waste vary by region, crop and animal product, and time period (based on ref [35]. The regional and product specific shares of FLW are kept constant over time in the BAU scenario. For the EL2 scenario, SSP1 rates of loss reduction was assumed. It has been estimated based on a regression linking per capita GDP assumed in SSP1 and food losses. Household level waste was

	Food groups in the EAT-Lancet healthy reference diet were mapped to GLOBIOM food groups, with recommended quantities expressed in calories. These values guide the calibration of income elasticities so that diets align with the EAT-Lancet targets by 2050.		assumed to be linearly reduced by 50% by 2050.
IMAGE	IMAGE is coupled to the MAGNET model for the representation of the agro-economy, including food demand. Therefore, the dietary change implementation is directly in line with the MAGNET implementation.<br><br>Diet targets for the EAT-Lancet healthy reference diet food groups are derived by comparing 2050 BAU intake in grams/capita to recommended levels. Targets for staples (cereals, roots and tubers) are adjusted such that the EAT-Lancet healthy reference diet recommended calorie intake is reached. These targets are set exogenously in the diet scenario, allowing MAGNET to endogenously select a consumption pattern where total intake (fresh and processed foods, food consumed out-of-home through food services) satisfies EAT-Lancet recommendations. Defining the targets in primary equivalents instead of food items allows region-specific diet adjustments in line with food preferences and endogenously changing prices. Non-food consumption adjusts endogenously to meet the	Agricultural productivity increases are based on an SSP1 scenario[48]. SSP1 describes a sustainable world characterized by improvements in both crop yields and production efficiencies.<br><br>In IMAGE, production and resource efficiencies (i.e., how much dry matter feed is required to produce one unit of animal output) are adjusted for pig, poultry, beef, and dairy production systems consistent with SSP1[48]. This results in lower feed requirements, and increased production for all of these livestock groups. Crop yield and livestock efficiency improvements are exogenously implemented in MAGNET, using region-specific assumptions about technological in technology parameters of the nested CES production functions in MAGNET. This implementation approach	IMAGE is coupled to the MAGNET model for the representation of the agro-economy, including food loss and waste (FLW). Therefore, the FLW implementation is directly in line with the MAGNET implementation.<br><br>Reductions in FLW are defined by groups of food items to capture differences in FLW rates, with a method based on ref [51]. Food loss and waste are implemented at three different stages of the food supply chain (primary, processing and consumption) for five food categories (cereals, meat, fish and other seafood, milk and eggs, and other plant-based foods). Reduction in food loss and waste are based on estimations by ref [35] and mapped from their food categories (cereals, oilseeds and pulses, roots and tubers, fruits and

	(endogenous) household budget constraint.<br><br>The primary content of food items is tracked by a variable initialized through computing the Leontief Inverse (LI) on the material balances[46,47]. Intermediates used in primary food production are excluded, (e.g. feed inputs are excluded from the primary content of beef). The LI traces all primary contents added in different stages of the global supply chains, which is key to assess intake through composite processed foods and food services. During simulations the LI-derived primary content is updated to capture changes in region-specific intermediate input use and bilateral trade flows.	was used in for example in refs [49,50]. These yield changes consequently influence land-use demand by altering regional crop production levels.	vegetables, meat, milk) and regions (Europe and Russia, Industrialised Asia, North America and Oceania, Latin America, North Africa and West and Central Asia, South and Southeast Asia, Sub-Saharan Africa) to IMAGE-MAGNET food categories and regions. Maximum reductions are assumed by taking the lowest FLW rates observed in low-income world regions, and high- and middle-income regions can converge to those levels.<br><br>As FLW amounts are not explicit, these reductions are modelled as efficiency gains, i.e. less food items are needed to produce the same amount of end product or final consumption.
IMPACT	Dietary change in the IMPACT model is implemented by providing additional adjustments to the exogenous household demand growth rates to achieve alignment with the EAT-Lancet healthy reference diet. The baseline simulation (BAU) follows SSP2 assumptions for population, income growth, and technological change, generating endogenous projections of food demand by commodity and region. To implement the EL2 scenario, a demand gap is calculated by comparing the baseline per capita availability in the baseline scenario to the EL2 diet targets by 2050. These gaps, expressed as deviations from the baseline, are applied	Crop productivity is primarily driven by exogenous assumptions about Intrinsic Productivity Rates (IPRs), which reflect expected annual yield growth rates due to technological progress, management improvements, and other non-price factors[52]. IPRs are informed by expert consultations, including global model intercomparisons[53], CGIAR scientist input[54], and empirical trends in yield growth and research intensity, often benchmarked against FAOSTAT data.	In the IMPACT model, food loss and waste (FLW) are incorporated through commodity- and region-specific waste factors that adjust total demand to account for consumer-level waste. These waste factors are applied multiplicatively to projected agricultural demand, effectively simulating an increased gross production required to meet net demand. Thus, reductions in FLW translate into lower gross demand for agricultural commodities.

	to shift household demand growth trajectories in IMPACT to meet the desired EL2 target over 30 years from start of IMPACT simulations (2020), gradually transitioning toward EL2 targets by 2050.<br><br>The adjusted demand trajectories are introduced as exogenous inputs to the model, effectively modifying the income- and price-responsive demand system for the affected commodities, which need demand adjustments based in EL2 diets. The model is then rerun with these (re)adjusted trajectories to simulate the resulting impacts on production, trade, prices, nutrition etc. This method maintains consistency within the partial equilibrium framework while enabling the evaluation of alternative diet futures. By isolating demand-side shifts, the approach allows for assessment of the biophysical and economic implications of large-scale dietary transitions under a range of policy and development scenarios.	In IMPACT, IPRs vary by country and modelled crop. For the EL2 scenario, productivity changes (increases) are implemented as proportional adjustments to baseline IPRs. These adjustments are linked to differences in per-capita income between SSP1 and SSP2, under the assumption that higher per-capita income levels under SSP1 correlate with greater public and private investment in agricultural R&D and extension. The adjusted IPRs gradually increase productivity relative to the baseline in high productivity scenarios. This approach allows the model to simulate the systemic implications of accelerated yield improvements on supply, prices, area harvested, yields, and trade in the context of dietary transformation scenarios.	To simulate FLW reduction scenarios, the waste factors are exogenously modified relative to the baseline (50% reduction). This reduction lowers the quantity of food required at the production stage, which in turn reduces pressure on agricultural supply system (area, yields etc.). Because IMPACT is a partial equilibrium model, changes in demand from waste reductions also affect market outcomes—lowering prices, shifting trade balance.
MAGNET	Diet targets for the EL2 food groups are derived by comparing 2050 BAU intake in grams/capita to recommended levels. Targets for staples (cereals, roots and tubers) are adjusted such that the EL2 recommended calorie intake is reached. These EL2 targets are set exogenously in the diet scenario, allowing MAGNET to endogenously select a consumption pattern where total intake (fresh and processed foods, food consumed out-of-home through food services) satisfies the EL2 recommendations. Defining the targets in primary equivalents	The increase in productivity is quantified using data for the SSP1 scenario provided by the IMAGE model (see ref [48] for more details on the IMAGE implementation). Region- and sector-specific increases in productivity of land in crop production and feed in livestock production are modelled by exogenous changes in technology parameters of the nested CES production functions in MAGNET. This implementation approach	Reductions in food loss and waste (FLW) are defined by groups of food items (cereals, fish meat, dairy, other foods) to capture differences in FLW rates. The method is based on the application as applied in ref [51]. Food loss and waste are implemented at three different stages of the food supply chain (primary, processing and consumption) for five food categories (cereals, meat, fish and other seafood, milk and

	instead of food items allows region-specific diet adjustments in line with food preferences and endogenously changing prices. Non-food consumption adjusts endogenously to meet the (endogenous) household budget constraint.<br><br>The primary content of food items is tracked by a variable initialized through computing the Leontief Inverse (LI) on the material balances[46,47]. Intermediates used in primary food production are excluded, (e.g. feed inputs are excluded from the primary content of beef). The LI traces all primary contents added in different stages of the global supply chains, key to assess intake through composite processed foods and food services. During simulations the LI-derived primary content is updated to capture changes in region-specific intermediate input use and bilateral trade flows.	was used for example in refs [49,50].<br><br>This implementation increases productivity of livestock via two channels: directly by saving on feed use and indirectly as crop-based feed inputs are becoming cheaper from the increased productivity of land.	eggs, and other plant-based foods).<br>Reduction in food loss and waste are based on estimations by ref [35] and mapped from their food categories (cereals, oilseeds and pulses, roots and tubers, fruits and vegetables, meat, milk) and regions (Europe and Russia, Industrialised Asia, North America and Oceania, Latin America, North Africa and West and Central Asia, South and Southeast Asia, Sub-Saharan Africa) to IMAGE-MAGNET food categories and regions. Maximum reductions are assumed by taking the lowest FLW rates observed in low-income world regions, and high and middle income regions can converge to those levels.<br><br>As FLW amounts are not explicit, these reductions are modelled as efficiency gains, i.e. less food items are needed to produce the same amount of end product or final consumption.<br><br>To capture food losses in production, efficiency gains are applied at total activity level for food sectors (the same level of inputs produces more food), and at intermediate input level for food processing and food services sectors. Use of food in non-food sectors is not adjusted. At the household level food waste is captured

			by a shift in the demand function is applied which reduces the food demanded by the household.
MAgPIE	As opposed to a BAU diet scenario, where food consumption patterns in MAgPIE follow historical trends[27], food consumption in the EL2 scenario component is constrained by the intake recommendations from the EAT-Lancet healthy reference diet[55]. These recommendations are applied at the country level and define upper and lower bounds for different food groups, which were mapped to the corresponding food categories in MAgPIE.<br><br>The endogenously calculated country-level intake per MAgPIE food item from the BAU scenario is increased (when the minimum intake target is not reached) or reduced (when the maximum intake target is exceeded). If these adjustments result in a deviation from the total kilocalorie intake required by the EAT-Lancet reference diet, the food group 'cereals' (balancing post) is adjusted. The transition to the recommended intake levels is phased in gradually to 2050.	Improvements in crop yields are determined endogenously in MAgPIE based on the interplay between land-use intensity and technological change costs. Depending on other model constraints (e.g., scarcity of land or other resources given demand), MAgPIE invests into technological change to increase land-use intensification, which in turn increases yields. The effectiveness of such investment decreases with the intensity level as further increases will become more expensive as more technical solutions have already been implemented.<br><br>Following the implementation in ref [56], yield increases in the EL2 scenario component are facilitated by reducing interest rates in developing countries. In low-income regions, interest rates are lowered from 10% to 6%. Interest rates in high-income countries remain at 4%. This improves the economic feasibility of investments in yield-increasing technologies.<br><br>In the EL2 scenario, livestock productivity follows SSP1 assumptions. Compared to increases of livestock	For historical time steps, food waste is calculated at country level by subtracting estimated per-capita food calorie intake (averaged across age and sex groups) from FAO per-capita food calorie supply[27,39]. For future time steps, the increase in the food waste ratio (defined as the ratio of food calorie supply to food intake) is estimated using a regression[27]. The relative contribution of different food products to estimated per-capita food waste is inferred from food-group specific waste estimates from ref [35].<br><br>In the EL2 scenario, a reduction of food waste to a maximum of roughly 50% of the levels observed in high-income regions is enforced by 2050 at the country level. This reduction is phased in gradually to 2050.

		productivity in BAU (SSP2), the SSP1 setting assumes a further catch-up in regions with currently low productivity levels, with a focus on ruminant production systems. Productivity gains involve both more efficient feed conversion as well as a change in feed basket composition, shifting from feed sources with a low nutrient content such as grazed biomass or crop residues to feed types with a higher nutrient content.	

Protocol reporting

Food sector representation

Supplementary Table 6 shows the sectors reported on by the model ensemble.

Supplementary Table 6: Sectors represented in the modelling. *Different models have different resolution and aggregation of sectors. The list here represents a common set of sectors to be reported on.*

**Group**	**Sector code**	**Full name**
Agriculture	AGR	All agricultural products
Livestock products	LSP	RUM + NRM + DRY + OAP
Animal sourced foods	RUM	Ruminant meats
	RUM|BOV	Bovine meat
	RUM|OTH	Other ruminant (e.g. sheep, goat) meat
	NRM	Non ruminant meats (including eggs)
	NRM|PRK	Pig meat
	NRM|PTM	Poultry meat
	DRY	Dairy
	OAP	Other animal products (wool, honey)
Crops	CRP	RIC + WHT + CGR + OSD + SGC + VFN + PFB + OCR<br>PFB = plant-based fibres, OCR = other crops
Cereals	RIC	Rice
	WHT	Wheat
	CGR	Coarse grains (including maize)
Vegetables, fruits, nuts, legumes, roots and tubers	VFN	Vegetables, fruits, nuts, and legumes
	VFN|VEG	Vegetables
	VFN|FRU	Fruits

	VFN|NUT	Nuts
	VFN|LEG	Legumes
	VFN|RTB	Roots and tubers
Oils, fats, and sugar	OSD	Oilseeds (including soybeans and peanuts)
	SGC	Sugar crops
Land-based sectors	FOR	Forest products
	GRS	Grass
	ONV	Other natural vegetation
	ECP	Energy crops

Model reporting

Not every model reports on every possible sector or variable. Supplementary Table 7 shows the sector-variable coverage of models in the ensemble.

Supplementary Table 7: Number of models (out of 10) reporting results for each food sector and variable combination. X indicates not required. *XPRP = producer prices, PROD = production, AREA = harvested area, ANNR = animal numbers, FOOD = food use, FEED = feed use, OTHU = other use, AEMP = Agricultural employment, LAND = physical land, EMIS = non-CO2 (direct) emissions, WATR = water withdrawals, FRTN = nitrogen fertilizer.*

Sector	XPRP	PROD	AREA	ANNR	FOOD	FEED	OTHU	AEMP	LAND	EMIS	WATR	FRTN
AGR	8	9	6	X	8	8	8	3	6	5	3	2
GRS	X	X	X	X	X	X	X	X	6	X	X	X
CRP	8	9	8	X	8	9	8	2	6	5	7	4
RIC	9	10	9	X	8	9	8	1	X	5	5	4
WHT	9	10	9	X	9	9	8	1	X	5	5	4
CGR	9	10	9	X	9	9	8	1	X	5	5	4
VFN	7	9	9	X	8	9	8	1	X	5	5	3
OSD	9	10	9	X	9	9	8	1	X	5	5	4
SGC	9	10	9	X	8	9	8	1	X	5	5	4
LSP	8	9	4	X	9	5	8	2	X	5	X	X
RUM	8	9	4	2	9	4	7	1	X	5	X	X
NRM	8	9	X	2	9	4	8	1	X	5	X	X
DRY	8	9	4	2	9	5	8	1	X	5	X	X
OAP	2	3	1	X	2	1	3	1	X	X	X	X

Reference diet

Supplementary Table 8 shows a summary of the EAT-Lancet healthy reference intake in both grams and kcal per person per day.

Supplementary Table 8: Reference intake for healthy diets provided to all participating models based on an updated version of[57]*. Model mapping is the illustrative categorisation of nutritional food group to sector commodities. Note that, nutritionally, pig meat is classified as red meat (i.e. along with bovine and sheep meat), but*

pigs are commonly biophysically grouped with other monogastric (non-ruminant) animals. The conversion from g/day to kcal/day uses FAO Food Balance Sheets.

Food group	Model mapping	g/day	kcal/day
Grains	RIC, WHT, CGR	233	630
Roots	VFN	50	43
Vegetables	VFN	300	76
Fruits	VFN	200	105
Legumes	VFN	100	350
Nuts and seeds	VFN	50	182
Vegetable oils	OSD	54	511
Palm oil	OSD	6	58
Sugar	SGC	30	96
Red meat	RUM, NRM	15	34
Poultry	NRM	30	43
Milk	DRY	250	190
Eggs	NRM	15	21
Fish	FSH	30	18

Exogenous yields

Supplementary Table 9 shows the global average exogenous yield trends between 2020 and 2050 for different sectors and the two core scenarios (BAU and EL2).

Supplementary Table 9: Global average exogenous yield trends (inclusive of productivity changes and climate impacts) for crops and livestock products provided by IMPACT model for use in EL2 scenario. *RUM = ruminant meat, DRY = dairy, NRM = non ruminant meat (poultry, pork, eggs), RIC = rice, WHT = wheat, CGR = coarse grains, VFN = vegetables, fruits, and nuts, OSD = oilseeds, SGC = sugar crops, OCR = other crops, PFB = plant-based fibres.*

Sector	BAU yield trend 2020-2050 (%)	EL2 yield trend 2020-2050 (%)
RUM	25	37
DRY	39	54
NRM	35	48
RIC	31	48
WHT	48	61
CGR	36	48
VFN	30	45
OSD	34	47
SGC	20	33
OCR	29	44
PFB	29	43

Global region coverage

Supplementary Table 10 shows the categorisation (at ISO3 level) of countries into the 13 global regions represented in the modelling.

Supplementary Table 10: *The thirteen global regions (plus world) and their countries represented in the reporting protocol.*

Region	Full name	ISO3
CAN	Canada	CAN
USA	United States	USA
BRA	Brazil	BRA
CHN	China	CHN
IND	India	IND
OSA	Other South and Central America	AIA, ATG, ARG, ABW, BHS, BRB, BLZ, BMU, BOL, VGB, CYM, CHL, COL, CRI, CUB, DMA, DOM, ECU, SLV, FLK, GRD, GLP, GTM, GUY, HTI, HND, JAM, MEX, MSR, ANT, NIC, PAN, PRY, PER, KNA, LCA, VCT, SUR, TTO, TCA, URY, VEN, GUF, PRI, VIR
EUR	Europe	ALB, AND, AUT, BEL, BIH, BGR, HRV, CYP, CZE, DNK, EST, FIN, FRA, DEU, GIB, GRC, VAT, HUN, ISL, IRL, ITA, LVA, LIE, LTU, LUX, MLT, MCO, MNE, NLD, NOR, POL, PRT, ROU, SMR, SRB, SVK, SVN, ESP, SWE, CHE, MKD, GBR, GRL, SJM, IMN, JEY, GGY
ECC	Eastern Europe, Caucasus, Central Asia	ARM, AZE, BLR, GEO, KAZ, KGZ, MDA, RUS, TJK, TKM, UKR, UZB
MEN	Middle East and North Africa	DZA, BHR, EGY, IRN, IRQ, ISR, JOR, KWT, LBN, LBY, MAR, OMN, PSE, QAT, SAU, SYR, TUN, TUR, ARE, YEM, ESH
SSA	Sub-Saharan Africa	AGO, BEN, BWA, BFA, BDI, CMR, CPV, CAF, TCD, COM, COG, CIV, COD, DJI, GNQ, ERI, ETH, GAB, GMB, GHA, GIN, GNB, KEN, LSO, LBR, MDG, MWI, MLI, MRT, MUS, MOZ, NAM, NER, NGA, REU, RWA, STP, SEN, SYC, SLE, SOM, ZAF, SDN, SWZ, TGO, UGA, TZA, ZMB, ZWE
SEA	South-East Asia	BRN, KHM, PRK, IDN, JPN, LAO, MYS, MMR, PHL, KOR, SGP, THA, TLS, VNM, TWN
OAS	Other Asia	AFG, BGD, BTN, COK, FJI, PYF, KIR, MDV, MHL, FSM, MNG, NRU, NPL, NCL, NIU, PAK, PLW, PNG, WSM, SLB, LKA, TKL, TON, TUV, VUT
ANZ	Australia and New Zealand	AUS, NZL
WLD	World	All countries

Supplementary Discussion

Literature review summary

This review follows the interim guidance on PRISMA rapid reviews (PRISMA-RR)[58] and focuses on multi model ensembles (MMEs) and model intercomparison projects (MIPs) that have explored historic or future scenarios of the socioeconomic or environmental dimensions of agricultural or food system change. A second review was conducted on single model/non-MME studies. The reviews exclude studies on climate or meteorological models and those focused specifically on the climate impacts on crops. Scopus and Web of Science Core Collection (WoS) were searched in June 2025 (see Supplementary Table 11 for search string). All languages, time periods, and document types were included in the search. To expedite the review, title and abstract screening and extraction was performed by the lead author using Covidence.

Supplementary Table 11: *Search strings for the MME and single model/non-MME rapid reviews.*

Review	Search string
MME	("multi model ensemble" OR "multi-model ensemble" OR "model ensemble" OR "modelling ensemble" OR "model intercomparison" OR "multi-model" OR "multi model") AND ("EAT-Lancet" OR "EAT Lancet" OR "diet*" OR " food AND system* " OR "healthy AND diet*" OR "agriculture" OR "land" OR "economic" OR "histor*")
Single model/ non-MME	("model*" OR "modelling") AND ("EAT-Lancet" OR "EAT Lancet" OR "diet*" OR " food AND system* " OR "healthy AND diet*" OR "agriculture") AND ("scenario*") AND ("global")

MME literature review

After removing duplicates (n=151), when combining Scopus (n=536) and WoS (n=375) searches and citation searching (n=9), 768 studies were screened for title and abstract. Fifty-nine studies were included for full text screening with 20 included for extraction (see Supplementary Figure 2). The main reasons for exclusion were studies that were concerned with the energy/power sector only (n=11), those focused on carbon pricing, emissions budgets or economic damage/impact/welfare (n=6), studies which were not MMEs/MIPs (n=6), and studies on crop yields due to climate impacts (n=5).

Single model/non-MME literature review

After removing duplicates (n=328) when combining Scopus (n=869) and WoS (n=822) searches and citation searching (n=13), 1376 studies were screened for title and abstract, 105 studies were included for full text screening with 55 included for extraction (see Supplementary Figure 3). The main reasons for exclusion were studies that did not explore models or scenarios (n=8), studies climate impacts e.g. on crop yields (n=8), and studies focusing on biodiversity/forestry (n=6) or bioenergy only (n=6).

The studies are summarised in Supplementary Table 12 and Supplementary Table 13.

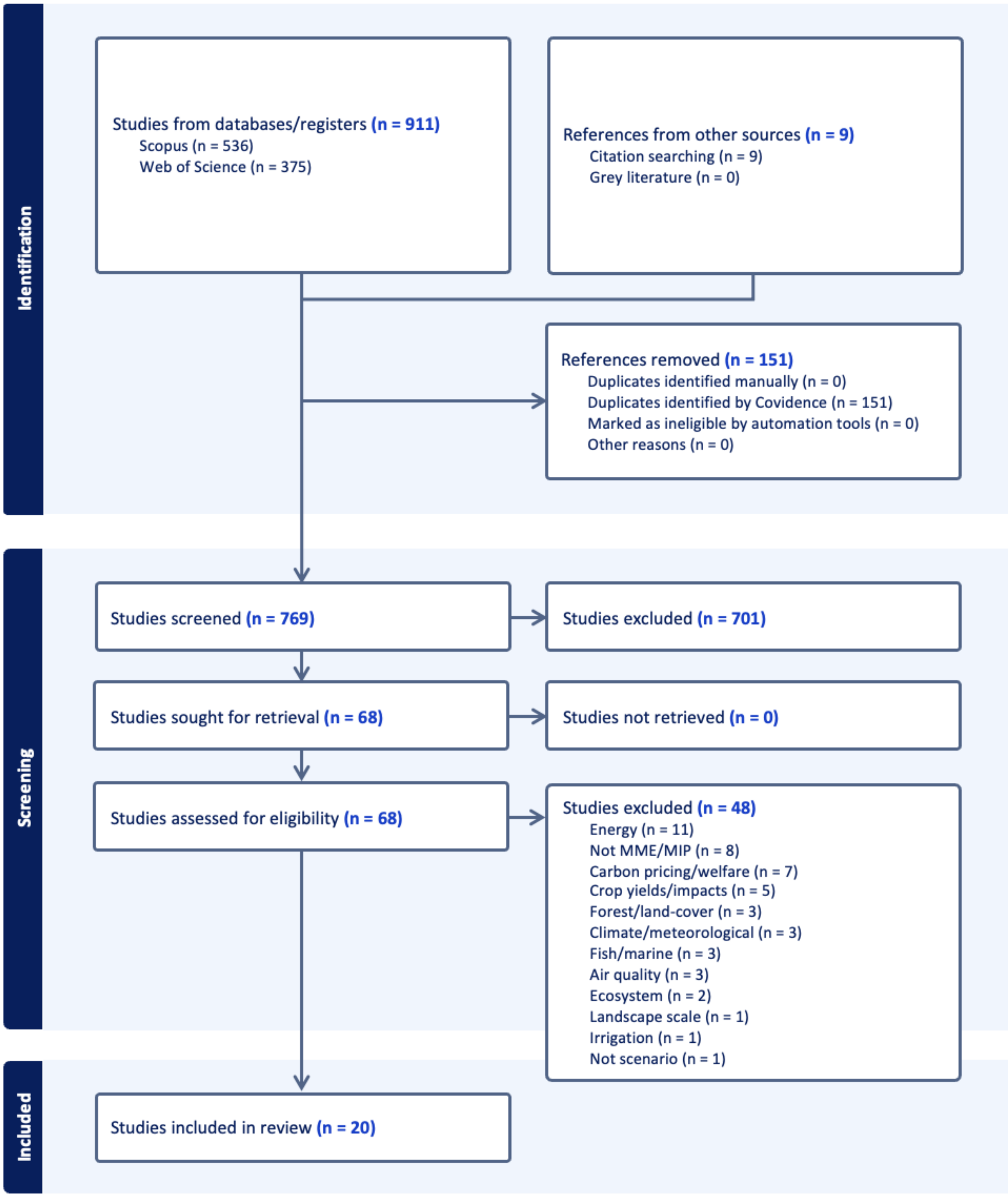

Supplementary Figure 2: *PRISMA summary of multi-model ensemble (MME) studies review*

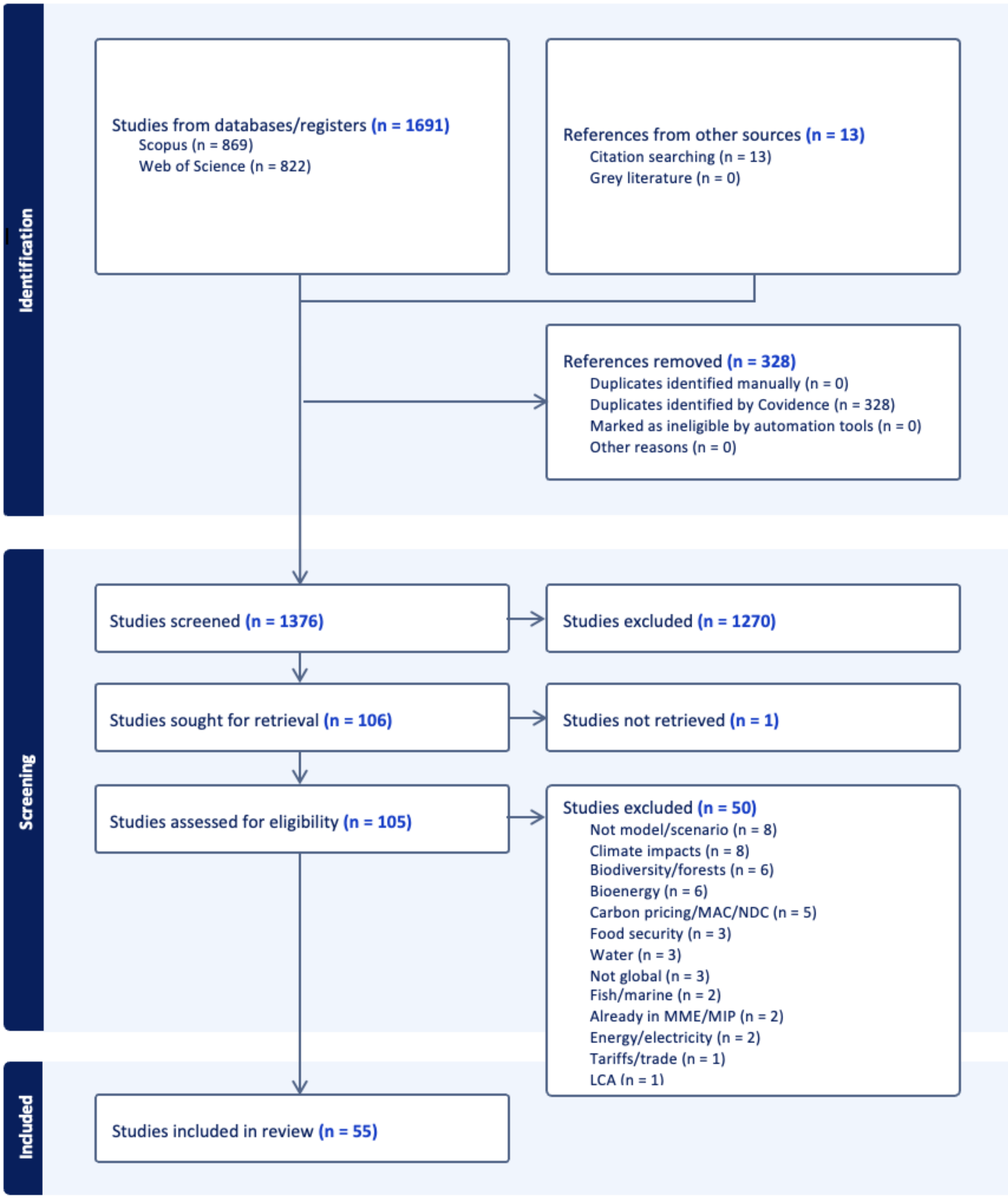

Supplementary Figure 3: *PRISMA summary of single model/non-MME review.*

Supplementary Table 12: Multi-model ensemble studies included in the review. *SE Asia – Southeast Asia. LAM – Latin America.*

Study ID	Title	Scale	Model type(s)	No. and name of models
Frank 2023	A multi-model approach to explore sustainable food and land use pathways for Argentina	Argentina	SD, land-use, conservation	3 (FABLE, Dinamica EGO, NatureMap)
Doelman 2022	Quantifying synergies and trade-offs in the global water-land-food-climate nexus using a multi-model scenario approach	Global	PE/IAM	2 (MAgPIE, IMAGE)
Fujimori 2022	Land-based climate change mitigation measures can affect agricultural markets and food security	Global	PE/CGE	6 (AIM/HUB, FARM, GCAM, CAPRI, GLOBIOM, MAGNET-IMAGE)
van Zeist 2020	Are scenario projections overly optimistic about future yield progress?	Global	PE/CGE	6 (IMAGE-MAGNET, GLOBIOM, AIM/Hub, GCAM, MAgPIE, IMPACT)
Fitton 2019	The vulnerabilities of agricultural land and food production to future water scarcity	Global	PE/CGE/IAM, rules-based, land-use	8 (AIM, FARM, MAGNET, GCAM, GLOBIOM, IMAGE, Falafel, Bajzelj)
Frank 2019	Agricultural non-CO2 emission reduction potential in the context of the 1.5C target	Global	PE/CGE/IAM	4 (CAPRI, GLOBIOM, MAGNET, IMAGE)
Fujimori 2019	A multi-model assessment of food security implications of climate change mitigation	Global	PE/CGE/IAM	6 (AIM, MESSAGE-GLOBIOM, REMIND-MAgPIE, IMAGE, WITCH, POLES)
Stehfest 2019	Key determinants of global land-use projections	Global	PE/CGE/IAM	6 (AIM/Hub, GCAM, GLOBIOM, IMAGE-MAGNET, MAgPIE, IMPACT)
Hasegawa 2018	Risk of increased food insecurity under stringent global climate change mitigation policy	Global	PE, CGE, IAM	8 (AIM/CGE, CAPRI, GCAM, GLOBIOM, IMAGE, IMPACT, MAGNET, MAgPIE)
Popp 2017	Land-use futures in the shared socio-economic pathways	Global	PE/CGE/IAM	5 (AIM, GCAM, IMAGE, MESSAGE-GLOBIOM, REMIND/MAgPIE)
Calvin 2016	Agriculture, forestry, and other land-use emissions in Latin America	LAM	PE/CGE	4 (ADAGE, EPPA, GCAM, TIAM-WORLD (with MAgPIE))
Mason-D'Croz 2016	Multi-factor, multi-state, multi-model scenarios: Exploring food and climate futures for Southeast Asia	SE Asia	PE, land-use	3 (GLOBIOM, IMPACT, LandSHIFT)
Prestele 2016	Hotspots of uncertainty in land-use and land-cover change projections: a global-scale model comparison	Global	PE/CGE, land-use	11 (AIM, FARM, GCAM, MAGNET, PLUM, CAPS, CLUMondo, GLOBIOM, IMAGE, LandSHIFT, MAgPIE)
Wiebe 2015	Climate change impacts on agriculture in 2050 under a range of plausible socioeconomic and emissions scenarios	Global	PE/CGE	5 (FARM, ENVISAGE, MAGNET, IMPACT, MAgPIE)
Lotze-Campen 2014	Impacts of increased bioenergy demand on global food markets: An AgMIP economic model intercomparison	Global	PE/CGE	5 (AIM, MAGNET, GCAM, GLOBIOM, MAgPIE)
Nelson 2014	Agriculture and climate change in global scenarios: why don't the models agree	Global	PE/CGE	9 (AIM, ENVISAGE, FARM, GCAM, GLOBIOM, GTEM, IMPACT, MAGNET, MAgPIE)
Nelson 2014	Climate change effects on agriculture: Economic responses to biophysical shocks	Global	PE/CGE	9 (AIM, ENVISAGE, FARM, GCAM, GLOBIOM, GTEM, IMPACT, MAGNET, MAgPIE)
Schmitz 2014	Land-use change trajectories up to 2050: Insights from a global agro-economic model comparison	Global	PE/CGE	10 (AIM, ENVISAGE, EPPA, FARM, GTEM, MAGNET, GCAM, GLOBIOM, IMPACT, MAgPIE)
Valin 2014	The future of food demand: Understanding differences in global economic models	Global	PE/CGE	10 (AIM, ENVISAGE, EPPA, FARM, GTEM, MAGNET, GCAM, GLOBIOM, IMPACT, MAgPIE)
VonLampe 2014	Why do global long-term scenarios for agriculture differ? An overview of the AgMIP global economic model intercomparison	Global	PE/CGE	10 (AIM, ENVISAGE, EPPA, FARM, GTEM, MAGNET, GCAM, GLOBIOM, IMPACT, MAgPIE)

Supplementary Table 13: *Single model/non-MME studies included in the review.*

No.	Study ID	Title	Model type	Model name
1	van Vuuren 2025	Exploring pathways for world development within planetary boundaries	IAM	IMAGE
2	Humpenöder 2024	Food matters: Dietary shifts increase the feasibility of 1.5C pathways in line with the Paris Agreement	IAM	REMIND-MAgPIE
3	Rulli 2024	Meeting the EAT-Lancet healthy diet target while protecting land and water resources	Agro-hydrological	no name
4	Weindl 2024	Food and land system transformations under different societal perspectives on sustainable development	IAM	REMIND-MAgPIE, IMAGE
5	Yang 2024	Assessing environmental impacts of response strategies for sustainable food system transformation	System dynamics-based IAM	FeliX
6	Almaraz 2023	Model-based scenarios for achieving net negative emissions in the food system	PE	IMPACT
7	Forslund 2023	Can healthy diets be achieved worldwide in 2050 without farmland expansion?	Biomass balance/flow	no name
8	Kok 2023	Assessing ambitious nature conservation strategies in a below 2-degree and food-secure world	IAM	IMAGE-GLOBIO
9	Kyle 2023	Assessing Multi-Dimensional Impacts of Achieving Sustainability Goals by Projecting the Sustainable Agriculture Matrix Into the Future	IAM	GCAM
10	Liu 2023	Robust strategies to end global poverty and reduce environmental pressures	System dynamics-based IAM	FeliX
11	Spillias 2023	Reducing global land-use pressures with seaweed farming	PE	GLOBIOM
12	Xia 2023	Potential environmental and nutritional benefits of replacing ruminant meat with forage fish	CGE	AIM/Hub
13	Ball 2022	C-LLAMA 1.0: a traceable model for food, agriculture, and land use	Statistical empirical	C-LLAMA
14	Bodirsky 2022	Integrating degrowth and efficiency perspectives enables an emission-neutral food system by 2100	PE	MAgPIE
15	Henry 2022	Global and regional health and food security under strict conservation scenarios	Land systems	LandSyMM
16	Humpenöder 2022	Projected environmental benefits of replacing beef with microbial protein	PE	MAgPIE
17	Humpenöder 2022	Overcoming global inequality is critical for land-based mitigation in line with the Paris Agreement	PE	MAgPIE
18	Moallemi 2022	Early systems change necessary for catalyzing long-term sustainability in a post-2030 agenda	System dynamics-based IAM	FeliX
19	Rasche 2022	Food versus wildlife: Will biodiversity hotspots benefit from healthier diets?	Land/crop optimization	no name
20	Kalt 2021	Exploring the option space for land system futures at regional to global scales: The diagnostic agro-food, land use and greenhouse gas emission model BioBaM-GHG 2.0	Biomass balance/flow	BioBaM
21	Komarek 2021	Income, consumer preferences, and the future of livestock-derived food demand	PE	IMPACT
22	Molotoks 2021	Impacts of land use, population, and climate change on global food security	Food supply, diet, malnutrition	FEEDME
23	Pal 2021	Coupled social and land use dynamics affect dietary choice and agricultural land-use extent	Coupled social and land-use	no name
24	Springmann 2021	The global and regional costs of healthy and sustainable dietary patterns: a modelling study	PE	IMPACT
25	Folberth 2020	The global cropland-sparing potential of high-yield farming	Global gridded crop	EPIC
26	Gerten 2020	Feeding ten billion people is possible within four terrestrial planetary boundaries	Biosphere model	LPJmL
27	Mora 2020	Exploring the future of land use and food security: A new set of global scenarios	Biomass balance/flow	GlobAgri-AgT

28	Eker 2019	Modelling the drivers of a widespread shift to sustainable diets	System dynamics-based IAM	FeliX
29	Gouel 2019	Nutrition transition and the structure of global food demand	Consumer demand	MAIDADS
30	Mason-D'Croz 2019	Gaps between fruit and vegetable production, demand, and recommended consumption at global and national levels: an integrated modelling study	PE	IMPACT
31	Bertram 2018	Targeted policies can compensate most of the increased sustainability risks in 1.5C mitigation scenarios	IAM	REMIND-MAgPIE
32	Conijn 2018	Can our global food system meet food demand within planetary boundaries?	N and P mass balance	BIOSPACS
33	Delzeit 2018	Global economic-biophysical assessment of midterm scenarios for agricultural markets - Biofuel policies, dietary patterns, cropland expansion, and productivity growth	CGE	DART-BIO
34	Doelman 2018	Exploring SSP land-use dynamics using the IMAGE model: Regional and gridded scenarios of land-use change and land-based climate change mitigation	IAM	IMAGE, MAGNET, MAGICC, FAIR-SimCAP
35	Kok 2018	Pathways for agriculture and forestry to contribute to terrestrial biodiversity conservation: A global scenario-study	IAM	IMAGE, GLOBIO
36	Nelson 2018	Income growth and climate change effects on global nutrition security to mid-century	PE	IMPACT
37	Springmann 2018	Options for keeping the food system within environmental limits	IO	no name
38	Springmann 2018	Health and nutritional aspects of sustainable diet strategies and their association with environmental impacts: a global modelling analysis with country-level detail	IO	no name
39	Bijl 2017	A physically-based model of long-term food demand	Food demand	no name
40	Bryngelsson 2017	How Do Dietary Choices Influence the Energy-System Cost of Stabilizing the Climate?	Food supply and emissions	WOFSUM
41	Roos 2017	Greedy or needy? Land use and climate impacts of food in 2050 under different livestock futures	Biomass balance/flow	no name
42	Weindl 2017	Livestock production and the water challenge of future food supply: Implications of agricultural management and dietary choices	PE	MAgPIE
43	Eitelberg 2016	Demand for biodiversity protection and carbon storage as drivers of global land change scenarios	Land systems	CLUMondo
44	Springmann 2016	Analysis and valuation of the health and climate change co-benefits of dietary change	Global health risk model with emissions accounting	no name
45	van Vuuren 2015	Pathways to achieve a set of ambitious global sustainability objectives by 2050: Explorations using the IMAGE integrated assessment model	IAM	IMAGE-GLOBIO-MAGNET
46	Baldos 2014	Global food security in 2050: The role of agricultural productivity and climate change	PE	SIMPLE
47	Brunelle 2014	The Impact of Globalization on Food and Agriculture: The Case of the Diet Convergence	PE	Nexus Land-Use
48	Gerst 2014	Contours of a Resilient Global Future	IAM	PoleStar
49	Pardey 2014	A Bounds Analysis of World Food Futures: Global Agriculture Through to 2050	Statistical econometric	iAP
50	Springer 2014	Feeding Nine Billion People Sustainably: Conserving Land and Water through Shifting Diets and Changes in Technologies	IO	World Trade Model
51	Stehfest 2013	Options to reduce the environmental effects of livestock production - Comparison of two economic models	PE	IMPACT, LEITAP
52	Valin 2013	Agricultural productivity and greenhouse gas emissions: Trade-offs or synergies between mitigation and food security?	PE	GLOBIOM

53	Popp 2010	Food consumption, diet shifts and associated non-CO2 greenhouse gases from agricultural production	PE	MAgPIE
54	Stehfest 2009	Climate benefits of changing diet	IAM	IMAGE
55	Lotze-Campen 2008	Global food demand, productivity growth, and the scarcity of land and water resources: A spatially explicit mathematical programming approach	PE	MAgPIE

Sensitivity and uncertainty assessment

Individual scenario drivers

Supplementary Figure 4 shows the multi-model ensemble results for the three individual driver scenarios (BAU_PROD, BAU_WAST, BAU_DIET) with the BAU and EL2 scenarios included for comparison. With these individual driver scenarios, we can isolate the different components of the EL2 scenario to analyse and compare their results across key output variables of interest. The BAU_PROD scenario maintains business as usual trends from 2020 to 2050 but BAU trends on agricultural productivity are replaced with the EAT-Lancet improved agricultural productivity (around 10 to 15 percentage point increase on BAU trends – see Supplementary Table 9). The BAU_WAST scenario maintains business as usual trends from 2020 to 2050 but BAU trends on food loss and waste are replaced with a 50% reduction by 2050 on 2020 levels. The BAU_DIET scenario maintains business as usual trends from 2020 to 2050 but BAU trends on diets are replaced with global achievement of the healthy reference intake by 2050 (see Supplementary Table 8).

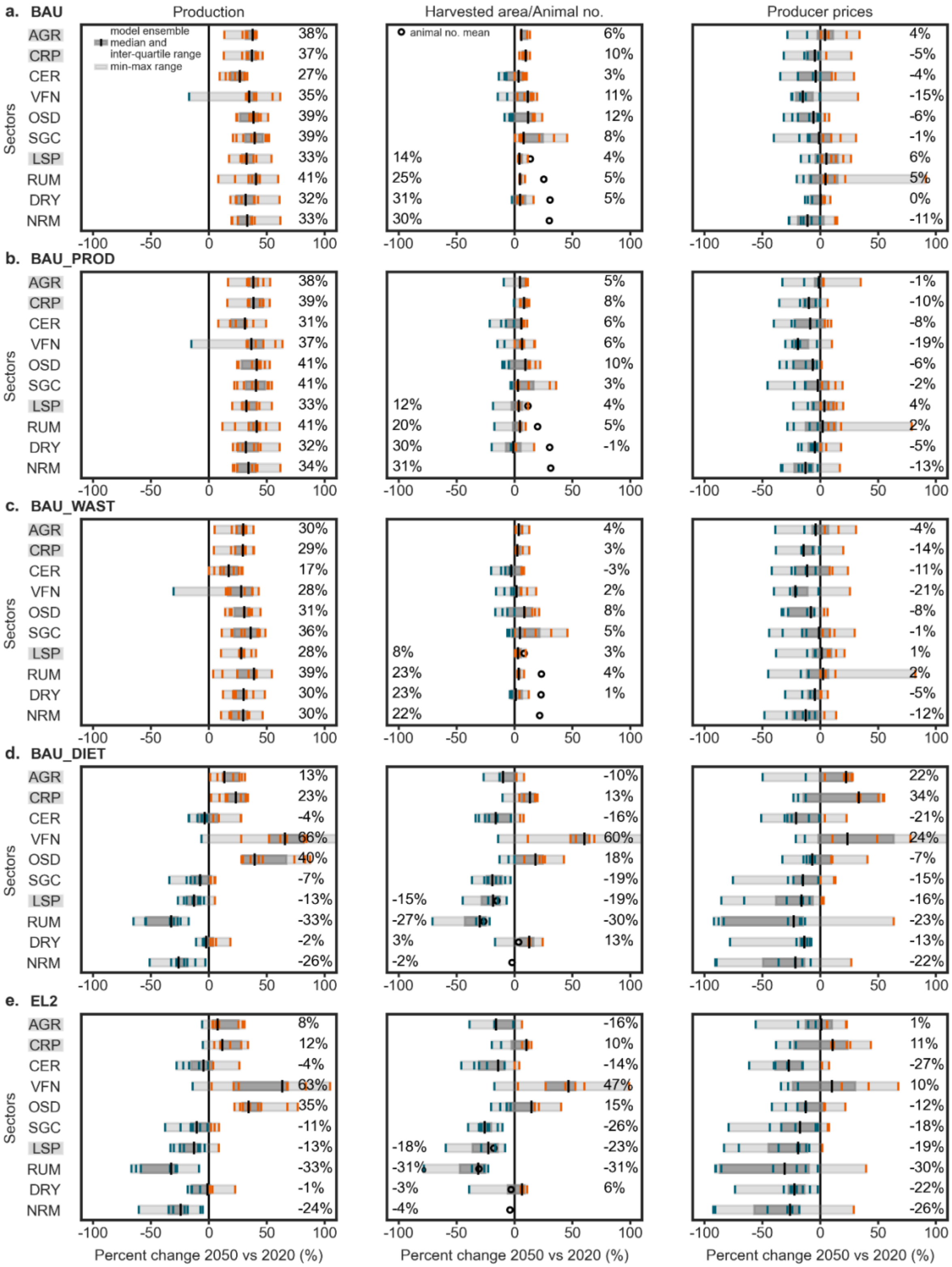

Supplementary Figure 4: Global results from the model ensemble showing percentage change in 2050 from 2020 levels for the BAU scenario (Fig.4a), the BAU_PROD scenario (Fig.4b), the BAU_WAST scenario (Fig.4c), the BAU_DIET scenario (Fig.4d), and the EL2 scenario (Fig.4e). *Results are shown for food group sectors across three food systems dimensions - agricultural production, area (harvested area for crops, pasture area*

and animal numbers for livestock), and producer prices. AGR = all agricultural products (but excluding fisheries and aquaculture), CRP = all crops including cereals (CER) (wheat, rice, coarse grains); vegetables, fruits, nuts, and legumes (VFN); oilseeds (OSD); and sugar crops (SGC) (all shown) and other minor food crops and non-food crops (energy crops and plant-based fibres; not shown separately), LSP = all livestock products include ruminant meat (RUM) (cattle, sheep, and goats), non-ruminants (NRM) (pork, poultry meat and eggs), dairy (DRY), and a category of other animal products (e.g. wool; not shown separately). LSP excludes fisheries and aquaculture. Shaded areas give the range of results (dark grey = inter-quartile range, light grey = min-max range) with individual model results given by teal (percentage decrease) and orange (percentage increase) lines. Black vertical lines indicate median values which are also expressed numerically in each figure (on the right-hand side). Mean (of two reporting models) percentage change in animal numbers is given by unfilled black markers and is represented numerically on the left-hand side.

The BAU and BAU_PROD results show similar median values and inter-quartile ranges. For agricultural production, median values of BAU_PROD are consistently a few percentage points higher than in BAU. This suggests that efficiency gains (through increased productivity) in isolation can lead to increased supply. We also see that changes to producer prices in BAU_PROD are in general lower than in BAU, that is, more efficient production costs less per unit which is reflected in producer prices. Finally, we see that changes to area and animal numbers in BAU_PROD are also in general lower than in BAU – while clear to note that in absolute terms there is still an increase compared to 2020 levels.

The BAU and BAU_WAST scenarios show broadly comparable median values and inter-quartile ranges but median values in BAU_WAST are consistently smaller than in BAU. BAU_WAST median values are also consistently smaller than BAU_PROD. The BAU and BAU_DIET scenarios differ substantially in their median values and inter-quartile ranges. Here we see that the median value for changes to overall agricultural production (AGR) is 15% in BAU_DIET compared to 37% in BAU. Cereals (-1% vs 27%), sugar crops (-7% vs 39%), and livestock products (-14% vs 33%) in particular show substantial changes.

Root-mean square error analysis

Root-mean square error (RMSE) is a common metric for evaluating the performance of how closely predicted data matches observed data. Here we use it to compare how closely the ensemble distribution of results from individual driver scenarios matches that of the BAU and EL2 scenarios respectively. A lower value indicates that an individual driver scenario's results are closer to BAU/EL2 results and a higher value, further away. The average RMSE scores of the model ensemble results for the production variable

(PROD) for BAU_PROD, BAU_WAST, and BAU_DIET scenarios against BAU were 3.7%, 7.9%, and 38.0% respectively. In other words, BAU_WAST and BAU_PROD results for changes to production are quite close to those of the BAU scenario while the BAU_DIET scenario driver is further away. For context, the average RMSE score between the full EL2 scenario and BAU was 37.1%. Average RMSE scores for BAU_PROD, BAU_WAST, and BAU_DIET against the EL2 scenario were 41.5%, 35.8%, and 7.2% respectively (Supplementary Table 14). This indicates that the BAU_PROD and BAU_WAST scenarios had similar and smaller individual effects than the BAU_DIET scenario. This pattern holds for other variables and is visually supported by Supplementary Figure 4 which shows model ensemble results across all five scenarios. Although here it is not possible to assess the interaction effects when combined (see ref. [59]), this provides evidence that the dietary shift component of the EL2 scenario is the most influential individual driver of change.

Specifically, across all 222 scenario-sector-variable triplets, the smallest RMSE scores against BAU in general were those of BAU_PROD and BAU_WAST for the production (PROD) and food demand (FOOD) variables (Supplementary Table 14). That is, these results showed the least amount of difference with BAU. The largest RMSE scores against BAU were those of the BAU_DIET scenario, i.e. the largest difference with BAU results. For comparison with the EL2 scenario, this pattern was reversed, the BAU_DIET had smaller RMSE scores than BAU_PROD and BAU_WAST. This indicates that the BAU_PROD and BAU_WAST scenarios had similar and smaller individual effects than the BAU_DIET scenario. And although here it is not possible to assess the interaction effects when combined, this provides evidence that the dietary shift component of the EL2 scenario is the most influential of the three drivers of change.

Supplementary Table 14: Root-mean square error (RMSE) between individual driver scenarios (BAU_PROD, BAU_WAST, BAU_DIET) and BAU and EL2 for each sector-variable pair. *RMSE is given in native units, i.e. % change in 2050 from 2020 levels. The table is ordered on 'RMSE (BAU)' from smallest to largest.*

No.	Scenario	Sector	Variable	RMSE BAU	RMSE EL2
1	BAU_PROD	AGR	WATR	1.1	9.5
2	BAU_WAST	RUM	AREA	1.2	50.1
3	BAU_PROD	SGC	PROD	1.4	49.5
4	BAU_PROD	DRY	PROD	1.6	38.7

5	BAU_PROD	NRM	PROD	1.6	62.2
6	BAU_PROD	DRY	FOOD	1.6	41.7
7	BAU_PROD	WHT	FOOD	1.8	28.5
8	BAU_PROD	NRM	FOOD	1.8	68.7
9	BAU_PROD	OSD	FOOD	1.9	282.4
10	BAU_PROD	AGR	FOOD	2.1	17.6
11	BAU_PROD	CGR	FOOD	2.1	26.0
12	BAU_PROD	SGC	FOOD	2.1	64.0
13	BAU_PROD	SGC	EMIS	2.2	41.7
14	BAU_PROD	WHT	WATR	2.2	28.5
15	BAU_PROD	VFN	FOOD	2.2	35.0
16	BAU_PROD	OSD	PROD	2.3	10.7
17	BAU_PROD	RUM	PROD	2.3	77.3
18	BAU_WAST	AGR	WATR	2.3	8.2
19	BAU_PROD	RUM	FOOD	2.3	84.1
20	BAU_PROD	WHT	PROD	2.5	31.4
21	BAU_PROD	VFN	PROD	2.5	24.5
22	BAU_PROD	VFN	OTHU	2.6	39.7
23	BAU_PROD	VFN	EMIS	2.7	33.6
24	BAU_PROD	WHT	AREA	2.8	16.3
25	BAU_PROD	OSD	AREA	2.8	7.4
26	BAU_PROD	WHT	EMIS	2.8	29.8
27	BAU_WAST	AGR	AREA	2.9	22.5
28	BAU_PROD	RIC	PROD	3.0	28.1
29	BAU_PROD	RIC	FOOD	3.0	27.2
30	BAU_PROD	AGR	OTHU	3.0	8.5
31	BAU_PROD	WHT	OTHU	3.1	35.1
32	BAU_PROD	DRY	EMIS	3.2	28.5
33	BAU_PROD	OSD	EMIS	3.3	13.4
34	BAU_WAST	DRY	AREA	3.5	18.0
35	BAU_PROD	RIC	AREA	3.6	17.1
36	BAU_WAST	RIC	AREA	3.8	16.4
37	BAU_WAST	CGR	AREA	3.8	19.2
38	BAU_PROD	RIC	EMIS	3.8	22.5
39	BAU_PROD	NRM	EMIS	3.8	55.7
40	BAU_WAST	WHT	WATR	3.9	26.1
41	BAU_PROD	AGR	EMIS	4.2	43.3
42	BAU_PROD	VFN	AREA	4.3	42.3
43	BAU_WAST	OSD	XPRP	4.3	7.6
44	BAU_WAST	OSD	AREA	4.4	7.3
45	BAU_PROD	OSD	XPRP	4.4	8.6
46	BAU_WAST	WHT	AREA	4.6	14.4
47	BAU_PROD	AGR	PROD	4.7	25.9
48	BAU_WAST	RUM	EMIS	4.7	61.8
49	BAU_WAST	OSD	WATR	4.7	24.7

50	BAU_DIET	AGR	FOOD	5.0	13.5
51	BAU_WAST	RIC	XPRP	5.1	18.3
52	BAU_PROD	SGC	XPRP	5.1	17.0
53	BAU_PROD	DRY	XPRP	5.2	25.0
54	BAU_PROD	CGR	EMIS	5.2	30.2
55	BAU_PROD	OSD	WATR	5.2	20.4
56	BAU_PROD	OSD	FRTN	5.2	45.4
57	BAU_PROD	WHT	XPRP	5.3	18.6
58	BAU_WAST	DRY	EMIS	5.3	24.1
59	BAU_WAST	RIC	WATR	5.3	18.0
60	BAU_WAST	AGR	OTHU	5.3	9.0
61	BAU_WAST	SGC	AREA	5.4	37.7
62	BAU_WAST	WHT	XPRP	5.4	18.0
63	BAU_WAST	CGR	XPRP	5.4	15.7
64	BAU_WAST	RUM	FOOD	5.4	79.2
65	BAU_PROD	SGC	FEED	5.5	46.0
66	BAU_WAST	RUM	PROD	5.6	71.1
67	BAU_PROD	RUM	EMIS	5.6	64.3
68	BAU_PROD	SGC	WATR	5.6	38.8
69	BAU_WAST	SGC	XPRP	5.7	17.4
70	BAU_WAST	AGR	EMIS	5.7	38.7
71	BAU_PROD	RIC	WATR	5.7	22.1
72	BAU_PROD	SGC	FRTN	5.7	58.9
73	BAU_WAST	DRY	FOOD	5.7	36.6
74	BAU_DIET	AGR	OTHU	5.7	5.8
75	BAU_PROD	WHT	FRTN	6.0	36.4
76	BAU_PROD	VFN	FRTN	6.0	52.5
77	BAU_WAST	DRY	PROD	6.1	32.2
78	BAU_WAST	NRM	EMIS	6.1	50.8
79	BAU_PROD	SGC	AREA	6.2	35.6
80	BAU_WAST	SGC	WATR	6.2	33.4
81	BAU_PROD	CGR	XPRP	6.3	14.5
82	BAU_WAST	SGC	FOOD	6.3	57.0
83	BAU_PROD	RUM	XPRP	6.4	48.7
84	BAU_WAST	AGR	XPRP	6.5	8.3
85	BAU_WAST	SGC	PROD	6.6	43.0
86	BAU_WAST	CGR	FEED	6.6	49.7
87	BAU_PROD	AGR	AREA	6.7	19.5
88	BAU_DIET	AGR	WATR	6.7	4.0
89	BAU_WAST	VFN	AREA	6.8	44.5
90	BAU_PROD	NRM	XPRP	6.9	32.3
91	BAU_WAST	OSD	PROD	7.0	15.6
92	BAU_PROD	RIC	FRTN	7.0	31.8
93	BAU_WAST	CGR	PROD	7.1	25.3
94	BAU_WAST	DRY	XPRP	7.1	20.7

95	BAU_WAST	NRM	PROD	7.2	54.7
96	BAU_WAST	AGR	FEED	7.2	45.0
97	BAU_WAST	NRM	FOOD	7.3	62.3
98	BAU_WAST	SGC	FEED	7.3	43.3
99	BAU_WAST	VFN	XPRP	7.6	29.7
100	BAU_WAST	AGR	PROD	7.7	16.3
101	BAU_WAST	CGR	EMIS	7.7	25.4
102	BAU_WAST	RIC	FOOD	7.7	17.9
103	BAU_WAST	RIC	EMIS	7.8	17.0
104	BAU_WAST	RIC	PROD	8.0	18.3
105	BAU_PROD	AGR	XPRP	8.0	12.3
106	BAU_WAST	WHT	OTHU	8.2	37.0
107	BAU_WAST	SGC	EMIS	8.4	35.1
108	BAU_PROD	WHT	FEED	8.5	59.2
109	BAU_WAST	NRM	XPRP	8.6	30.7
110	BAU_WAST	RIC	FEED	8.6	41.3
111	BAU_WAST	WHT	FEED	8.7	52.0
112	BAU_DIET	OSD	AREA	8.8	6.9
113	BAU_WAST	OSD	EMIS	9.1	18.3
114	BAU_WAST	CGR	FOOD	9.1	18.1
115	BAU_WAST	OSD	FOOD	9.1	286.2
116	BAU_PROD	VFN	XPRP	9.2	32.7
117	BAU_WAST	AGR	FOOD	9.3	11.4
118	BAU_WAST	VFN	FEED	9.5	65.9
119	BAU_PROD	RIC	XPRP	9.6	16.3
120	BAU_PROD	DRY	AREA	9.7	11.4
121	BAU_PROD	AGR	FRTN	9.7	26.3
122	BAU_WAST	OSD	FEED	9.8	45.2
123	BAU_DIET	DRY	AREA	10.1	13.8
124	BAU_PROD	VFN	WATR	10.2	45.9
125	BAU_WAST	RUM	XPRP	10.3	47.1
126	BAU_WAST	WHT	PROD	10.4	21.2
127	BAU_WAST	CGR	OTHU	10.4	17.1
128	BAU_DIET	RIC	WATR	10.5	13.1
129	BAU_WAST	VFN	OTHU	10.7	45.0
130	BAU_PROD	RUM	AREA	10.8	43.0
131	BAU_WAST	WHT	EMIS	10.9	20.8
132	BAU_WAST	OSD	FRTN	11.0	49.0
133	BAU_PROD	AGR	FEED	11.3	53.5
134	BAU_WAST	CGR	FRTN	11.6	35.5
135	BAU_WAST	RIC	FRTN	11.7	26.1
136	BAU_WAST	WHT	FOOD	11.9	17.9
137	BAU_DIET	CGR	XPRP	12.0	9.7
138	BAU_PROD	CGR	AREA	12.2	25.0
139	BAU_WAST	WHT	FRTN	12.2	28.2

140	BAU_DIET	OSD	XPRP	12.5	10.1
141	BAU_PROD	RIC	FEED	12.6	48.3
142	BAU_WAST	AGR	FRTN	13.2	23.1
143	BAU_WAST	VFN	FOOD	13.2	42.9
144	BAU_DIET	WHT	AREA	13.8	6.1
145	BAU_WAST	VFN	PROD	14.0	33.3
146	BAU_DIET	AGR	XPRP	14.0	14.5
147	BAU_WAST	SGC	FRTN	14.1	49.4
148	BAU_DIET	RIC	XPRP	14.2	10.7
149	BAU_PROD	VFN	FEED	14.2	80.2
150	BAU_DIET	CGR	OTHU	14.3	8.7
151	BAU_PROD	OSD	FEED	14.4	60.3
152	BAU_PROD	CGR	PROD	14.6	39.1
153	BAU_WAST	VFN	WATR	14.7	50.6
154	BAU_DIET	RIC	AREA	14.9	5.8
155	BAU_PROD	CGR	WATR	15.4	43.4
156	BAU_DIET	RIC	EMIS	15.5	9.1
157	BAU_DIET	WHT	XPRP	15.6	7.6
158	BAU_DIET	SGC	XPRP	15.6	7.3
159	BAU_WAST	VFN	EMIS	15.7	44.1
160	BAU_PROD	CGR	OTHU	16.7	8.3
161	BAU_DIET	AGR	PROD	17.7	6.3
162	BAU_WAST	CGR	WATR	17.7	19.0
163	BAU_DIET	AGR	AREA	18.6	6.6
164	BAU_DIET	OSD	PROD	19.7	11.3
165	BAU_DIET	AGR	FRTN	19.7	17.1
166	BAU_DIET	CGR	FOOD	20.0	10.8
167	BAU_DIET	WHT	FOOD	20.3	10.8
168	BAU_DIET	RIC	FOOD	20.4	8.4
169	BAU_DIET	RIC	PROD	20.8	6.6
170	BAU_PROD	CGR	FEED	20.9	64.9
171	BAU_DIET	WHT	EMIS	21.4	10.7
172	BAU_DIET	RIC	FRTN	22.0	16.1
173	BAU_DIET	CGR	AREA	22.3	5.8
174	BAU_WAST	VFN	FRTN	22.8	63.1
175	BAU_PROD	CGR	FRTN	23.0	57.3
176	BAU_DIET	DRY	EMIS	23.3	8.1
177	BAU_DIET	WHT	PROD	24.6	7.7
178	BAU_DIET	OSD	EMIS	25.5	17.5
179	BAU_DIET	CGR	EMIS	25.7	7.7
180	BAU_DIET	WHT	WATR	25.8	6.7
181	BAU_DIET	DRY	XPRP	25.9	7.6
182	BAU_DIET	WHT	FRTN	26.2	13.7
183	BAU_DIET	OSD	WATR	28.0	7.6
184	BAU_DIET	CGR	PROD	30.5	4.8

185	BAU_DIET	VFN	PROD	33.6	20.4
186	BAU_DIET	DRY	PROD	34.0	5.8
187	BAU_DIET	SGC	WATR	34.0	5.2
188	BAU_DIET	NRM	XPRP	34.3	5.6
189	BAU_DIET	WHT	OTHU	34.8	6.5
190	BAU_DIET	CGR	WATR	34.9	6.6
191	BAU_DIET	SGC	AREA	35.2	6.5
192	BAU_DIET	CGR	FRTN	36.4	11.2
193	BAU_DIET	DRY	FOOD	36.9	7.1
194	BAU_DIET	SGC	EMIS	37.1	6.1
195	BAU_DIET	AGR	EMIS	39.0	7.3
196	BAU_DIET	VFN	OTHU	42.0	11.8
197	BAU_DIET	VFN	FOOD	43.6	24.3
198	BAU_DIET	SGC	FEED	45.1	9.4
199	BAU_DIET	RIC	FEED	45.3	11.5
200	BAU_DIET	SGC	PROD	46.5	3.1
201	BAU_DIET	RUM	AREA	47.4	4.2
202	BAU_DIET	AGR	FEED	50.0	11.1
203	BAU_DIET	RUM	XPRP	50.6	10.6
204	BAU_DIET	VFN	EMIS	51.5	28.0
205	BAU_DIET	NRM	EMIS	53.6	5.6
206	BAU_DIET	SGC	FRTN	53.8	11.9
207	BAU_DIET	VFN	AREA	54.3	15.7
208	BAU_DIET	VFN	WATR	54.6	16.3
209	BAU_DIET	OSD	FRTN	54.9	17.5
210	BAU_DIET	OSD	FEED	55.1	10.9
211	BAU_DIET	WHT	FEED	55.2	16.8
212	BAU_DIET	CGR	FEED	56.8	12.3
213	BAU_DIET	VFN	XPRP	57.8	35.1
214	BAU_DIET	NRM	PROD	58.7	4.4
215	BAU_DIET	SGC	FOOD	60.1	4.8
216	BAU_DIET	RUM	EMIS	62.5	4.9
217	BAU_DIET	NRM	FOOD	64.8	4.6
218	BAU_DIET	VFN	FEED	72.9	8.5
219	BAU_DIET	RUM	PROD	74.8	4.5
220	BAU_DIET	VFN	FRTN	77.2	34.0
221	BAU_DIET	RUM	FOOD	80.6	3.4
222	BAU_DIET	OSD	FOOD	510.0	229.4

Inter-quartile range analysis

While RMSE analysis provides insight to the relative importance of the individual driver components (diet shift, improved productivity, reduced FLW) of the full EL2 scenario, to examine the uncertainty range across model ensemble results, we look at the inter-

quartile range (IQR). Supplementary Table 15 shows the mean IQR of multi-model ensemble results for different scenarios, sectors, and variables both for 2050 results relative to the BAU scenario in 2050 and for 2050 results relative to 2020 levels.

Supplementary Table 15: *Mean inter-quartile range (IQR) of multi-model ensemble results for different scenarios, sectors, and variables both for 2050 results relative to the BAU scenario in 2050 (IQR – change vs BAU 2050 (%)) and for 2050 results relative to 2020 levels (IQR – change vs 2020 (%)).*

	**Average IQR**	**IQR - change vs BAU 2050 (%)**	**IQR - change vs 2020 (%)**
**Scenario**			
BAU	19.1	-	19.1
BAU_WAST	12.4	3.6	21.1
BAU_PROD	12.5	5.0	20.1
BAU_DIET	23.6	18.0	29.3
EL2	27.7	21.3	34.1
**Sector**			
DRY	12.2	9.4	14.5
RIC	16.2	9.6	21.5
SGC	17.0	9.1	23.2
CGR	19.0	12.7	24.1
OSD	19.9	11.4	26.7
WHT	22.0	14.2	28.2
NRM	27.8	12.3	40.1
VFN	28.0	19.7	34.6
RUM	30.7	16.5	42.1
**Variable**			
AREA	12.6	10.8	14.1
PROD	13.5	10.1	16.1
WATR	14.0	8.5	18.5
EMIS	14.3	9.3	18.3
FOOD	17.7	13.8	20.7
FRTN	19.8	10.1	27.6
XPRP	20.3	15.7	24.0
FEED	22.4	13.0	29.9
OTHU	35.1	14.4	51.7
**Overall**	19.1	12.0	24.7

Overall, multi-model ensemble results for 2050 for scenarios relative to BAU had a smaller IQR (12.0%) than results for 2050 for scenarios relative to 2020 levels (24.7%). That is, on average there was less uncertainty in model results that compared two

scenarios at a future date (2050) than model results that compared future scenarios versus the current (2020).

The EL2 scenario had an IQR of 34.1% for relative change between 2050 and 2020 compared to the BAU reference scenario 19.1%. The smaller model IQR for BAU is expected given the reference scenario was constructed based on continuation of current trends. When comparing IQR from results in 2050 relative to the BAU scenario we see that BAU_WAST and BAU_PROD have relatively small IQRs on average (3.6% and 5.0% respectively) whereas BAU_DIET and EL2 have much larger ranges (18.0% and 21.3% respectively). This indicates that the dietary shift component has greater model uncertainty than ensemble results for modelling improved agricultural productivity and reduced food loss and waste.

For agricultural sectors, the smallest IQR is for dairy (DRY), rice (RIC) and sugar crops (SGC) (9.4%, 9.6%, 9.1%) and largest is for vegetables, fruits, nuts, and legumes (VFN), and ruminant meat (RUM) (19.7% and 16.5% respectively). These differences across sectors likely reflect two main factors. Firstly, these latter two sectors change most substantially with shifts to healthy diets (for example increases to VFN and decreases to RUM). Secondly, sectors that are represented largely as single or bulk commodities (e.g. sugar crops, grains, oilseeds) have less heterogeneity than aggregate sectors, e.g. VFN, which is composed of a diverse group of agricultural products.

For variables, area (AREA) and production (PROD) have smaller overall average IQRs (10.8% and 10.1% respectively) than producer prices (XPRP), animal feed (FEED) and other use (OTHU) (20.3%, 22.4%, 35.1% respectively). The latter consists of demand for agricultural products for industrial use and the bioeconomy (e.g. biofuel, bioenergy). Area and production are the two key metrics that the multi-model ensemble is able to report on with well-understood and characterised processes and dynamics. The larger IQR for producer prices is likely somewhat a reflection of different model assumptions on price elasticities and assumptions on the speed, scale and sensitivity of producers switching agricultural commodity production. A likely primary reason why ‘other use’ (OTHU) has

the largest IQR is that the scenarios purposely did not provide a storyline for this variable. I.e. food and feed demand were influenced substantially by the shifts toward healthy diets, whereas there was no comparable factor from a scenario narrative standpoint for non-food/feed other demand.

To explore this from a bioenergy perspective, we give results for land-use changes from dedicated energy crops in the different scenarios versus BAU projections (Supplementary Figure 9). The five models that report on this sector show that, in general but with a range of uncertainty, an EL2 scenario leads to a relative increase in energy crop land use versus BAU by 2050. The different models and modelling frameworks in this study have somewhat different approaches to land-use. But in general, land-use is determined by land market prices, demand, and any national policies that constrain or incentivise different types of land use. For example, it is instructive from Supplementary Figure 9 that the USA, a region that sees one of the largest relative reductions in agricultural production in an EL2 versus BAU scenario, has one of the biggest relative increases in land for energy crops (+22% versus BAU).

VFN sector scatter plots

Supplementary Figure 5 shows scatterplots of changes in VFN (vegetable, fruit, nut, legume) sector producer price results versus changes in production, harvested area, and yields. Results are given for all models and regions in the EL2 scenario. A linear trends line is fitted for each pair of variables. The scatterplots show an inverse relationship between producer prices and the three other variables. That is, with increasing percentage change to production, area, and yield in EL2 versus 2020 levels, the change in producer prices falls.

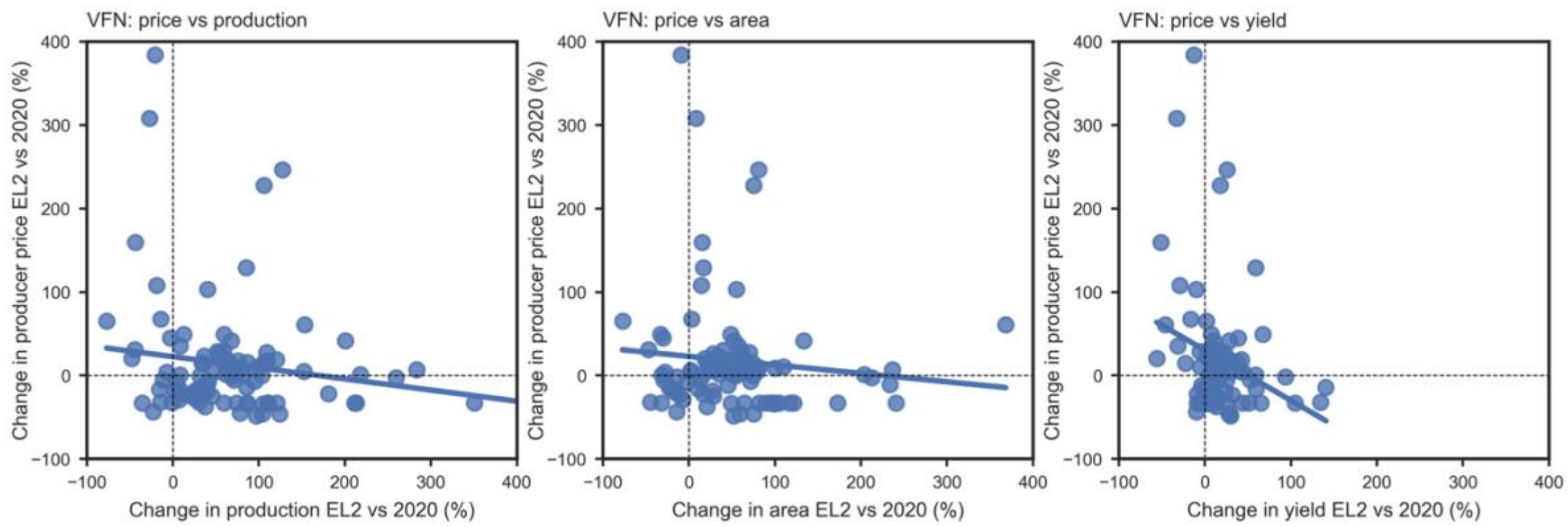

Supplementary Figure 5: Scatterplots of changes in VFN (vegetable, fruit, nut, legume) sector producer price results versus changes in production, harvested area, and yields. *Results are given for all models and regions in the EL2 scenario. A linear trends line is fitted for each pair of variables.*

Food loss and waste sensitivity

Supplementary Figure 6 shows the results of the food loss and waste (FLW) sensitivity for BAU_WAST and EL2 scenarios for 10 sectors and 3 variables (production, harvested

area, producer prices). The sensitivity analysis varied the reduction in FLW from 50% to 25% and 75%.

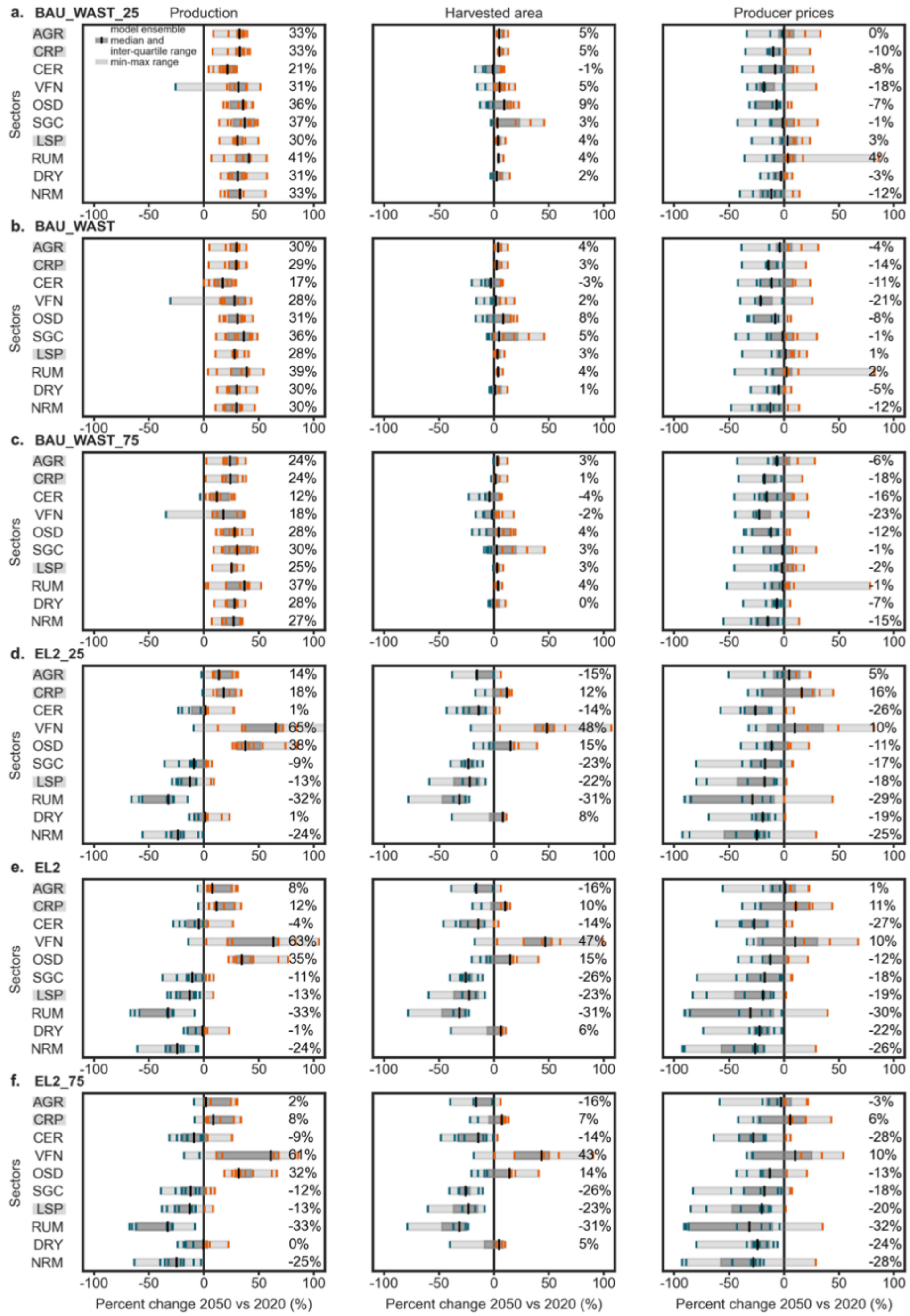

.

Supplementary Figure 6: Food loss and waste (FLW) sensitivity for BAU_WAST and EL2 scenarios for 10 sectors and 3 variables (production, harvested area, producer prices). *a. shows BAU_WAST with FLW reduced by 25%, b. shows BAU_WAST with FLW reduced by 50%, c. shows BAU_WAST with FLW reduced by 75%, d. shows EL2 with FLW reduced by 25%, e. shows EL2 with FLW reduced by 50%, f. shows EL2 with FLW reduced by 75%.*

RMSE analysis FLW sensitivity

Supplementary Table 16 summaries RMSE analysis of the FLW sensitivity scenarios for different sectors and variables. Comparison is made between the 25% and 75% reduction scenarios (BAU_WAST_25/75 and EL2_25/75) against the BAU_WAST and EL2 scenarios (which have 50% FLW reduction). The 25% and 75% FLW scenarios are also compared with each other. We see that in general, the 25% and 75% reduction scenarios have small RMSE scores (2.9% difference on average) when compared to their 50% equivalents. However, when the 25% and 75% scenarios are compared to each other, we obtain higher RMSE scores on average (5.5%).

Supplementary Table 16: Root mean squared error (RMSE) between different multi-model ensemble results of different FLW sensitivity scenarios. *Units are in the native units of the results, i.e. relative change (%). Results are given for four scenarios (BAU_WAST_25 and BAU_WAST_75 vs BAU_WAST and EL2_25 and EL2_75 vs EL2. I.e. the 25% and 75% FLW sensitivities are compared with the core 50% FLW scenarios. And then the 25%/75% scenarios are compared with each other (last two columns). 10 sectors and three variables, ordered from smallest to largest RMSE scores.*

		RMSE vs BAU_WAST (%)		RMSE vs EL2 (%)		RMSE vs BAU_WAST_ 25/75 (%)	RMSE vs EL2_25/75 (%)
**Sector**	**Variable**	**BAU_WAST_25**	**BAU_WAST_75**	**EL2_25**	**EL2_75**		
CGR	XPRP	0.7	0.5	0.3	0.3	1.2	0.6
AGR	XPRP	0.9	0.8	2.1	2.0	1.7	4.1
DRY	PROD	0.9	0.9	0.7	0.8	1.8	1.5
OSD	PROD	1.2	1.2	2.0	2.0	2.5	3.8
NRM	PROD	1.5	1.4	1.5	1.5	2.8	3.0
VFN	XPRP	1.6	1.7	0.9	0.9	3.2	1.8
CRP	XPRP	1.8	1.6	1.8	2.2	3.0	3.9
LSP	PROD	1.8	1.8	2.9	2.9	3.6	5.5
WHT	XPRP	1.8	1.6	1.9	1.6	3.3	3.2
SGC	PROD	1.9	1.8	2.1	1.9	3.6	3.7
RUM	PROD	2.1	2.4	3.0	2.2	4.2	4.8
WHT	AREA	2.2	2.3	2.5	2.3	4.2	4.8
DRY	XPRP	2.3	1.8	2.0	1.9	4.1	3.9
LSP	XPRP	2.3	1.8	8.4	6.8	4.0	15.0
RUM	XPRP	2.3	2.7	2.3	2.3	4.6	4.3
SGC	XPRP	2.3	1.9	2.0	2.2	4.1	4.1
CRP	AREA	2.5	2.5	2.9	4.0	4.9	6.6
NRM	XPRP	2.8	3.1	2.4	1.8	5.8	3.2
RIC	AREA	2.8	2.5	3.4	2.8	5.3	6.1
AGR	AREA	3.0	2.8	3.1	2.9	5.8	6.0
RIC	XPRP	3.1	1.5	5.0	4.4	4.3	9.1

LSP	AREA	3.2	2.7	8.9	8.2	5.6	16.8
VFN	AREA	3.4	2.9	3.6	3.5	6.2	6.9
OSD	XPRP	3.5	3.1	4.3	3.8	6.6	8.0
AGR	PROD	3.7	4.2	3.8	4.3	7.4	7.6
CGR	AREA	3.7	3.4	4.1	3.6	7.0	7.5
RIC	PROD	3.7	3.8	6.1	6.0	6.9	11.6
VFN	PROD	3.7	4.0	4.4	4.6	7.7	9.0
WHT	PROD	3.7	3.6	3.3	3.9	6.8	5.8
CGR	PROD	3.8	3.4	3.1	3.4	6.5	6.5
SGC	AREA	3.8	3.5	4.5	4.4	7.0	8.3
CRP	PROD	4.0	3.9	4.5	4.8	7.7	8.9
OSD	AREA	4.0	3.3	4.2	3.6	7.1	7.6
DRY	AREA	4.1	2.6	2.6	1.4	5.0	3.7
RUM	AREA	4.8	4.4	2.1	2.6	8.7	4.7
NRM	AREA	5.3	2.6	2.7	2.3	6.3	4.0

Supplementary Table 17 reports individual model results for the change in total agricultural production by 2050 for the three FLW scenarios versus the BAU scenario. All models showed larger negative percentage changes in production as reduction in FLW goes from 25% to 50% and 75% (left to right) versus BAU by 2050, but the magnitude of the change varied. ENVISAGE, GLOBIOM and MAgPIE showing the largest changes in production relative to BAU while IMAGE, IMPACT and MAGNET showed the smallest.

The median model values for sector production, area and producer prices showed the expected behaviour of larger relative declines (of a few percentage points) as FLW reduction ambition increased. For total agricultural production, an EL2 scenario with 25% reduction in FLW sees a median increase of 14% by 2050 on 2020 levels. In contrast, an EL2 scenario with 75% reduction shows only a 2% median increase on 2020 levels. And so, while dietary shift is the largest individual driver, the level of ambition of reductions in FLW matters.

Supplementary Table 17: *Model results for the relative change in global agricultural production by 2050 for the three food loss and waste scenarios (25% reduction in FLW – BAU_WAST_25, 50% reduction in FLW – BAU_WAST, 75% reduction in FLW – BAU_WAST_75) versus the business-as-usual (BAU) scenario.*

	**Change in agricultural production by 2050 versus BAU (%)**

Model	BAU_WAST_25	BAU_WAST	BAU_WAST_75
AIM	-5.3	-7.1	-8.8
CAPRI	-4.2	-7.3	-9.8
ENVISAGE	-4.6	-8.7	-12.2
GCAM	-2.7	-4.8	-6.7
GLOBIOM	-5.4	-10.0	-14.2
IMAGE	-0.7	-1.3	-1.8
IMPACT	-0.6	-1.2	-1.7
MAGNET	-0.5	-0.9	-1.4
MAgPIE	-2.9	-8.3	-14.8

General remarks on model uncertainty

Our results for agricultural land, GHG emission and production changes are consistent with previous modelling studies[10,49,60–62].There are fewer studies to compare with respect to changes in the economic value of production, but those that do have shown that aggregate world agricultural market prices decline in a future with less meat consumption, higher productivity and reduced FLW[61]. While there is good general agreement between models across the results shown here, uncertainties remain (as described above).

One rationale for employing an ensemble of models is to better capture the possibility space of scenario results, where larger differences in the range of results obtained from the different models indicates areas of greater model uncertainty. We do not consider, though highly important, model parameter uncertainty. A strength of multi-model ensemble analyses such as this is the integration of a diversity of model perspectives. A reflection of this is how multi-model approaches are increasingly being employed to investigate inequality[63], climate policy[64], fisheries[65], biodiversity[66], air quality[67], and irrigation[68].

The uncertainties highlighted above signal future modelling research needs to better characterise food sectors that are more heterogenous with less data readily available to modellers (e.g. vegetables, fruits, nuts, legumes) and to investigate how production not for food or feed use (e.g. industrial, bioeconomy) responds and restructures in the face

of food system transformation. This extends to fisheries and aquaculture which are currently not as well captured by agro-economic models and were not included in this study.

More general explanations for observed differences include model design, parameterization, and representation. The ensemble comprised IAM, partial equilibrium, and CGE models which have different foci of analysis (e.g. biogeochemical cycles, agricultural markets, economy-wide analysis). Models have varying detail and representation of both land and production activities, flexibility in the response and type of land-use change (e.g. protected areas, forestry, biofuels) and spatial constraints on different crop and livestock production. Socio-economically, trade responsiveness differs across models, there are differences in the responsiveness of producers and consumers to commodity price signals, and models vary in the extent to which income changes are captured in consumption responses. Another reason for differences in model results is how model groups interpreted and implemented scenarios. Key scenario dimensions, for example SSP2 projections on population and economic growth, the food group quantities in the healthy reference diet, agricultural productivity, and climate impacts were harmonized across models. However, within the scenario specifications, modelling groups followed individual assumptions, based on a priori knowledge, on business-as-usual trends, endogenous productivity changes, and the dynamic feedbacks in response to reduction in food loss and waste, and transitions to EAT-Lancet healthy diets.

Supplementary results

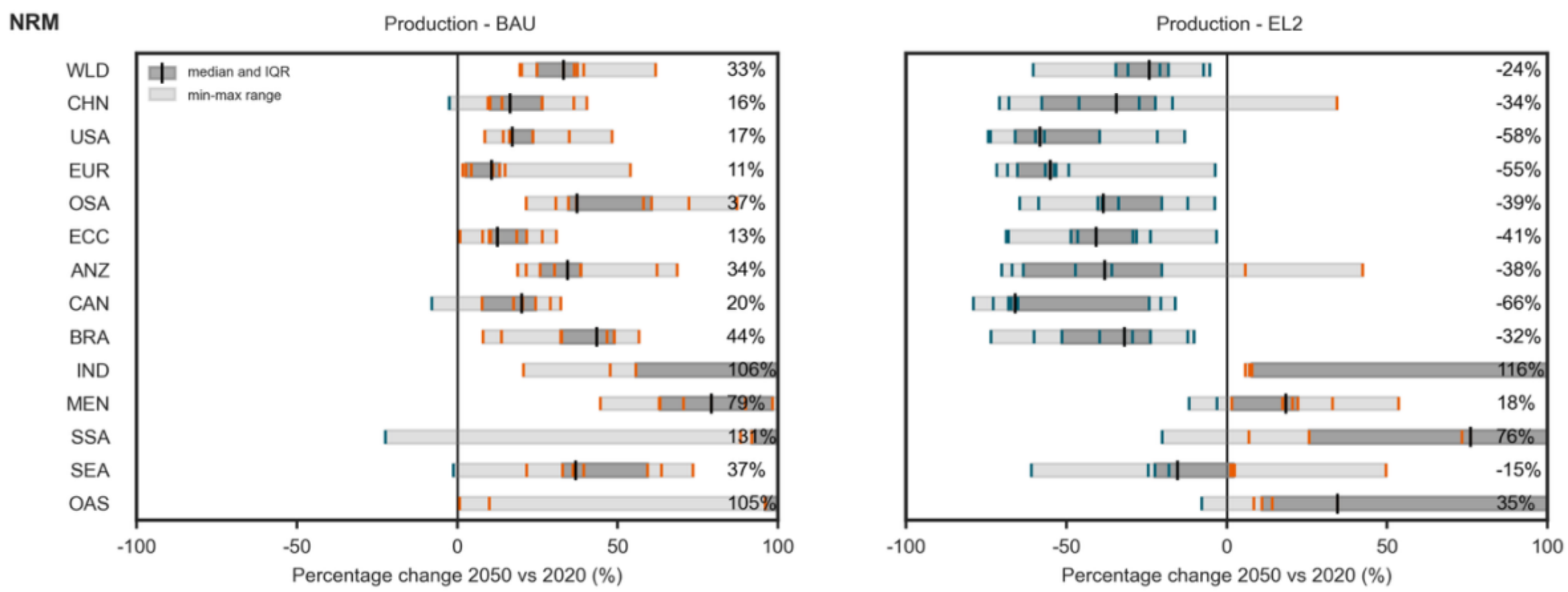
NRM
Production - BAU
Production - EL2
median and IQR
min-max range
WLD
CHN
USA
EUR
OSA
ECC
ANZ
CAN
BRA
IND
MEN
SSA
SEA
OAS
33%
16%
17%
11%
37%
13%
34%
20%
44%
106%
79%
131%
37%
105%
-24%
-34%
-58%
-55%
-39%
-41%
-38%
-66%
-32%
116%
18%
76%
-15%
35%
-100
-50
0
50
100
Percentage change 2050 vs 2020 (%)

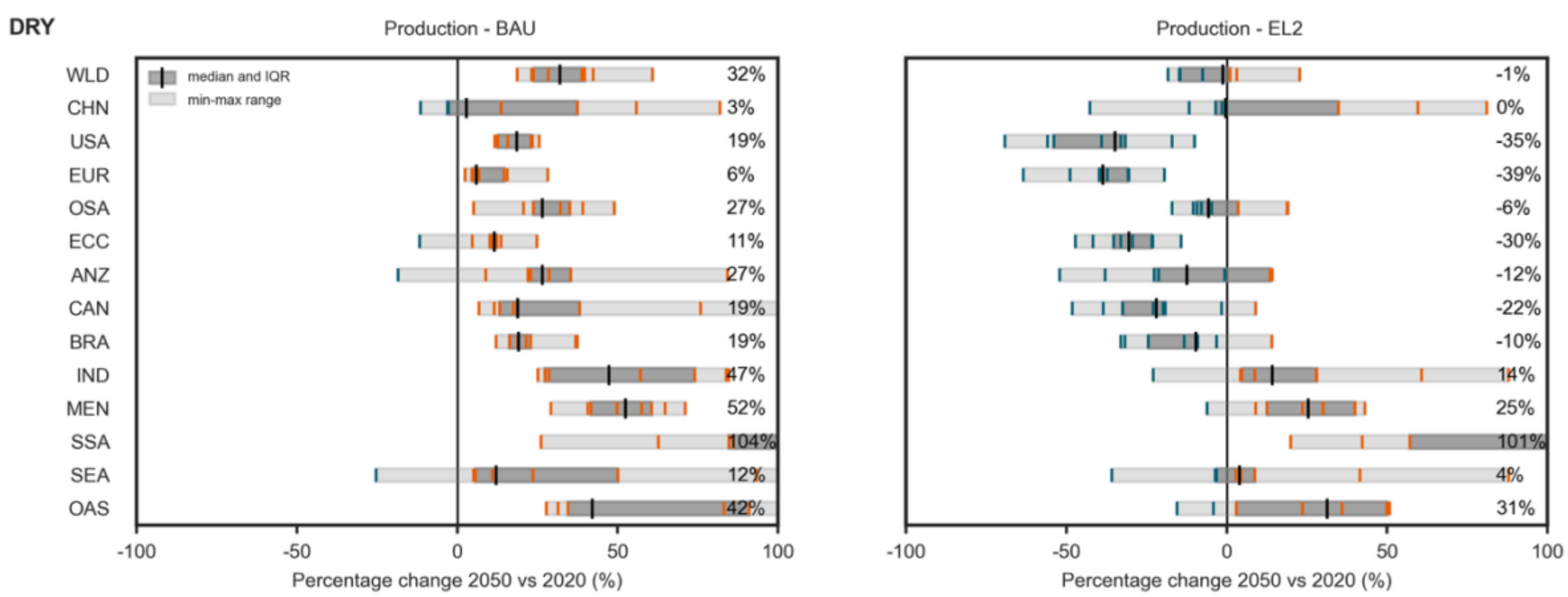
DRY
Production - BAU
Production - EL2
median and IQR
min-max range
WLD
CHN
USA
EUR
OSA
ECC
ANZ
CAN
BRA
IND
MEN
SSA
SEA
OAS
32%
3%
19%
6%
27%
11%
27%
19%
19%
47%
52%
104%
12%
42%
-1%
0%
-35%
-39%
-6%
-30%
-12%
-22%
-10%
14%
25%
101%
4%
31%
-100
-50
0
50
100
Percentage change 2050 vs 2020 (%)

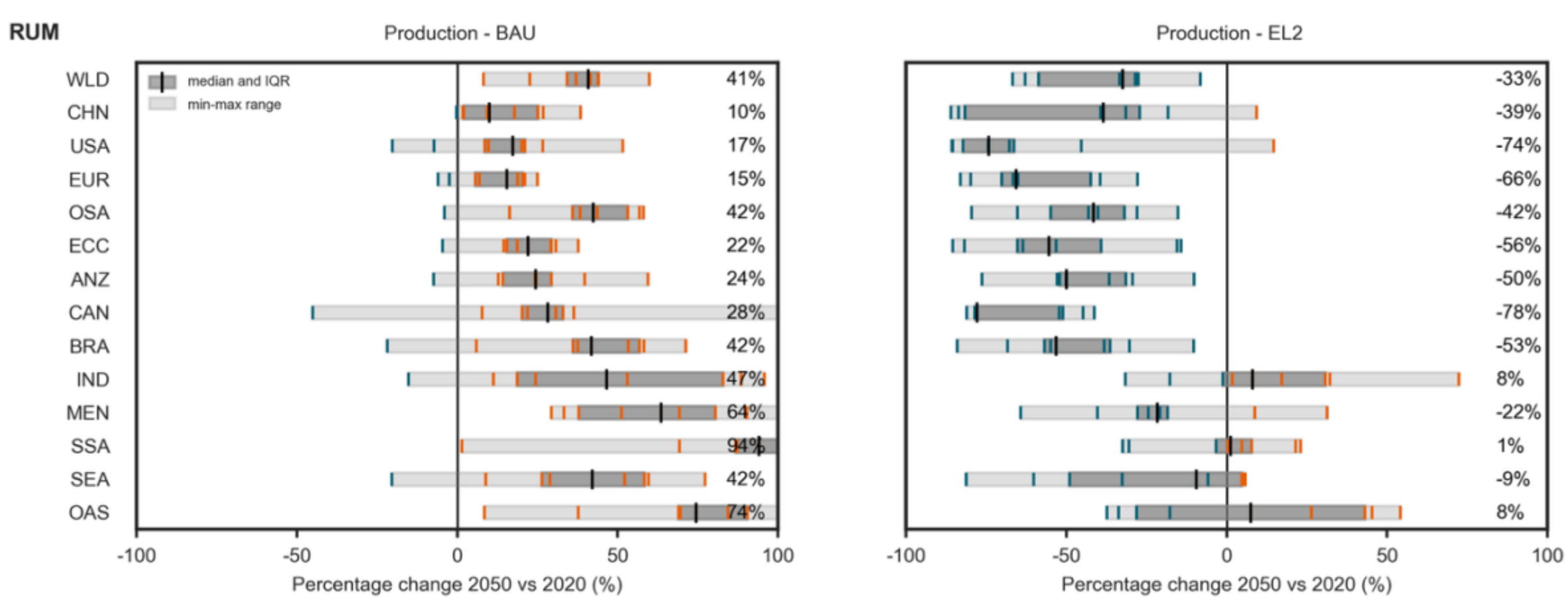
RUM
Production - BAU
Production - EL2
median and IQR
min-max range
WLD
CHN
USA
EUR
OSA
ECC
ANZ
CAN
BRA
IND
MEN
SSA
SEA
OAS
41%
10%
17%
15%
42%
22%
24%
28%
42%
47%
64%
94%
42%
74%
-33%
-39%
-74%
-66%
-42%
-56%
-50%
-78%
-53%
8%
-22%
1%
-9%
8%
-100
-50
0
50
100
Percentage change 2050 vs 2020 (%)

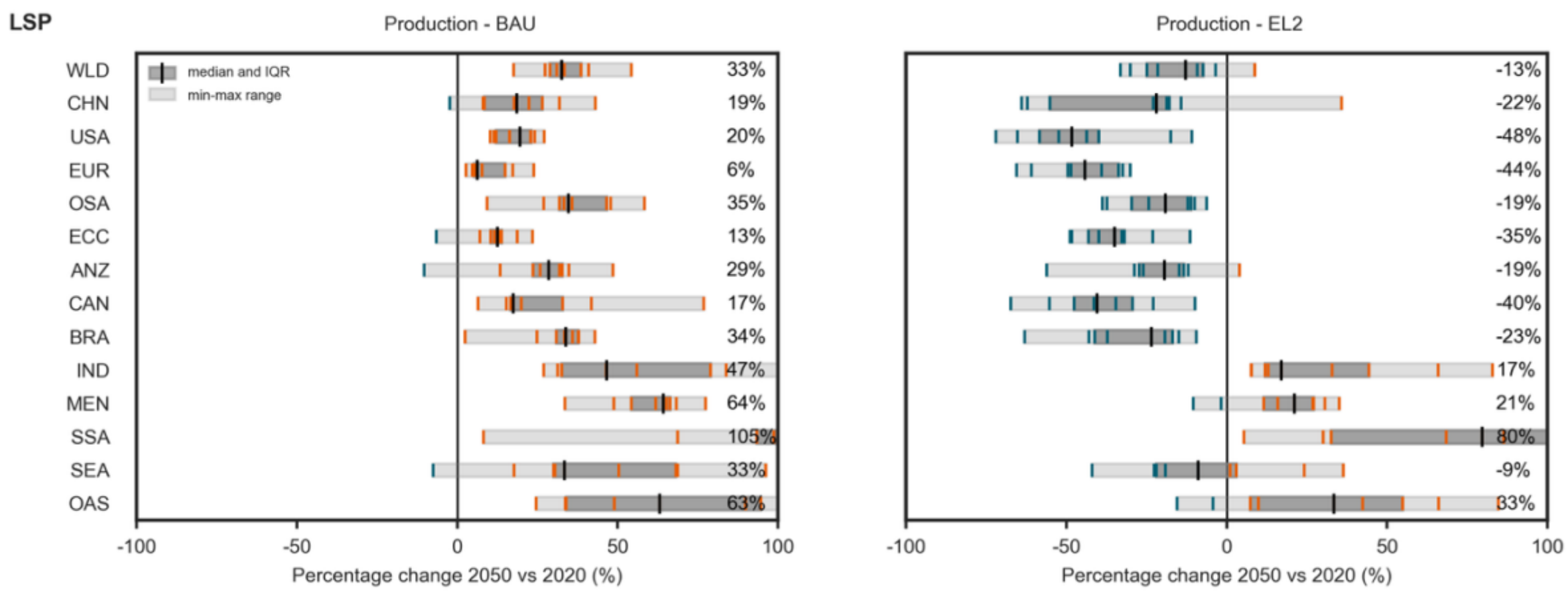
LSP
Production - BAU
Production - EL2
median and IQR
min-max range
WLD
CHN
USA
EUR
OSA
ECC
ANZ
CAN
BRA
IND
MEN
SSA
SEA
OAS
33%
19%
20%
6%
35%
13%
29%
17%
34%
47%
64%
105%
33%
63%
-13%
-22%
-48%
-44%
-19%
-35%
-19%
-40%
-23%
17%
21%
80%
-9%
33%
-100
-50
0
50
100
Percentage change 2050 vs 2020 (%)

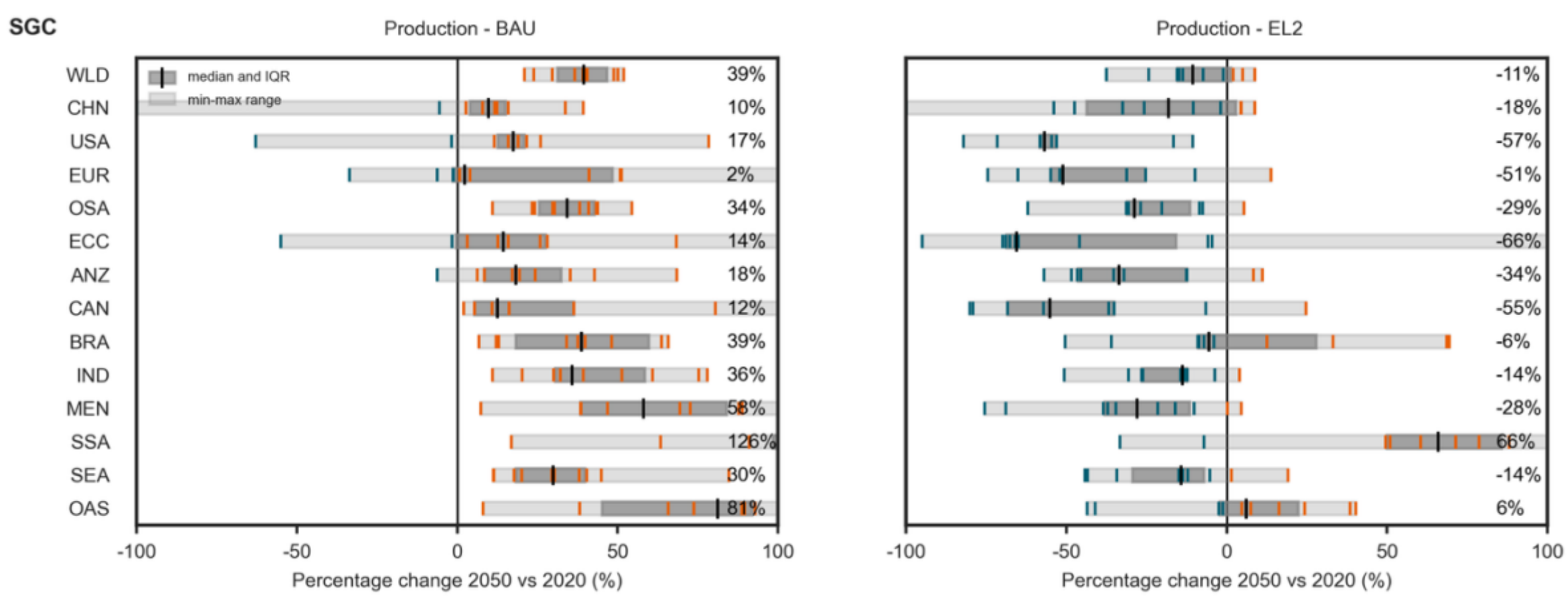
SGC
Production - BAU
Production - EL2
median and IQR
min-max range
WLD
CHN
USA
EUR
OSA
ECC
ANZ
CAN
BRA
IND
MEN
SSA
SEA
OAS
39%
10%
17%
2%
34%
14%
18%
12%
39%
36%
58%
126%
30%
81%
-11%
-18%
-57%
-51%
-29%
-66%
-34%
-55%
-6%
-14%
-28%
66%
-14%
6%
-100
-50
0
50
100
Percentage change 2050 vs 2020 (%)

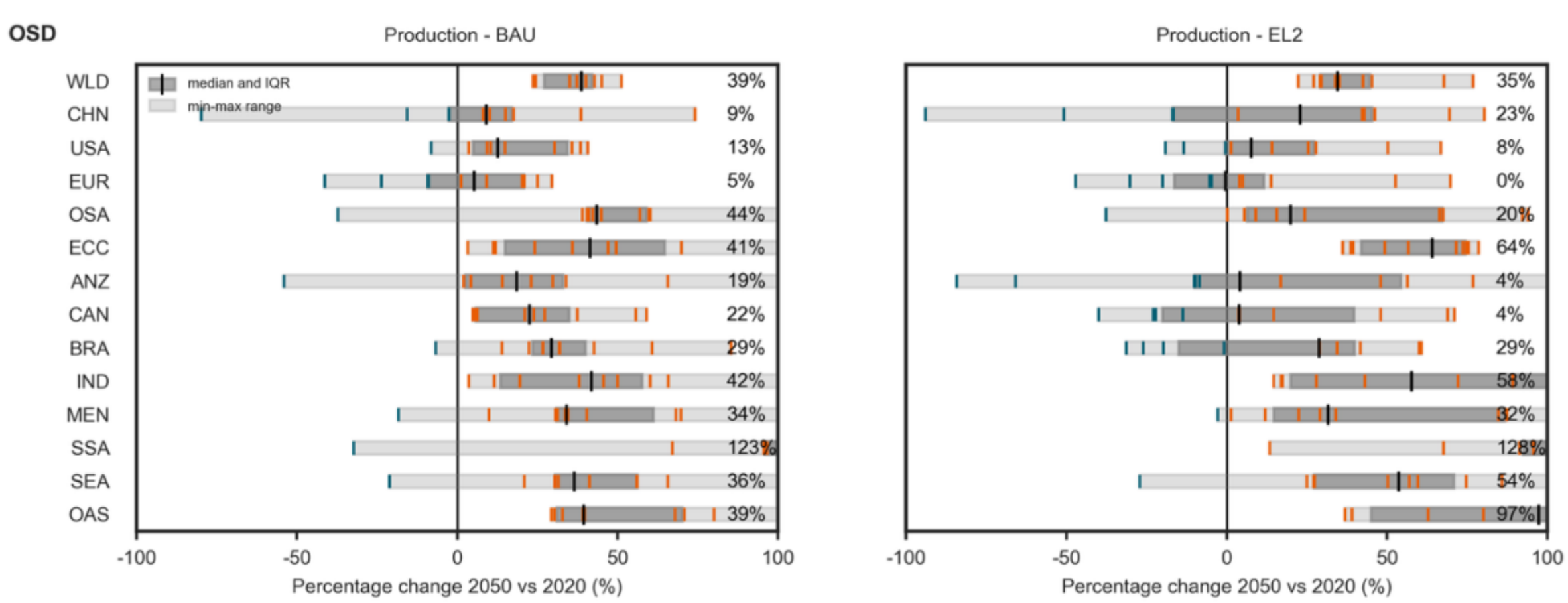
OSD
Production - BAU
Production - EL2
median and IQR
min-max range
WLD
CHN
USA
EUR
OSA
ECC
ANZ
CAN
BRA
IND
MEN
SSA
SEA
OAS
39%
9%
13%
5%
44%
41%
19%
22%
29%
42%
34%
123%
36%
39%
35%
23%
8%
0%
20%
64%
4%
4%
29%
58%
32%
128%
54%
97%
-100
-50
0
50
100
Percentage change 2050 vs 2020 (%)

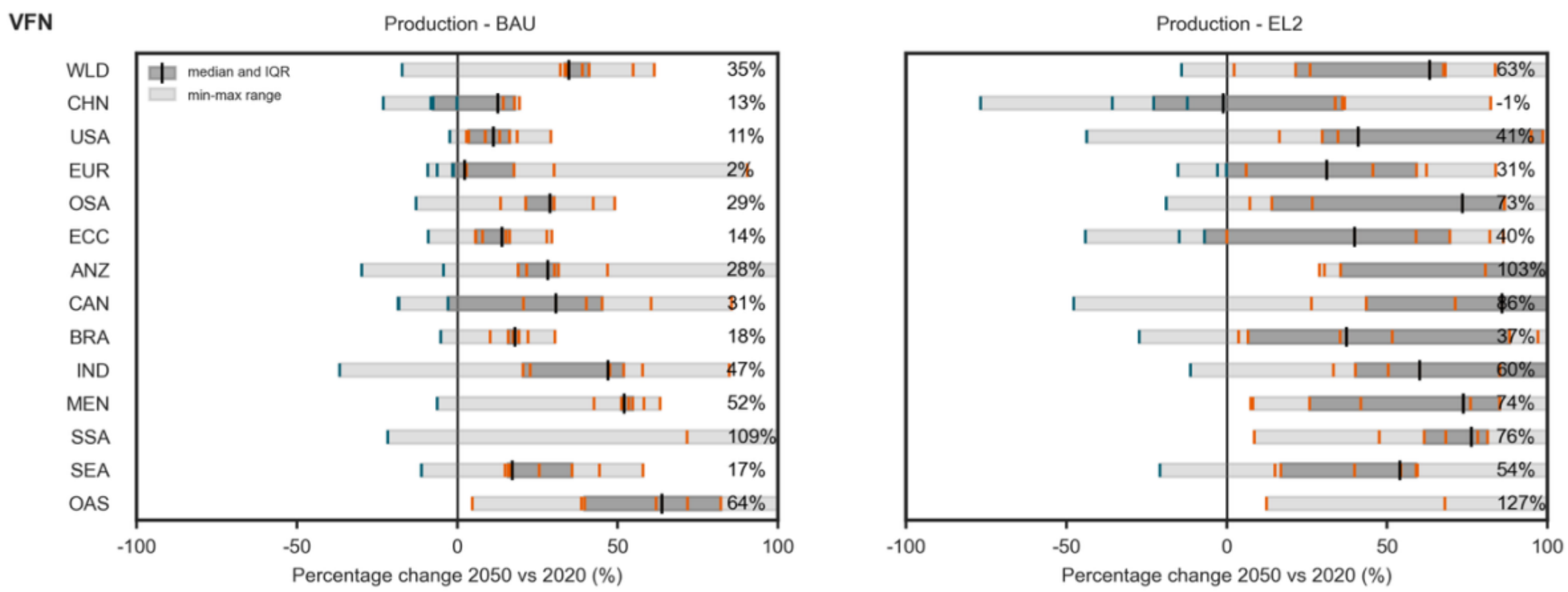
VFN
Production - BAU
Production - EL2
median and IQR
min-max range
WLD CHN USA EUR OSA ECC ANZ CAN BRA IND MEN SSA SEA OAS
35% 13% 11% 2% 29% 14% 28% 31% 18% 47% 52% 109% 17% 64%
63% -1% 41% 31% 73% 40% 103% 86% 37% 60% 74% 76% 54% 127%
Percentage change 2050 vs 2020 (%)

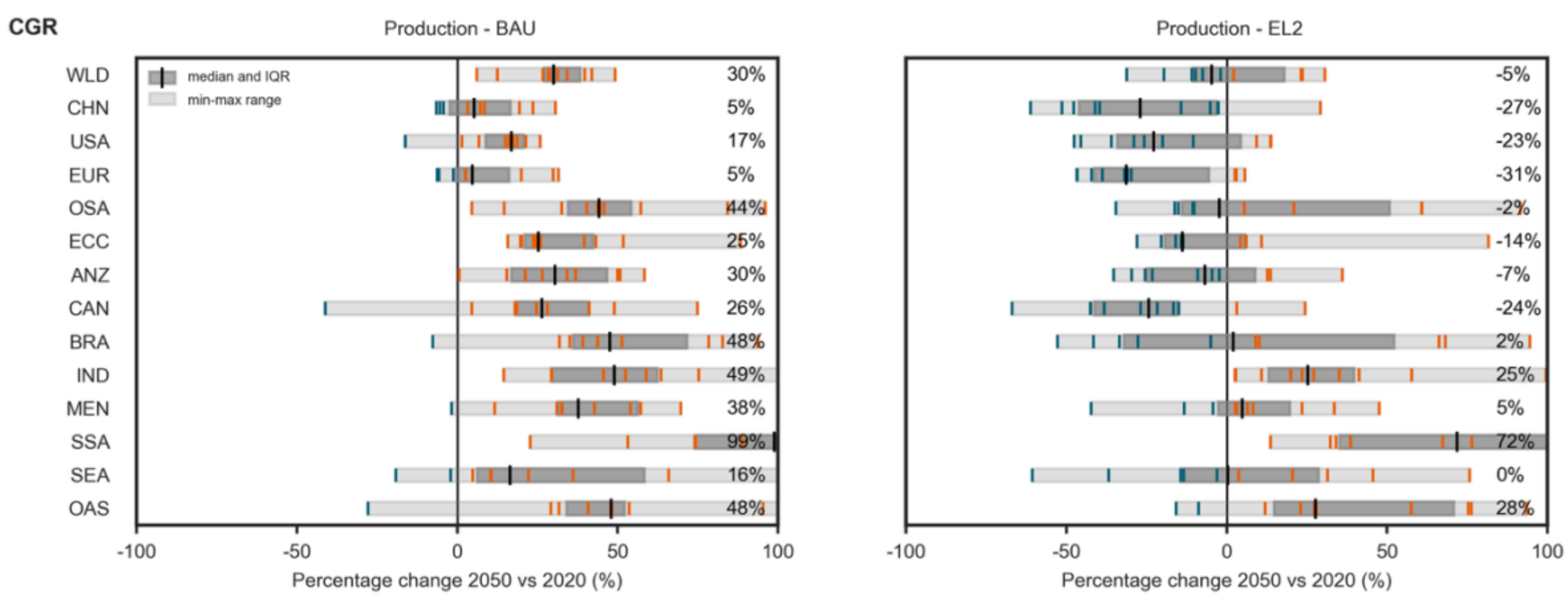
CGR
Production - BAU
Production - EL2
median and IQR
min-max range
WLD CHN USA EUR OSA ECC ANZ CAN BRA IND MEN SSA SEA OAS
30% 5% 17% 5% 44% 25% 30% 26% 48% 49% 38% 99% 16% 48%
-5% -27% -23% -31% -2% -14% -7% -24% 2% 25% 5% 72% 0% 28%
Percentage change 2050 vs 2020 (%)

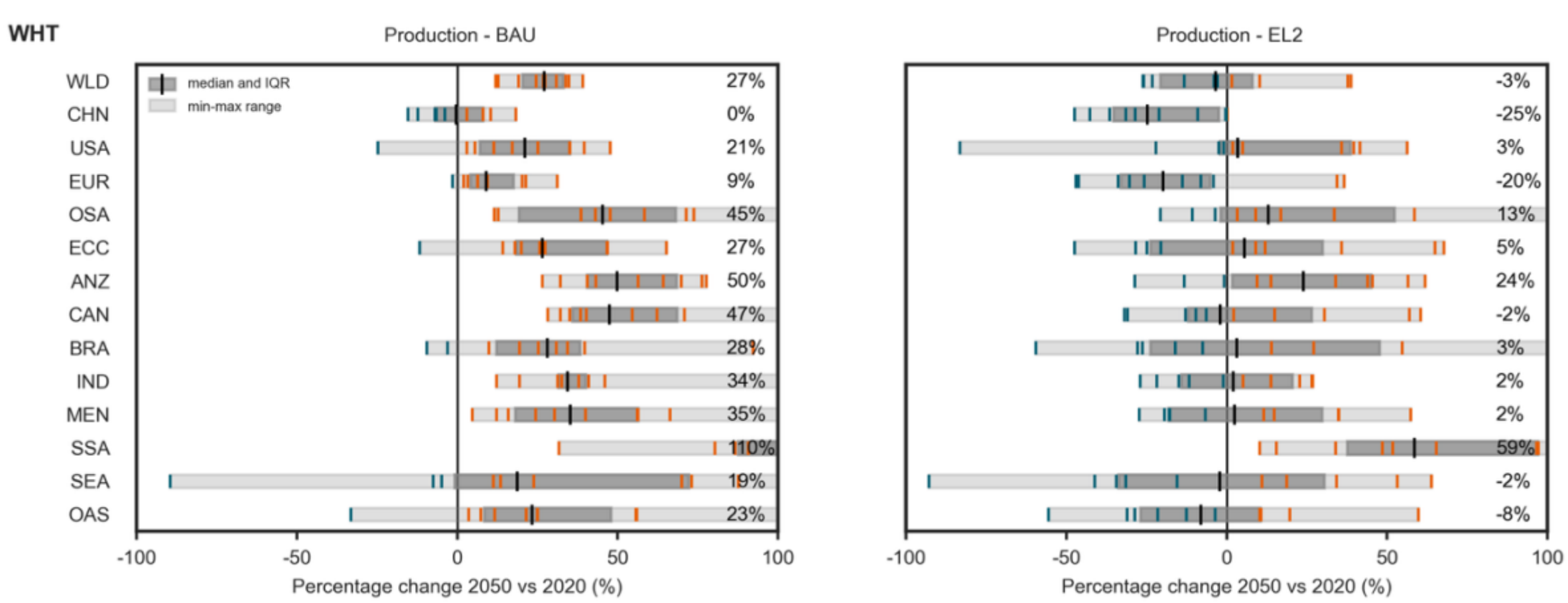
WHT
Production - BAU
Production - EL2
median and IQR
min-max range
WLD CHN USA EUR OSA ECC ANZ CAN BRA IND MEN SSA SEA OAS
27% 0% 21% 9% 45% 27% 50% 47% 28% 34% 35% 110% 19% 23%
-3% -25% 3% -20% 13% 5% 24% -2% 3% 2% 2% 59% -2% -8%
Percentage change 2050 vs 2020 (%)

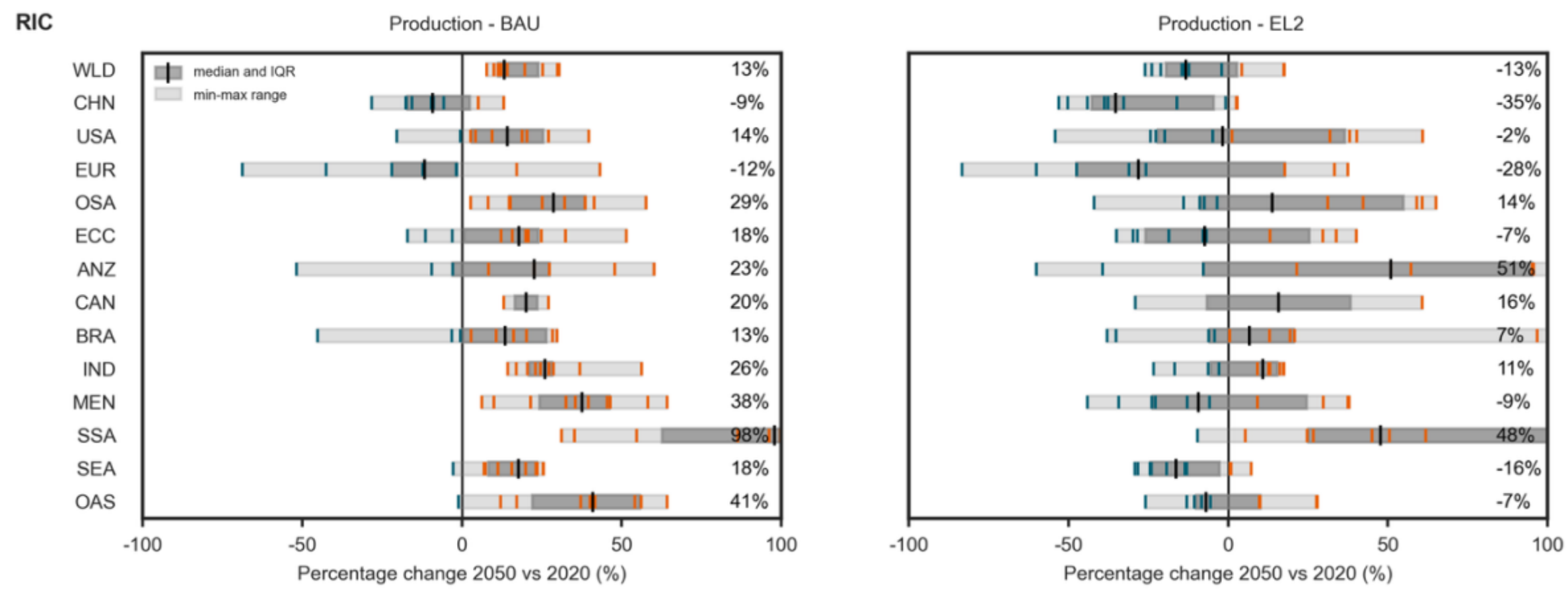

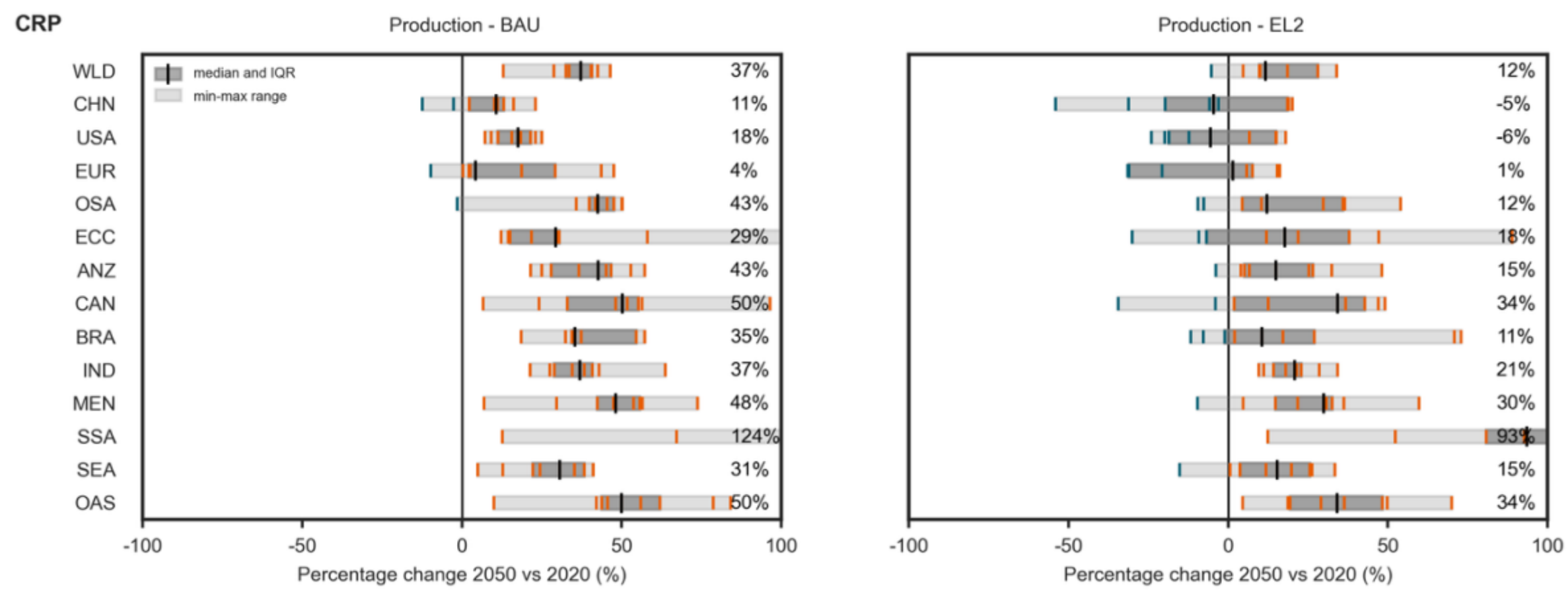

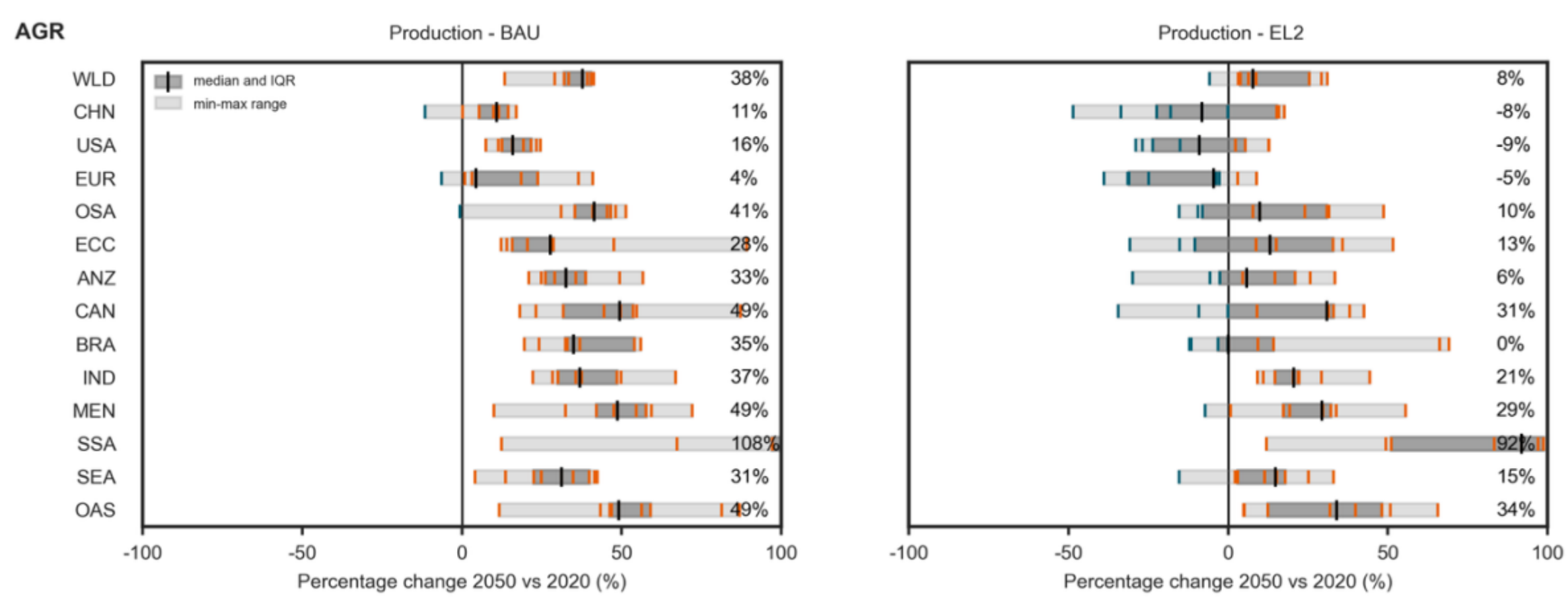

Supplementary Figure 7: Changes to production for BAU and EL2 scenarios by 2050 versus 2020 levels for 12 sectors/sector aggregates. *Results are given for the world ('WLD') and 13 global regions. Region codes are ANZ = Australia and New Zealand, BRA = Brazil; CAN = Canada; CHN = China; EUR = Europe; ECC = Eastern Europe, Caucasus, Central Asia; IND = India; MEN = Middle East and North Africa; OAS = Other Asia; OSA = Other South and Central America; SEA = Southeast Asia; SSA = Sub-Saharan Africa; USA = United States of America. Shaded areas give the range of results (dark grey = inter-quartile range, light grey = min-max range) with individual model results given by teal (percentage decrease) and orange (percentage increase) lines. Black vertical lines indicate median values which are also expressed numerically in each figure (on the right-hand side).*

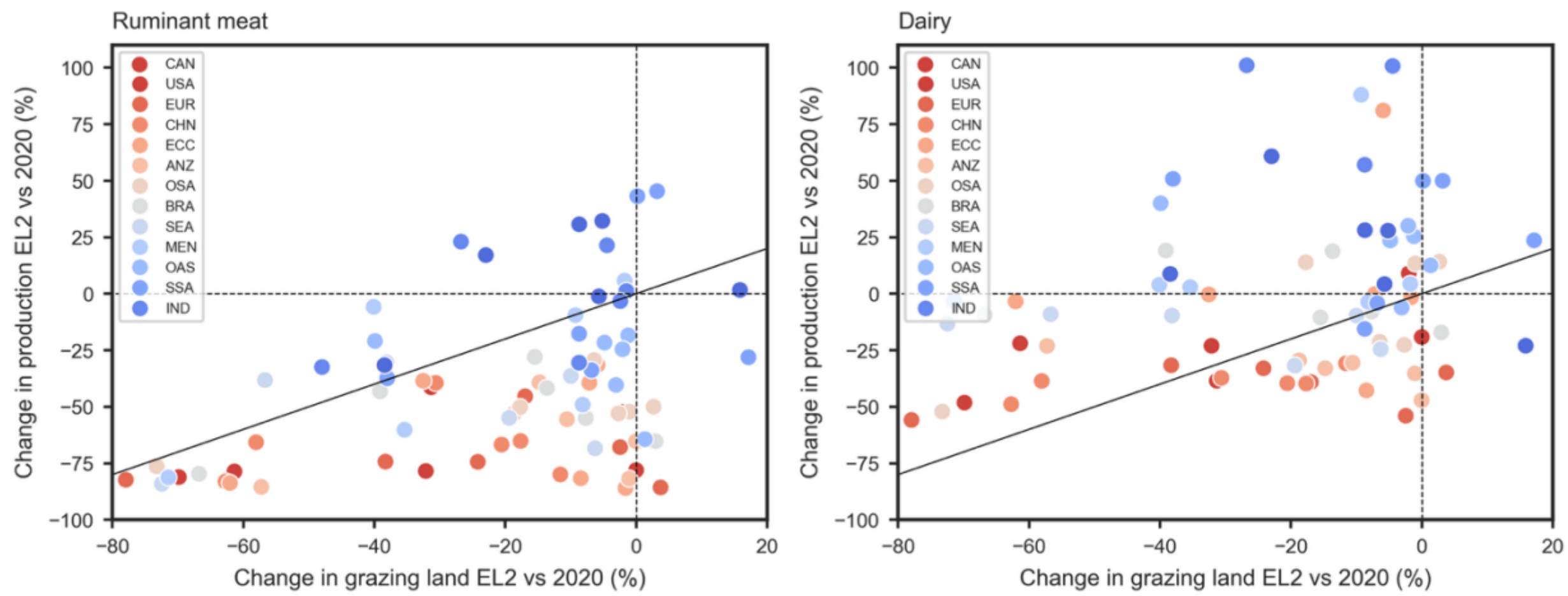

***Supplementary Figure 8: Scatterplots of relative changes in grazing land versus changes in ruminant meat and dairy in the EL2 scenario versus 2020 levels**. Results are given for 13 regions (coloured based on the size of change in ruminant production) for six models (AIM, GCAM, GLOBIOM, IMAGE, MAGNET, MAgPIE).*

***Supplementary Table 18: Median change in grazing land, ruminant meat and dairy production in EL2 scenario versus BAU 2050**. Ratios of production changes to grazing land changes are also given, as is ruminant yield results (inclusive of bovine and other ruminants) from IMPACT. Results are given for 13 global regions and the world.*

	**% change EL2 versus BAU 2050**			**Ratio of changes**		**IMPACT model RUM yields EL2 2050**
**Region**	**Grazing land (GRS)**	**Ruminant meat (RUM)**	**Dairy (DRY)**	**RUM:GRS**	**RUM:DRY**	**(kg/animal)**
ANZ	-4.6	-51.2	-3.9	3.7	2.0	64
BRA	-28.8	-46.6	-11.4	2.0	0.6	242
CAN	-31.7	-78.2	-22.5	1.9	0.7	361
CHN	-7.8	-60.5	-0.9	5.4	0.0	41
EUR	-25.6	-66.2	-39.1	2.3	1.6	100
ECC	-12.7	-60.4	-31.7	4.0	2.6	75
IND	-7.2	9.4	18.3	-0.3	-2.0	77
MEN	-2.6	-23.1	24.5	8.1	-2.9	61
OAS	-3.3	-22.9	36.8	3.5	1.6	40
OSA	-14.6	-49.1	-8.6	1.5	-0.2	179
SEA	-22.3	-29.3	3.4	1.1	-0.1	110
SSA	-6.6	-1.0	110.5	-0.1	-14.5	43
USA	-20.6	-74.3	-36.9	2.3	1.1	331
WLD	-9.0	-46.1	-3.3	3.5	0.4	67

Supplementary Table 18 shows that there are country/regional differences in the relationship between changes to grazing land and changes in ruminant meat production.

The ruminant production – grazing land relationship is modulated by each region's split between bovine and small ruminant (sheep and goat) production, yields (kg/animal), and the projected changes to dairy production which could have the effect of dampening or amplifying grazing land changes due to changing ruminant production. Another factor, not explicitly reported on by models in this study is the type and prevalence of different livestock production systems and the extent to which this can be modelled.

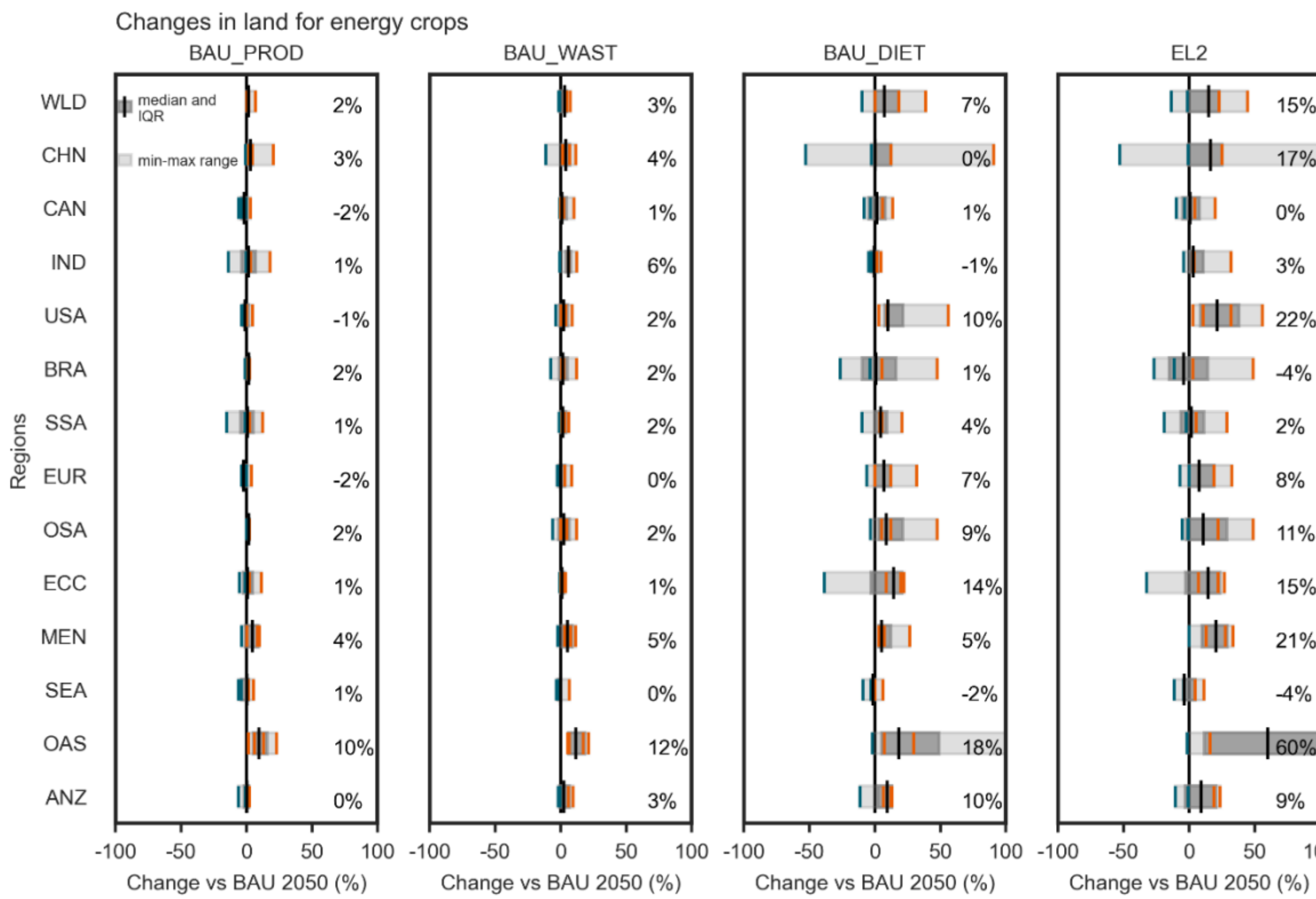

***Supplementary Figure 9: Changes in land for energy crops versus the BAU scenario for BAU_PROD, BAU_WAST, BAU_DIET and EL2 scenarios by 2050**. Results are given for the world ('WLD') and 13 global regions. Region codes are ANZ = Australia and New Zealand, BRA = Brazil; CAN = Canada, CHN = China; EUR = Europe; ECC = Eastern Europe, Caucasus, Central Asia; IND = India; MEN = Middle East and North Africa; OAS = Other Asia; OSA = Other South and Central America; SEA = Southeast Asia; SSA = Sub-Saharan Africa; USA = United States of America. Shaded areas give the range of results (dark grey = inter-quartile range, light grey = min-max range) with individual model results given by teal (percentage decrease) and orange (percentage increase) lines. Black vertical lines indicate median values which are also expressed numerically in each figure (on the right-hand side).*

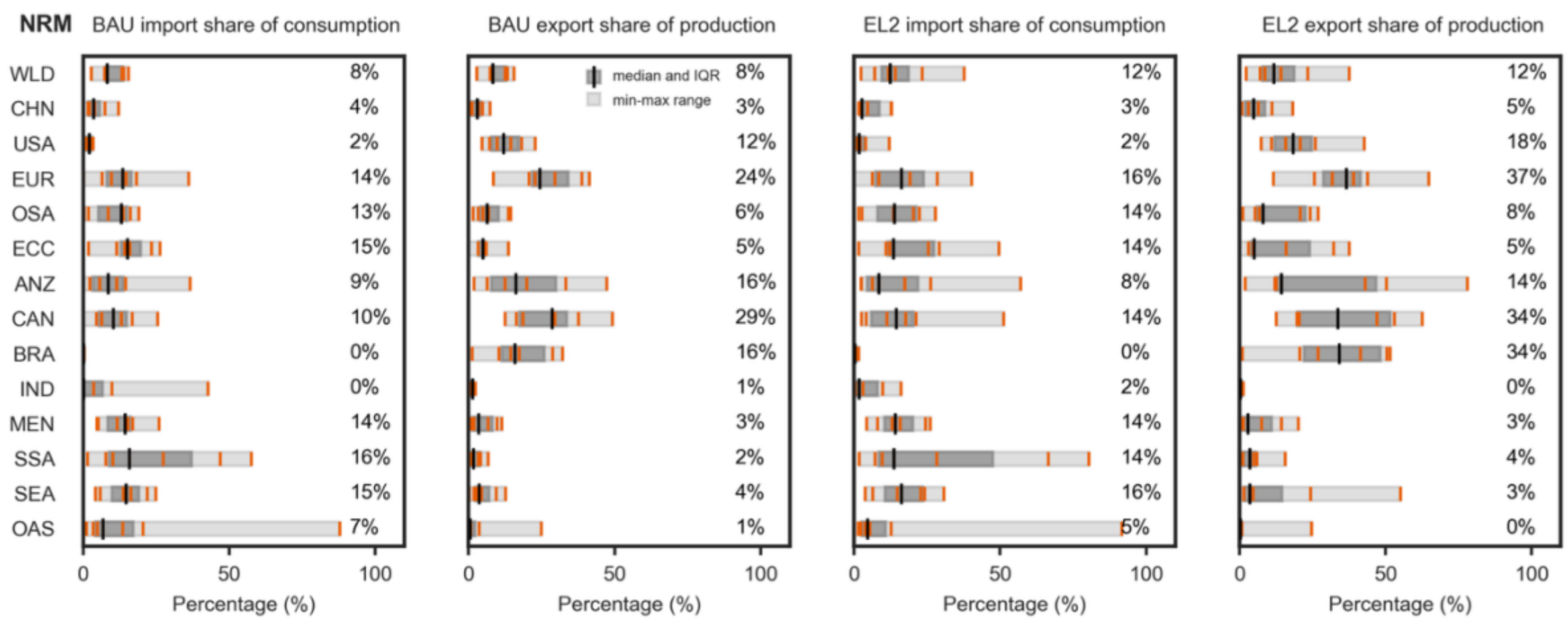
NRM
BAU import share of consumption
BAU export share of production
EL2 import share of consumption
EL2 export share of production
median and IQR
min-max range
WLD
CHN
USA
EUR
OSA
ECC
ANZ
CAN
BRA
IND
MEN
SSA
SEA
OAS
8% 4% 2% 14% 13% 15% 9% 10% 0% 0% 14% 16% 15% 7%
8% 3% 12% 24% 6% 5% 16% 29% 16% 1% 3% 2% 4% 1%
12% 3% 2% 16% 14% 14% 8% 14% 0% 2% 14% 14% 16% 5%
12% 5% 18% 37% 8% 5% 14% 34% 34% 0% 3% 4% 3% 0%
0 50 100
Percentage (%)

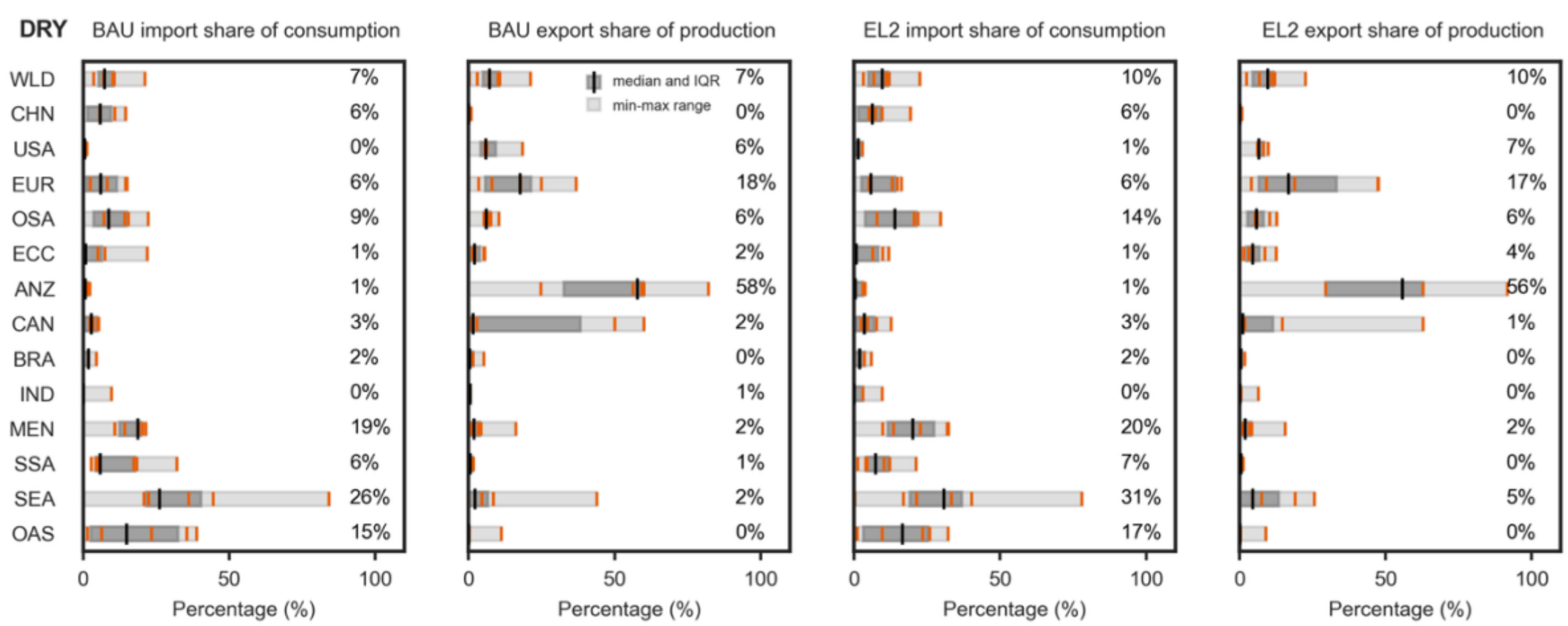
DRY
BAU import share of consumption
BAU export share of production
EL2 import share of consumption
EL2 export share of production
median and IQR
min-max range
WLD
CHN
USA
EUR
OSA
ECC
ANZ
CAN
BRA
IND
MEN
SSA
SEA
OAS
7% 6% 0% 6% 9% 1% 1% 3% 2% 0% 19% 6% 26% 15%
7% 0% 6% 18% 6% 2% 58% 2% 0% 1% 2% 1% 2% 0%
10% 6% 1% 6% 14% 1% 1% 3% 2% 0% 20% 7% 31% 17%
10% 0% 7% 17% 6% 4% 56% 1% 0% 0% 2% 0% 5% 0%
0 50 100
Percentage (%)

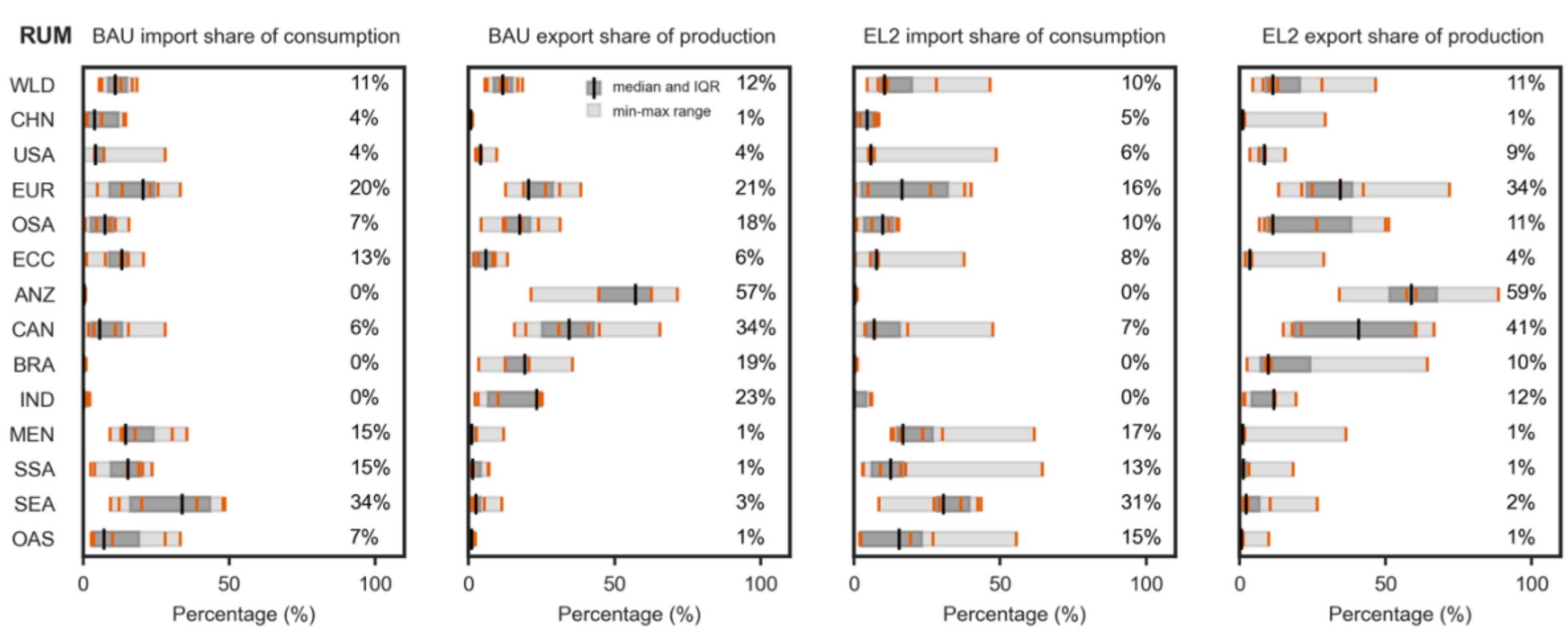
RUM
BAU import share of consumption
BAU export share of production
EL2 import share of consumption
EL2 export share of production
median and IQR
min-max range
WLD
CHN
USA
EUR
OSA
ECC
ANZ
CAN
BRA
IND
MEN
SSA
SEA
OAS
11% 4% 4% 20% 7% 13% 0% 6% 0% 0% 15% 15% 34% 7%
12% 1% 4% 21% 18% 6% 57% 34% 19% 23% 1% 1% 3% 1%
10% 5% 6% 16% 10% 8% 0% 7% 0% 0% 17% 13% 31% 15%
11% 1% 9% 34% 11% 4% 59% 41% 10% 12% 1% 1% 2% 1%
0 50 100
Percentage (%)

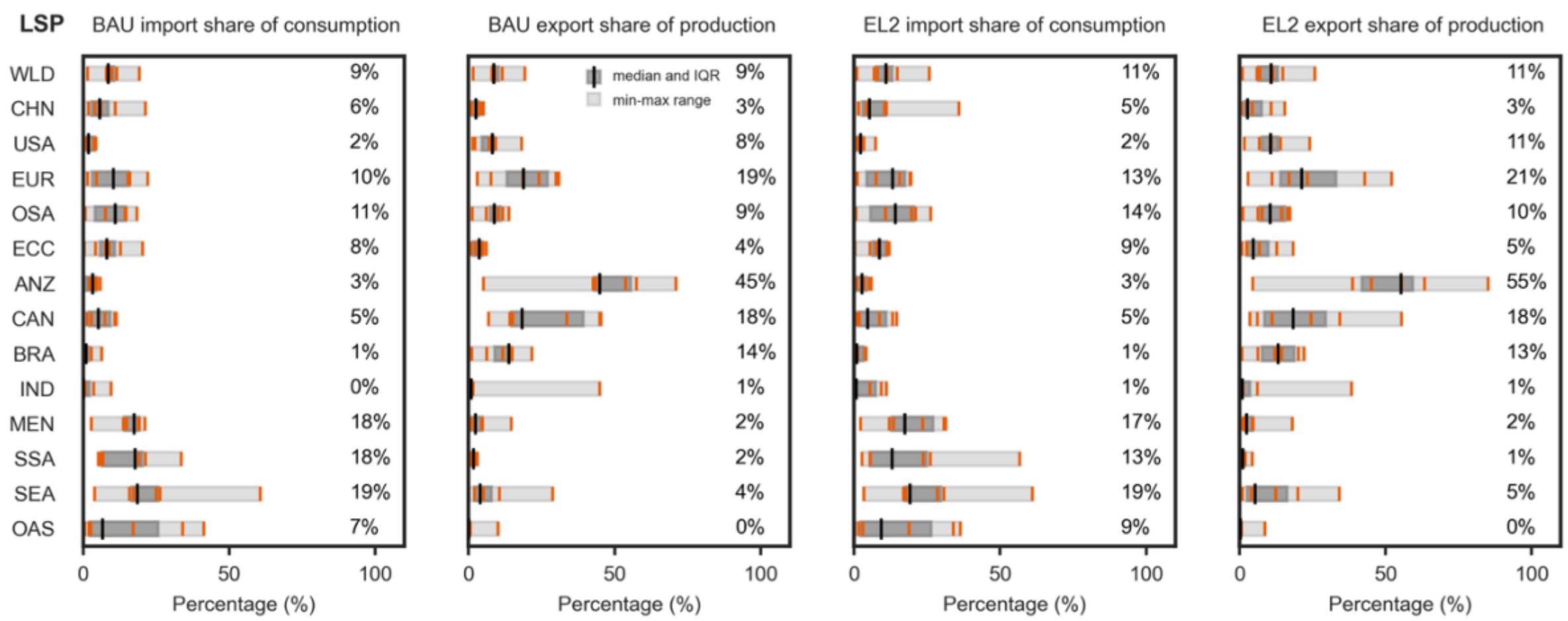
LSP
BAU import share of consumption
BAU export share of production
EL2 import share of consumption
EL2 export share of production
median and IQR
min-max range
WLD CHN USA EUR OSA ECC ANZ CAN BRA IND MEN SSA SEA OAS
9% 6% 2% 10% 11% 8% 3% 5% 1% 0% 18% 18% 19% 7%
9% 3% 8% 19% 9% 4% 45% 18% 14% 1% 2% 2% 4% 0%
11% 5% 2% 13% 14% 9% 3% 5% 1% 1% 17% 13% 19% 9%
11% 3% 11% 21% 10% 5% 55% 18% 13% 1% 2% 1% 5% 0%
0 50 100
Percentage (%)

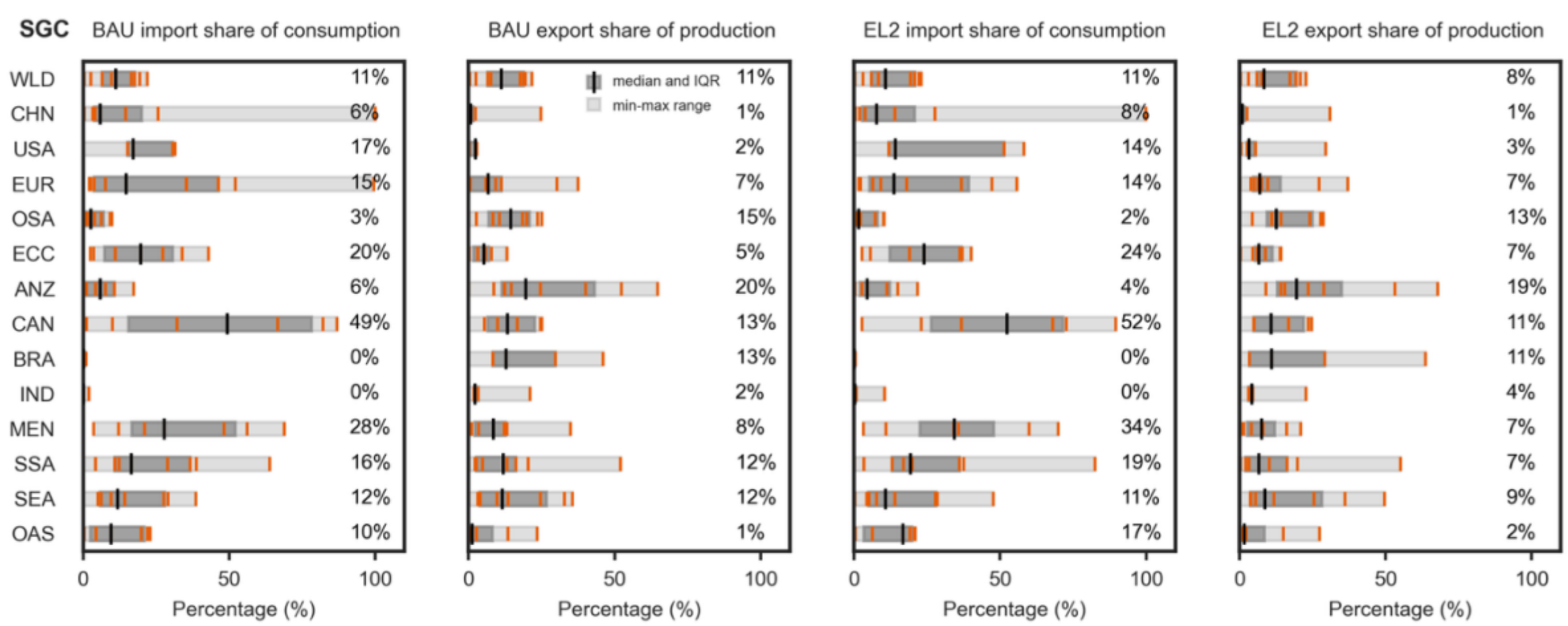
SGC
BAU import share of consumption
BAU export share of production
EL2 import share of consumption
EL2 export share of production
median and IQR
min-max range
WLD CHN USA EUR OSA ECC ANZ CAN BRA IND MEN SSA SEA OAS
11% 6% 17% 15% 3% 20% 6% 49% 0% 0% 28% 16% 12% 10%
11% 1% 2% 7% 15% 5% 20% 13% 13% 2% 8% 12% 12% 1%
11% 8% 14% 14% 2% 24% 4% 52% 0% 0% 34% 19% 11% 17%
8% 1% 3% 7% 13% 7% 19% 11% 11% 4% 7% 7% 9% 2%
0 50 100
Percentage (%)

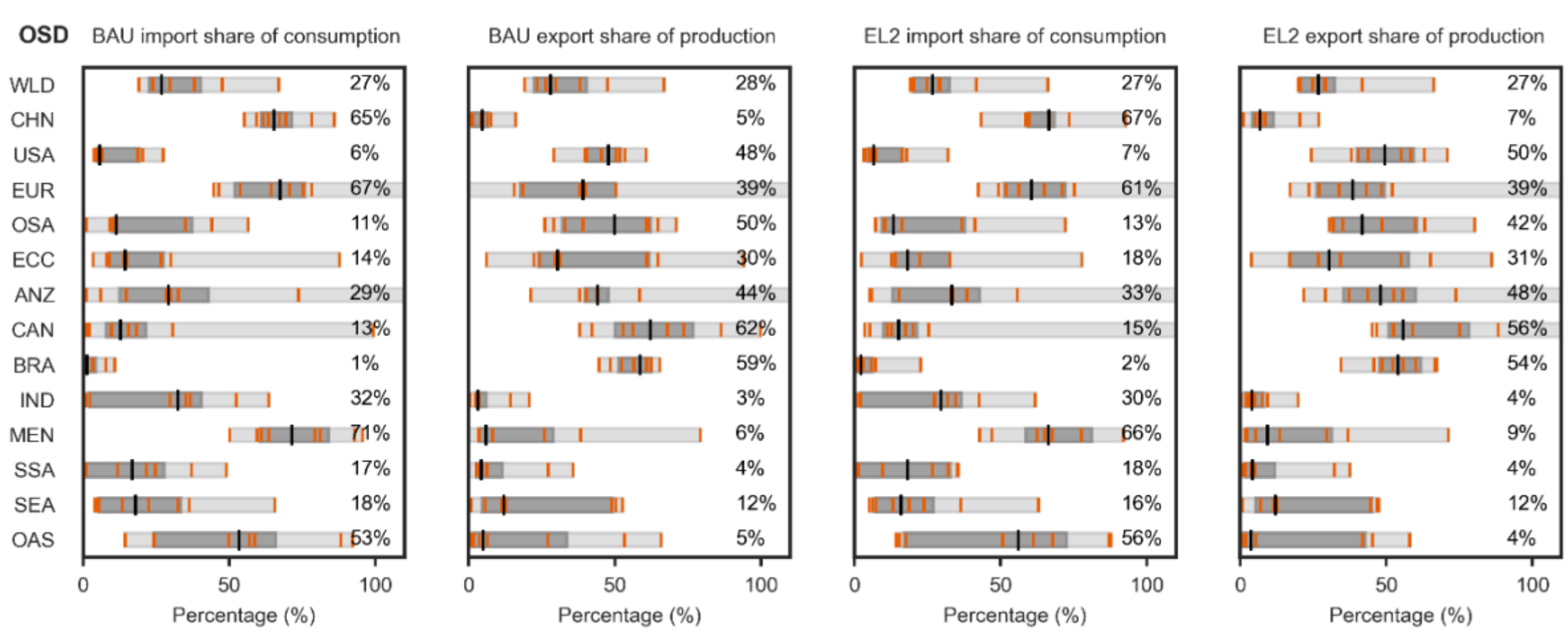
OSD
BAU import share of consumption
BAU export share of production
EL2 import share of consumption
EL2 export share of production
WLD CHN USA EUR OSA ECC ANZ CAN BRA IND MEN SSA SEA OAS
27% 65% 6% 67% 11% 14% 29% 13% 1% 32% 71% 17% 18% 53%
28% 5% 48% 39% 50% 30% 44% 62% 59% 3% 6% 4% 12% 5%
27% 67% 7% 61% 13% 18% 33% 15% 2% 30% 66% 18% 16% 56%
27% 7% 50% 39% 42% 31% 48% 56% 54% 4% 9% 4% 12% 4%
0 50 100
Percentage (%)

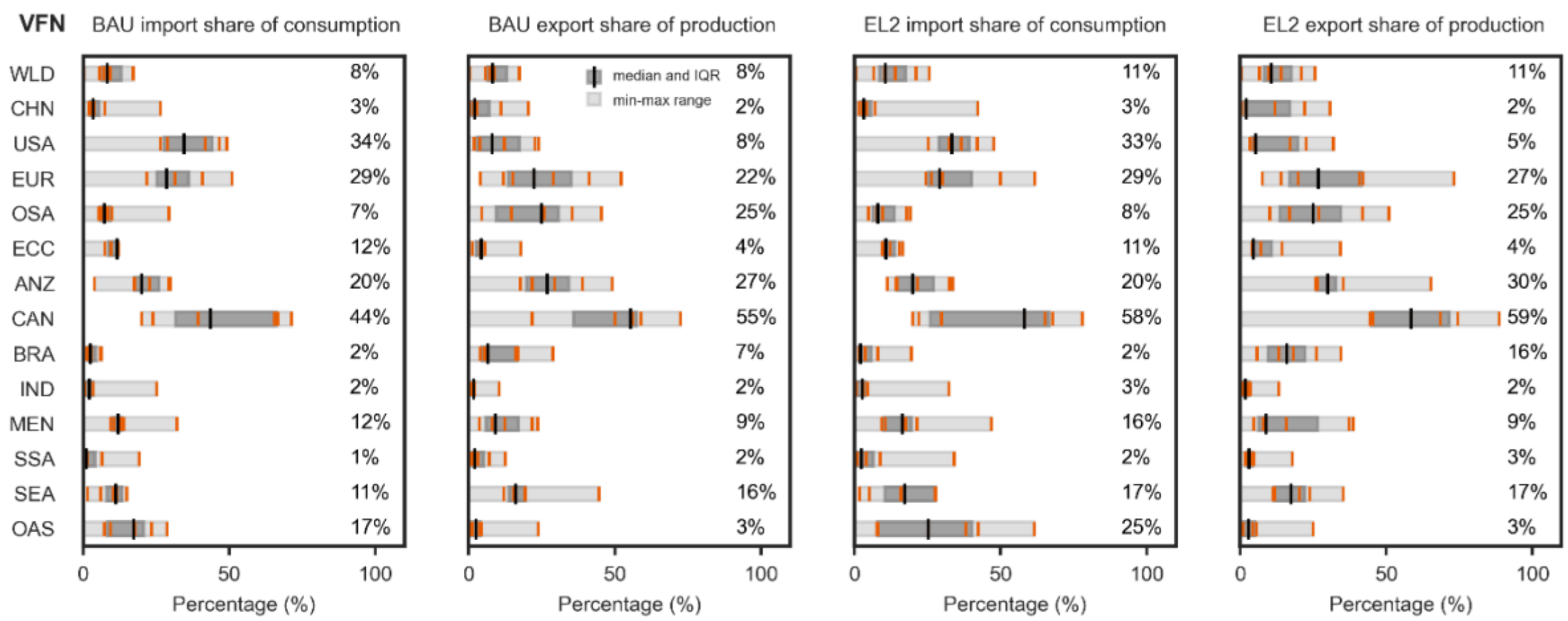
VFN
BAU import share of consumption
BAU export share of production
EL2 import share of consumption
EL2 export share of production
median and IQR
min-max range
WLD CHN USA EUR OSA ECC ANZ CAN BRA IND MEN SSA SEA OAS
8% 3% 34% 29% 7% 12% 20% 44% 2% 2% 12% 1% 11% 17%
8% 2% 8% 22% 25% 4% 27% 55% 7% 2% 9% 2% 16% 3%
11% 3% 33% 29% 8% 11% 20% 58% 2% 3% 16% 2% 17% 25%
11% 2% 5% 27% 25% 4% 30% 59% 16% 2% 9% 3% 17% 3%
0 50 100
Percentage (%)

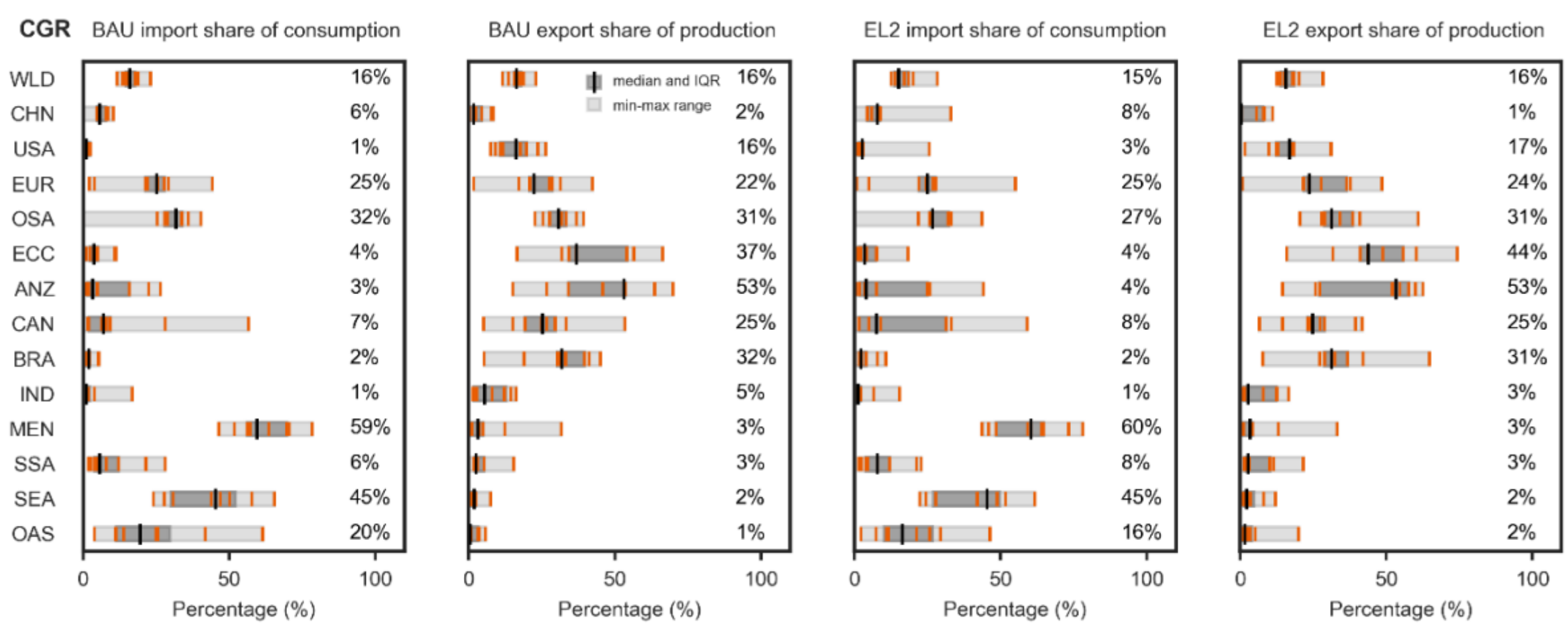
CGR
BAU import share of consumption
BAU export share of production
EL2 import share of consumption
EL2 export share of production
median and IQR
min-max range
WLD CHN USA EUR OSA ECC ANZ CAN BRA IND MEN SSA SEA OAS
16% 6% 1% 25% 32% 4% 3% 7% 2% 1% 59% 6% 45% 20%
16% 2% 16% 22% 31% 37% 53% 25% 32% 5% 3% 3% 2% 1%
15% 8% 3% 25% 27% 4% 4% 8% 2% 1% 60% 8% 45% 16%
16% 1% 17% 24% 31% 44% 53% 25% 31% 3% 3% 3% 2% 2%
0 50 100
Percentage (%)

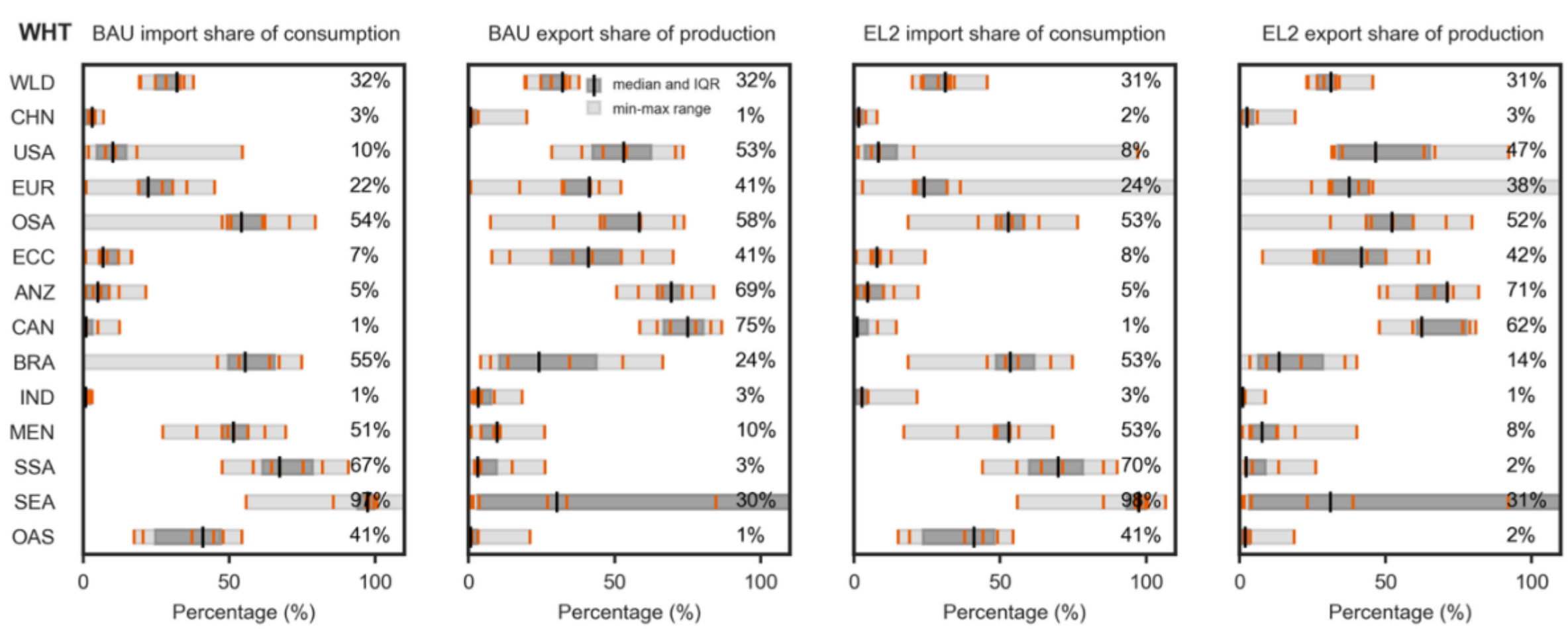
WHT
BAU import share of consumption
BAU export share of production
EL2 import share of consumption
EL2 export share of production
median and IQR
min-max range
WLD CHN USA EUR OSA ECC ANZ CAN BRA IND MEN SSA SEA OAS
32% 3% 10% 22% 54% 7% 5% 1% 55% 1% 51% 67% 97% 41%
32% 1% 53% 41% 58% 41% 69% 75% 24% 3% 10% 3% 30% 1%
31% 2% 8% 24% 53% 8% 5% 1% 53% 3% 53% 70% 98% 41%
31% 3% 47% 38% 52% 42% 71% 62% 14% 1% 8% 2% 31% 2%
0 50 100
Percentage (%)

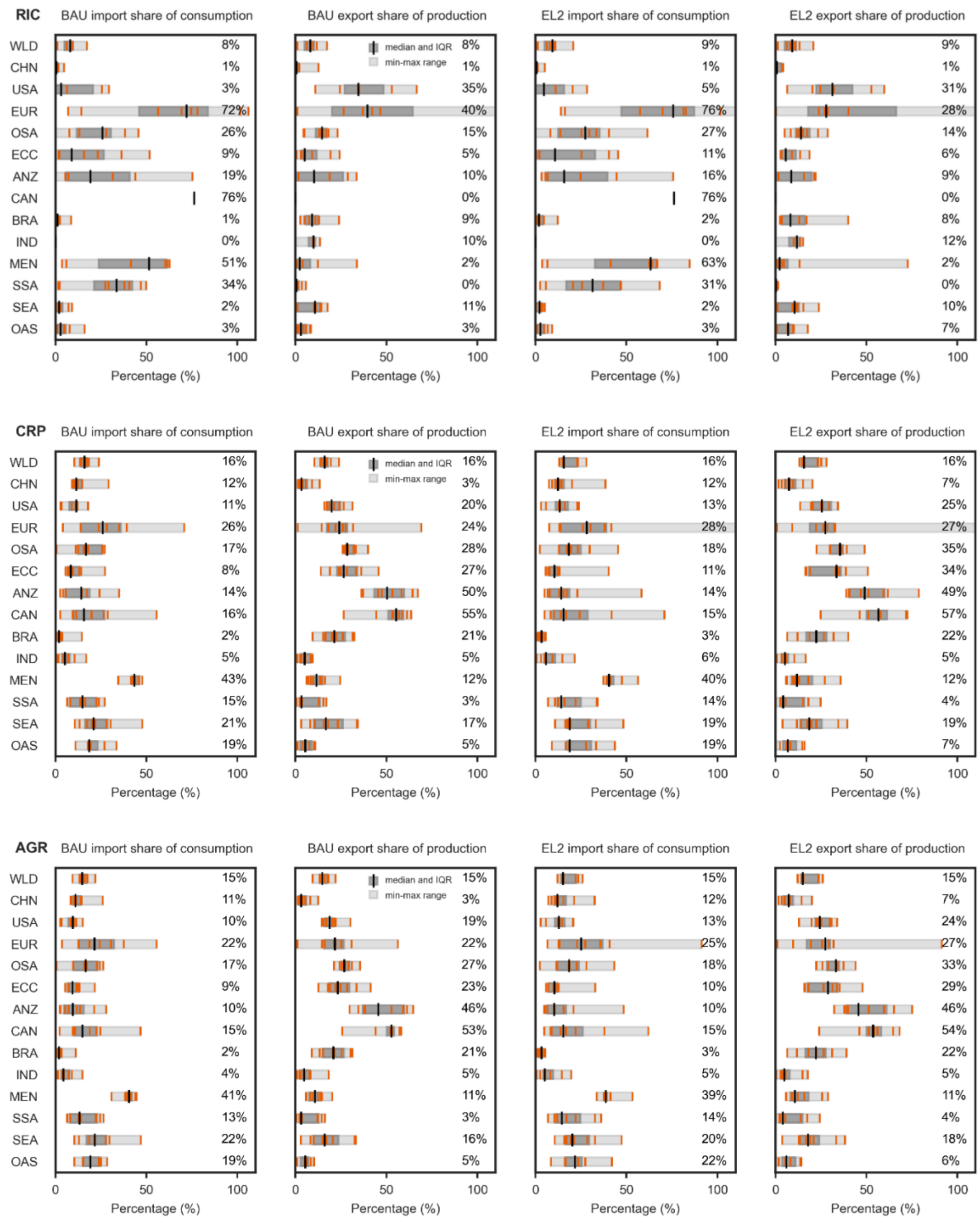

Supplementary Figure 10: Imports as a share of consumption and exports as share of production for BAU and EL2 scenarios for 12 sectors/sector aggregates. *Results are given for the world ('WLD') and 13 global regions. Region codes are ANZ = Australia and New Zealand, BRA = Brazil; CAN = Canada; CHN = China; EUR = Europe; ECC = Eastern Europe, Caucasus, Central Asia; IND = India; MEN = Middle East and North Africa; OAS = Other Asia; OSA = Other South and Central America; SEA = Southeast Asia; SSA = Sub-Saharan Africa; USA = United States of America. Shaded areas give the range of results (dark grey = inter-quartile range, light grey = min-max range) with individual model results given by teal (percentage decrease) and orange (percentage increase) lines.*

Black vertical lines indicate median values which are also expressed numerically in each figure (on the right-hand side).

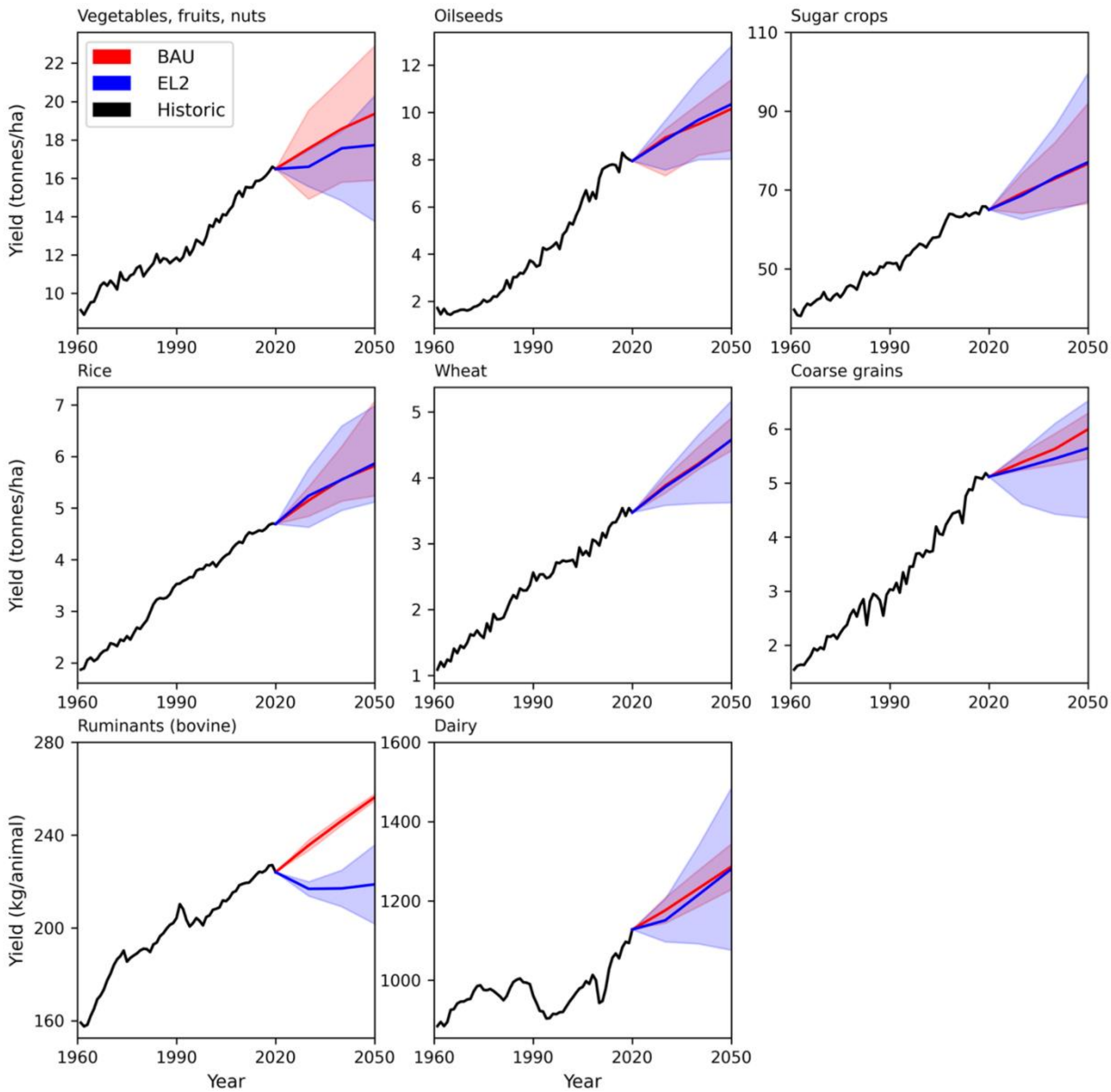

Supplementary Figure 11: Changes in yield (kg/animal and tonnes/ha)) from 1961 to 2020 (black) and then projections under BAU (red) and EL2 (blue) scenarios*. Scenario shaded areas are bounded by upper and lower ensemble model results with the median result plotted as a solid line. Historic trends use FAO FAOSTAT data[69].*

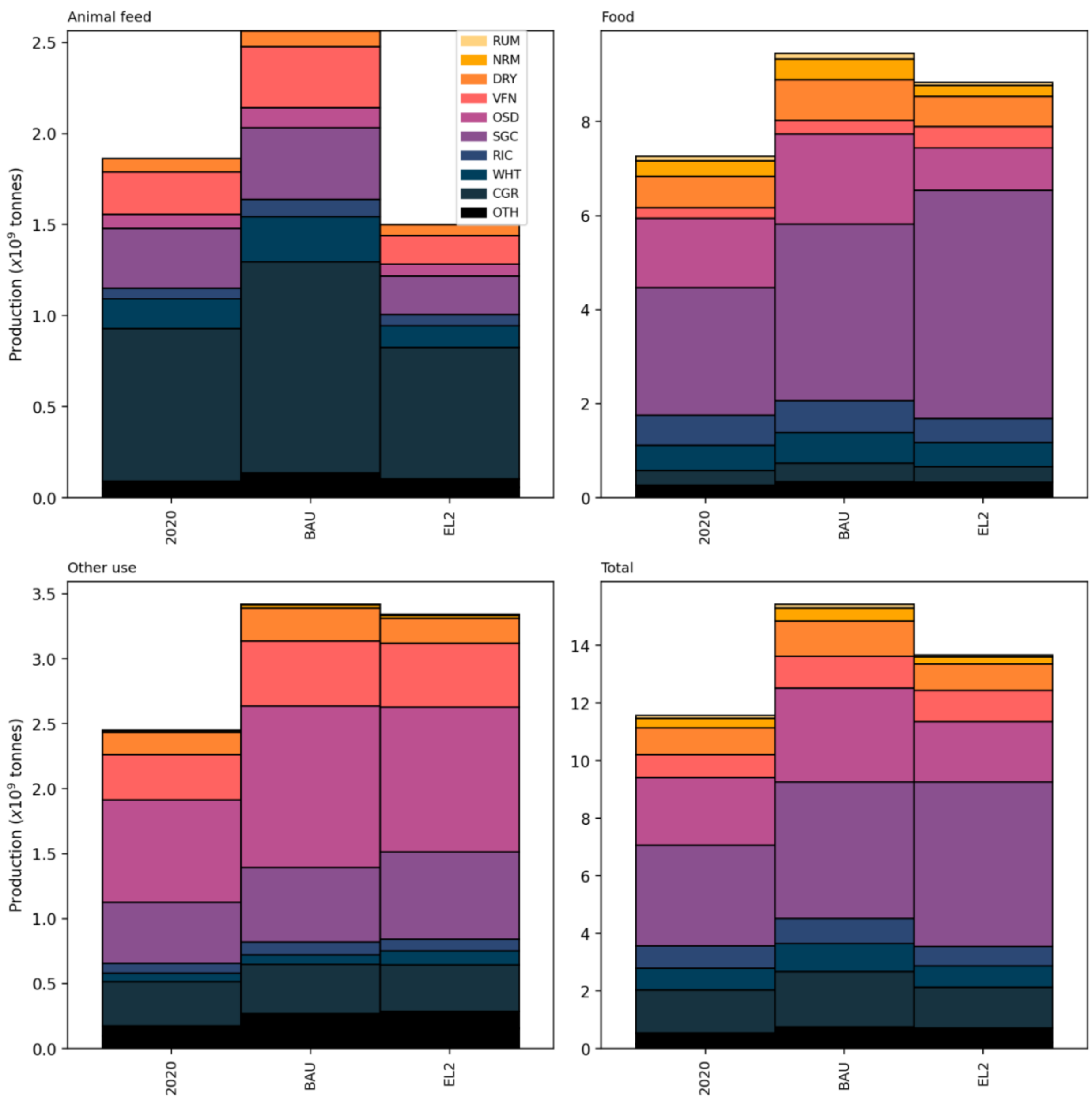

Supplementary Figure 12: Breakdown of production by food sector for different end uses (animal feed, food, other use) for 2020, BAU and EL2. *Total is the sum of the three end uses. All results are model ensemble medians. RUM = ruminant meat, NRM = non ruminant meat (poultry, pork, eggs), DRY = dairy, VFN = vegetables, fruits, and nuts, OSD = oilseeds, SGC = sugar crops, RIC = rice, WHT = wheat, CGR = coarse grains, OTH = other.*

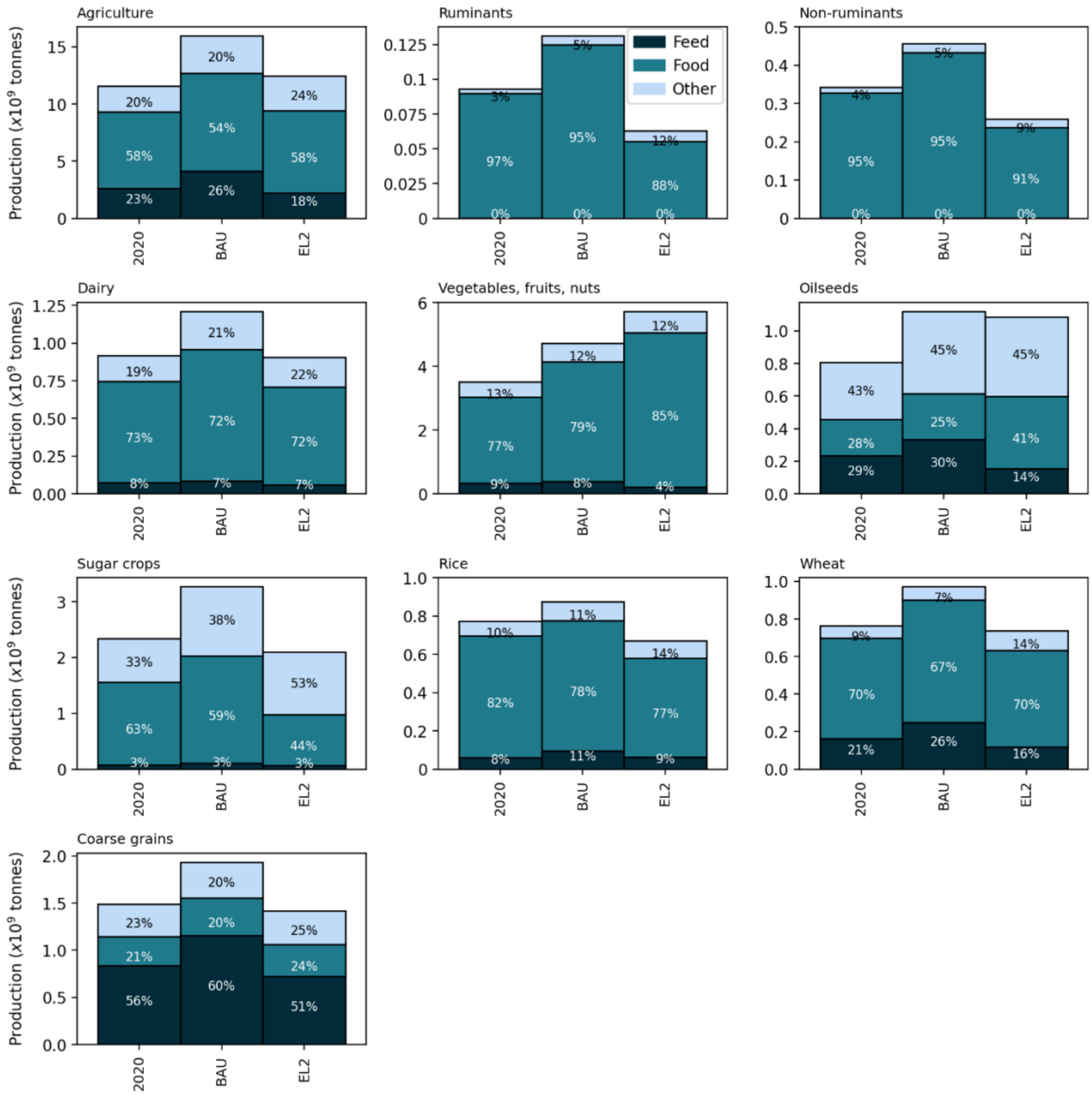

Supplementary Figure 13: Production (billion tonnes) use shares – feed, food, and other use from dark to light shades – by food sector for 2020, BAU and EL2. *Feed refers to animal feed, food refers to direct human demand, and other use refers to industrial or fuel use. Percentage shares are given for each segment. All results are model ensemble medians.*

References

1. Thornton P, Nelson G, Mayberry D, Herrero M. Impacts of heat stress on global cattle production during the 21st century: a modelling study. The Lancet Planetary Health. 2022 Mar 1;6(3):e192–201. doi:10.1016/S2542-5196(22)00002-X PubMed PMID: 35278386.

2. Nelson GC, Vanos J, Havenith G, Jay O, Ebi KL, Hijmans RJ. Global reductions in manual agricultural work capacity due to climate change. Global Change Biology. 2024;30(1):e17142. doi:10.1111/gcb.17142

3. Chepeliev M. GTAP-Power Data Base: Version 10. Journal of Global Economic Analysis. 2020 Dec 11;5(2):2. doi:10.21642/JGEA.050203AF

4. Chepeliev M. Incorporating Nutritional Accounts to the GTAP Data Base. Journal of Global Economic Analysis. 2022 Jun 25;7(1):1. doi:10.21642/JGEA.070101AF

5. Gatto A, Chepeliev M. Global food loss and waste estimates show increasing nutritional and environmental pressures. Nat Food. 2024 Feb;5(2):136–47. doi:10.1038/s43016-023-00915-6

6. Calvin K, Patel P, Clarke L, Asrar G, Bond-Lamberty B, Cui RY, et al. GCAM v5.1: representing the linkages between energy, water, land, climate, and economic systems. Geoscientific Model Development. 2019 Feb 15;12(2):677–98. doi:10.5194/gmd-12-677-2019

7. Zhao X, Mignone BK, Wise MA, McJeon HC. Trade-offs in land-based carbon removal measures under 1.5 °C and 2 °C futures. Nat Commun. 2024 Mar 14;15(1):2297. doi:10.1038/s41467-024-46575-3

8. Kindermann GE, Obersteiner M, Rametsteiner E, McCallum I. Predicting the deforestation-trend under different carbon-prices. Carbon Balance Manage. 2006 Dec 6;1(1):15. doi:10.1186/1750-0680-1-15

9. Gusti M, Havlik P, Obersteiner M. Technical Description of the IIASA Model Cluster [Other] [Internet]. 2008 [cited 2024 Nov 21]. Available from: http://digital.library.unt.edu/ark:/67531/metadc13707

10. Doelman JC, Beier FD, Stehfest E, Bodirsky BL, Beusen AHW, Humpenöder F, et al. Quantifying synergies and trade-offs in the global water-land-food-climate nexus using a multi-model scenario approach. Environ Res Lett. 2022 Mar;17(4):045004. doi:10.1088/1748-9326/ac5766

11. Stehfest, E., van Vuuren, D., Kram, T., Bouwman, L., Alkemade, R., Bakkenes, M., Biemans, H., Bouwman, A., den Elzen, M., Janse, J., Lucas, P., van Minnen, J., Müller, M., Prins, A., Elke Stehfest, Detlef van Vuuren, Tom Kram, Lex Bouwman, Rob Alkemade, Michel Bakkenes, Hester Biemans (WUR), Arno Bouwman, Michel den Elzen, Jan Janse, Paul Lucas, Jelle van Minnen, Christoph Müller, Anne Gerdien Prins. Integrated Assessment of Global Environmental Change with IMAGE 3.0 - Model description and policy applications [Internet]. Netherlands: PBL; 2014 Jul [cited 2024 Nov 21]. Report No. Available from: https://www.pbl.nl/en/publications/integrated-assessment-of-global-environmental-change-with-image-30-model-description-and-policy-applications

12. Doelman JC, Stehfest E, Tabeau A, van Meijl H, Lassaletta L, Gernaat DEHJ, et al. Exploring SSP land-use dynamics using the IMAGE model: Regional and gridded

scenarios of land-use change and land-based climate change mitigation. Global Environmental Change. 2018;48:119–35. doi:10.1016/j.gloenvcha.2017.11.014

13. Woltjer GB, Kuiper M, Kavallari A, Meijl H van, Powell JP, Rutten MM, et al. The MAGNET Model: Module description [Internet]. 2014 [cited 2024 Nov 21]. Available from: https://research.wur.nl/en/publications/the-magnet-model-module-description

14. Schaphoff S, von Bloh W, Rammig A, Thonicke K, Biemans H, Forkel M, et al. LPJmL4 – a dynamic global vegetation model with managed land – Part 1: Model description. Geoscientific Model Development. 2018 Apr 12;11(4):1343–75. doi:10.5194/gmd-11-1343-2018

15. Müller C, Stehfest E, Minnen JG van, Strengers B, Bloh W von, Beusen AHW, et al. Drivers and patterns of land biosphere carbon balance reversal. Environ Res Lett. 2016 Mar;11(4):044002. doi:10.1088/1748-9326/11/4/044002

16. Beusen AHW, Van Beek LPH, Bouwman AF, Mogollón JM, Middelburg JJ. Coupling global models for hydrology and nutrient loading to simulate nitrogen and phosphorus retention in surface water - description of IMAGE–GNM and analysis of performance. Geoscientific Model Development. 2015 Dec 21;8(12):4045–67. doi:10.5194/gmd-8-4045-2015

17. Meinshausen M, Raper SCB, Wigley TML. Emulating coupled atmosphere-ocean and carbon cycle models with a simpler model, MAGICC6 – Part 1: Model description and calibration. Atmospheric Chemistry and Physics. 2011 Feb 16;11(4):1417–56. doi:10.5194/acp-11-1417-2011

18. den Elzen M, Meinshausen M, van Vuuren D. Multi-gas emission envelopes to meet greenhouse gas concentration targets: Costs versus certainty of limiting temperature increase. Global Environmental Change. 2007 May 1;17(2):260–80. doi:10.1016/j.gloenvcha.2006.10.003

19. Corong EL, Hertel TW, McDougall R, Tsigas ME, Mensbrugghe D van der. The Standard GTAP Model, Version 7. Journal of Global Economic Analysis. 2017 Jun 22;2(1):1. doi:10.21642/JGEA.020101AF

20. Aguiar A, Chepeliev M, Corong E, Mensbrugghe D van der. The Global Trade Analysis Project (GTAP) Data Base: Version 11. Journal of Global Economic Analysis. 2022;7(2):2. doi:10.21642/JGEA.070201AF

21. Dixon J, Scura L, Carpenter R, Sherman P. Economic Analysis of Environmental Impacts. 2nd edn. London: Routledge; 2013. 224 p. doi:10.4324/9781315070438

22. van Meijl H, van Rheenen T, Tabeau A, Eickhout B. The impact of different policy environments on agricultural land use in Europe. Agriculture, Ecosystems & Environment. 2006 May 1;Scenario-Based Studies of Future Land Use in Europe114(1):21–38. doi:10.1016/j.agee.2005.11.006

23. Herrendorf B, Schoellman T. Wages, Human Capital, and Barriers to Structural Transformation. American Economic Journal: Macroeconomics. 2018 Apr;10(2):1–23. doi:10.1257/mac.20160236

24. Dietrich JP, Bodirsky BL, Humpenöder F, Weindl I, Stevanović M, Karstens K, et al. MAgPIE 4 – a modular open-source framework for modeling global land systems. Geoscientific Model Development. 2019 Apr 3;12(4):1299–317. doi:10.5194/gmd-12-1299-2019

25. Baumstark L, Bauer N, Benke F, Bertram C, Bi S, Gong CC, et al. REMIND2.1: transformation and innovation dynamics of the energy-economic system within climate and sustainability limits. Geoscientific Model Development. 2021 Oct 28;14(10):6571–603. doi:10.5194/gmd-14-6571-2021

26. von Bloh W, Schaphoff S, Müller C, Rolinski S, Waha K, Zaehle S. Implementing the nitrogen cycle into the dynamic global vegetation, hydrology, and crop growth model LPJmL (version 5.0). Geoscientific Model Development. 2018 Jul 12;11(7):2789–812. doi:10.5194/gmd-11-2789-2018

27. Bodirsky BL, Dietrich JP, Martinelli E, Stenstad A, Pradhan P, Gabrysch S, et al. The ongoing nutrition transition thwarts long-term targets for food security, public health and environmental protection. Sci Rep. 2020 Nov 18;10(1):19778. doi:10.1038/s41598-020-75213-3

28. Humpenöder F, Karstens K, Lotze-Campen H, Leifeld J, Menichetti L, Barthelmes A, et al. Peatland protection and restoration are key for climate change mitigation. Environ Res Lett. 2020 Oct;15(10):104093. doi:10.1088/1748-9326/abae2a

29. Humpenöder F, Popp A, Dietrich JP, Klein D, Lotze-Campen H, Bonsch M, et al. Investigating afforestation and bioenergy CCS as climate change mitigation strategies. Environ Res Lett. 2014 Jun;9(6):064029. doi:10.1088/1748-9326/9/6/064029

30. IPCC. Climate Change 2021 – The Physical Science Basis: Working Group I Contribution to the Sixth Assessment Report of the Intergovernmental Panel on Climate Change [Internet]. 1st edn. Cambridge University Press; 2023 [cited 2023 Nov 7]. Available from: https://www.cambridge.org/core/product/identifier/9781009157896/type/book doi:10.1017/9781009157896

31. FAO. FAO Statistical yearbook 2013: World food and agriculture [Internet]. Rome: FAO; 2013 [cited 2025 Jul 18]. Available from: https://library.unccd.int/Details/books/172

32. von Lampe M, Willenbockel D, Ahammad H, Blanc E, Cai Y, Calvin K, et al. Why do global long-term scenarios for agriculture differ? An overview of the AgMIP Global Economic Model Intercomparison. Agricultural Economics. 2014;45(1):3–20. doi:10.1111/agec.12086

33. Hanasaki N, Fujimori S, Yamamoto T, Yoshikawa S, Masaki Y, Hijioka Y, et al. A global water scarcity assessment under Shared Socio-economic Pathways - Part 2: Water

availability and scarcity. Hydrology and Earth System Sciences. 2013 Jul 1;17(7):2393–413. doi:10.5194/hess-17-2393-2013

34. Hasegawa T, Fujimori S, Takahashi K, Masui T. Scenarios for the risk of hunger in the twenty-first century using Shared Socioeconomic Pathways. Environ Res Lett. 2015 Jan;10(1):014010. doi:10.1088/1748-9326/10/1/014010

35. Gustavsson J, Cederberg C, Sonesson U, Van Otterdijk R, Meybeck A. Global Food Losses and Food Waste [Internet]. 2011. Report Nos: 978-92-5-107205–9. Available from: http://www.fao.org/3/a-i2697e.pdf

36. Thom F. Fundamental Changes in Food: Improving the Toolbox of Simulation Modeling [doctoralThesis] [Internet]. 2025 [cited 2025 Jul 18]. Available from: https://ediss.uni-goettingen.de/handle/11858/15823 doi:10.53846/goediss-11080

37. Rieger J, Freund F, Offermann F, Geibel I, Gocht A. From fork to farm: Impacts of more sustainable diets in the EU-27 on the agricultural sector. Journal of Agricultural Economics. 2023;74(3):764–84. doi:10.1111/1477-9552.12530

38. CAPRI Consortium. CAPRI Online Manual [Internet]. 2022 [cited 2025 Jul 17]. Available from: https://www.capri-model.org/dokuwiki_help/doku.php?id=start

39. FAO. Food balance sheets 2010–2021. Global, regional and country trends. [Internet]. 2023;FAOSTAT AnalyticalBrief Series No. 72. doi:https://doi.org/10.4060/cc8088en

40. Sands RD, Meade B, Seale JL, Robinson S, Seeger R. Scenarios of Global Food Consumption: Implications for Agriculture [Internet]. 2023;Economic Research Report Number 323. doi:https://doi.org/10.32747/2023.8134356.ers

41. Alexandratos N, Bruinsma J. World agriculture towards 2030/2050: the 2012 revision [Internet]. FAO; 2012 [cited 2025 Jul 18]. (ESA Working Papers 12-03). Report No. Available from: https://ageconsearch.umn.edu/record/288998 doi:10.22004/ag.econ.288998

42. Edmonds JA, Link R, Waldhoff ST, Cui R. A global food demand model for the assessment of complex human-earth systems. Clim Change Econ. 2017 Nov;08(04):1750012. doi:10.1142/S2010007817500129

43. Zhao X, Chepeliev M, Patel P, Wise M, Calvin K, Narayan K, et al. gcamfaostat: An R package to prepare, process, and synthesize FAOSTAT data for global agroeconomic and multisector dynamic modeling. Journal of Open Source Software. 2024 Apr 22;9(96):6388. doi:10.21105/joss.06388

44. Zhao X, Chepeliev M, Escobar N, Binsted MT, Patel P, Kyle P, et al. Traceable and Scalable Food Balance Sheets from Agricultural Commodity Supply and Utilization Accounts (2010–2022). Sci Data. 2025 May 21;12(1):834. doi:10.1038/s41597-025-05137-y

45. Andrew Muhammad, James L. Seale Jr., Birgit Meade, Anita Regmi. International Evidence on Food Consumption Patterns: An Update Using 2005 International Comparison Program Data [Internet]. U.S. Dept. of Agriculture, Econ. Res. Serv; 2011 [cited 2025 Jul 18]. (TB-1929). Report No. Available from: https://www.ers.usda.gov/publications/pub-details?pubid=47581

46. de Lange T, van Dijk M, Kuiper M, van Zeist WJ, Bartelings H, Mizan A, et al. Socio-economic, environmental and health impacts of dietary transformation in Bangladesh. Environ Res Lett. 2024 Dec;20(1):014057. doi:10.1088/1748-9326/ada0ca

47. Gatto A, Kuiper M, van Meijl H. Economic, social and environmental spillovers decrease the benefits of a global dietary shift. Nat Food. 2023 Jun;4(6):6. doi:10.1038/s43016-023-00769-y

48. Doelman JC, Stehfest E, Tabeau A, van Meijl H, Lassaletta L, Gernaat DEHJ, et al. Exploring SSP land-use dynamics using the IMAGE model: Regional and gridded scenarios of land-use change and land-based climate change mitigation. Global Environmental Change. 2018 Jan 1;48:119–35. doi:10.1016/j.gloenvcha.2017.11.014

49. van Vuuren DP, Doelman JC, Schmidt Tagomori I, Beusen AHW, Cornell SE, Röckstrom J, et al. Exploring pathways for world development within planetary boundaries. Nature. 2025 May;641(8064):910–6. doi:10.1038/s41586-025-08928-w

50. Leclère D, Obersteiner M, Barrett M, Butchart SHM, Chaudhary A, De Palma A, et al. Bending the curve of terrestrial biodiversity needs an integrated strategy. Nature. 2020;585(7826):551–6. doi:10.1038/s41586-020-2705-y

51. Weindl I, Soergel B, Ambrósio G, Daioglou V, Doelman J, Beier F, et al. Food and land system transformations under different societal perspectives on sustainable development. Environ Res Lett. 2024 Nov;19(12):124085. doi:10.1088/1748-9326/ad8f46

52. Robinson S, Dunston S, Mishra A, Sulser TB, Mason-D'Croz D, Robertson RD, et al. The International Model for Policy Analysis of Agricultural Commodities and Trade (IMPACT): Model documentation for version 3.6 [Internet]. 2024 Jul 8 [cited 2024 Nov 21]. Available from: https://hdl.handle.net/10568/148953

53. van Zeist WJ, Stehfest E, Doelman JC, Valin H, Calvin K, Fujimori S, et al. Are scenario projections overly optimistic about future yield progress? Global Environmental Change. 2020 Sep 1;64:102120. doi:10.1016/j.gloenvcha.2020.102120

54. Rosegrant MW, Sulser TB, Mason-D'Croz D, Cenacchi N, Nin-Pratt A, Dunston S, et al. Quantitative foresight modeling to inform the CGIAR research portfolio [Internet]. 2017 [cited 2024 Nov 21]. Available from: https://hdl.handle.net/10568/148186

55. Beier FD, Dietrich JP, Heinke J, Abrahao G, Jeetze P von, Bodirsky BL, et al. Planetary boundaries under a land-based climate change mitigation scenario with a food demand transformation: a modelling study. The Lancet Planetary Health. 2025 Jul 8;0(0). doi:10.1016/S2542-5196(25)00087-7 PubMed PMID: 40645200.

56. Stehfest E, Zeist WJ van, Valin H, Havlík P, Popp A, Kyle P, et al. Key determinants of global land-use projections. Nature Communications. 2019 May;10(1):2166. doi:10.1038/s41467-019-09945-w

57. Springmann M, Clark M, Mason-D'Croz D, Wiebe K, Bodirsky BL, Lassaletta L, et al. Options for keeping the food system within environmental limits. Nature. 2018;562(7728):519–25. doi:10.1038/s41586-018-0594-0

58. Stevens A, Hersi M, Garritty C, Hartling L, Shea BJ, Stewart LA, et al. Rapid review method series: interim guidance for the reporting of rapid reviews. BMJ Evidence-Based Medicine. 2025 Apr 1;30(2):118–23. doi:10.1136/bmjebm-2024-112899 PubMed PMID: 39038926.

59. Sundiang M, Oliveira TD, Mason-D'Croz D, Gibson M, Beier F, Benavidez L, et al. Bundling measures for food systems transformation: a global, multimodel assessment. The Lancet Planetary Health. 2025 Oct 1;9(10). doi:10.1016/j.lanplh.2025.101339 PubMed PMID: 41197651.

60. Stehfest E, van Zeist WJ, Valin H, Havlik P, Popp A, Kyle P, et al. Key determinants of global land-use projections. Nat Commun. 2019 May 15;10(1):1. doi:10.1038/s41467-019-09945-w

61. Popp A, Calvin K, Fujimori S, Havlik P, Humpenöder F, Stehfest E, et al. Land-use futures in the shared socio-economic pathways. Global Environmental Change. 2017 Jan 1;42:331–45. doi:10.1016/j.gloenvcha.2016.10.002

62. Kyle P, Ollenburger M, Zhang X, Niazi H, Durga S, Ou Y. Assessing Multi-Dimensional Impacts of Achieving Sustainability Goals by Projecting the Sustainable Agriculture Matrix Into the Future. Earth's Future. 2023;11(10):e2022EF003323. doi:10.1029/2022EF003323

63. Emmerling J, Andreoni P, Charalampidis I, Dasgupta S, Dennig F, Feindt S, et al. A multi-model assessment of inequality and climate change. Nat Clim Chang. 2024 Oct 4;1–7. doi:10.1038/s41558-024-02151-7

64. Rogelj J, Popp A, Calvin KV, Luderer G, Emmerling J, Gernaat D, et al. Scenarios towards limiting global mean temperature increase below 1.5 °C. Nature Clim Change. 2018 Apr;8(4):325–32. doi:10.1038/s41558-018-0091-3

65. Blanchard JL, Novaglio C, Maury O, Harrison CS, Petrik CM, Fierro-Arcos D, et al. Detecting, Attributing, and Projecting Global Marine Ecosystem and Fisheries Change: FishMIP 2.0. Earth's Future. 2024;12(12):e2023EF004402. doi:10.1029/2023EF004402

66. Pereira HM, Martins IS, Rosa IMD, Kim H, Leadley P, Popp A, et al. Global trends and scenarios for terrestrial biodiversity and ecosystem services from 1900 to 2050. Science. 2024 Apr 26;384(6694):458–65. doi:10.1126/science.adn3441

67. Im U, Brandt J, Geels C, Hansen KM, Christensen JH, Andersen MS, et al. Assessment and economic valuation of air pollution impacts on human health over Europe and the United

States as calculated by a multi-model ensemble in the framework of AQMEII3. Atmospheric Chemistry and Physics. 2018 Apr 27;18(8):5967–89. doi:10.5194/acp-18-5967-2018

68. Yao Y, Ducharne A, Cook BI, De Hertog SJ, Aas KS, Arboleda-Obando PF, et al. Impacts of irrigation expansion on moist-heat stress based on IRRMIP results. Nat Commun. 2025 Jan 26;16(1):1045. doi:10.1038/s41467-025-56356-1

69. FAO. FAOSTAT Database [Internet]. 2024 [cited 2024 Oct 21]. Available from: https://www.fao.org/faostat/en/#data
